# *Salmonella* exploits the host endolysosomal tethering factor HOPS complex to promote its intravacuolar replication

**DOI:** 10.1371/journal.ppat.1006700

**Published:** 2017-10-30

**Authors:** Aastha Sindhwani, Subhash B. Arya, Harmeet Kaur, Divya Jagga, Amit Tuli, Mahak Sharma

**Affiliations:** 1 Department of Biological Sciences, Indian Institute of Science Education and Research Mohali (IISERM), Punjab, India; 2 CSIR-Institute of Microbial Technology (IMTECH), Chandigarh, India; Johns Hopkins School of Public Health, UNITED STATES

## Abstract

*Salmonella enterica* serovar *typhimurium* extensively remodels the host late endocytic compartments to establish its vacuolar niche within the host cells conducive for its replication, also known as the *Salmonella*-containing vacuole (SCV). By maintaining a prolonged interaction with late endosomes and lysosomes of the host cells in the form of interconnected network of tubules (*Salmonella*-induced filaments or SIFs), *Salmonella* gains access to both membrane and fluid-phase cargo from these compartments. This is essential for maintaining SCV membrane integrity and for bacterial intravacuolar nutrition. Here, we have identified the multisubunit lysosomal tethering factor—HOPS (HOmotypic fusion and Protein Sorting) complex as a crucial host factor facilitating delivery of late endosomal and lysosomal content to SCVs, providing membrane for SIF formation, and nutrients for intravacuolar bacterial replication. Accordingly, depletion of HOPS subunits significantly reduced the bacterial load in non-phagocytic and phagocytic cells as well as in a mouse model of *Salmonella* infection. We found that *Salmonella* effector SifA in complex with its binding partner; SKIP, interacts with HOPS subunit Vps39 and mediates recruitment of this tethering factor to SCV compartments. The lysosomal small GTPase Arl8b that binds to, and promotes membrane localization of Vps41 (and other HOPS subunits) was also required for HOPS recruitment to SCVs and SIFs. Our findings suggest that *Salmonella* recruits the host late endosomal and lysosomal membrane fusion machinery to its vacuolar niche for access to host membrane and nutrients, ensuring its intracellular survival and replication.

## Author summary

Intracellular pathogens have devised various strategies to subvert the host membrane trafficking pathways for their growth and survival inside the host cells. *Salmonella* is one such successful intracellular pathogen that redirects membrane and nutrients from the host endocytic compartments to its replicative niche known as the *Salmonella*-containing vacuole (SCV) via establishing an interconnected network of tubules (*Salmonella*-induced filaments or SIFs) that form a continuum with the SCVs. How *Salmonella* ensures a constant supply of endocytic cargo required for its survival and growth remained unexplored. Our work uncovers a strategy evolved by *Salmonella* wherein it secretes a bacterial effector into the host cytosol that recruits component of host vesicle fusion machinery-HOPS complex to SCVs and SIFs. HOPS complex promotes docking of the late endocytic compartments at the SCV membrane, prior to their fusion. Thus, depletion of HOPS subunits both in cultured cell lines as well as a mouse model inhibits *Salmonella* replication, likely due to reduced access to host membranes and nutrients by the vacuolar bacteria. These findings provide mechanistic insights into how this pathogen reroutes the host’s endocytic transport towards its vacuole, ensuring its own intracellular survival and replication.

## Introduction

*Salmonella enterica* serovar *typhimurium* (hereafter *Salmonella*) is a Gram-negative facultative intracellular pathogen that causes gastroenteritis in a human host and a typhoid-like disease in mice. *Salmonella* replicates inside the non-phagocytic and phagocytic mammalian host cells in a unique membrane-bound vacuolar compartment known as the *Salmonella*-containing vacuole or SCV. Modulation of SCV association with the host endocytic machinery and reorganization of the host late endosomes and lysosomes is a major virulence strategy used by this pathogen. *Salmonella* invasion into the host cell and its replication inside the SCV is facilitated by bacterial effector proteins translocated into the host cytosol by its two type III secretion systems (T3SS)-1 and (T3SS)-2, encoded by the *Salmonella* pathogenicity island (SPI)-1 and -2 respectively [1,2,3].

During early time points of infection, SCV acquires markers of early endosomes including Rab5, EEA1 (Early endosome antigen 1), SNX1, and PI(3)P [4,5,6]. Within 30–60 min post infection (p.i.), early SCV matures into late SCV by loss of early endosomal proteins and simultaneous acquisition of selective late endosomal and lysosomal proteins including Rab7, lysosomal glycoproteins (lgps) such as, LAMP1 and LAMP2 and v-ATPases [5,7]. Although the SCV acquires characteristics of late endocytic compartments including acidification, it does not become bactericidal, due to reduced presence of lysosomal hydrolyases [8]. Onset of bacterial replication in host cells begins at 3–4 hr p.i. and coincides with the formation of a tubular membrane network that emanate from the SCV, known as *Salmonella*-induced filaments (SIFs) [9,10,11]. SIFs have been observed in both *Salmonella* infected epithelial cells and phagocytic cells, and are characterized by presence of lgps such as LAMP1 [12]. Recent studies have shown that early SIFs formed during 6–8 hr p.i. are highly dynamic structures that continuously acquire content from the late endosomes and lysosomes of the host cell [10,13,14]. Detailed ultrastructure analysis of SIFs has revealed that a subset of these are double membrane structures wherein the space (that harbors the bacteria) between the outer and the inner lumen is accessible to content from the host late endosomes and lysosomes [13]. Notably, this crosstalk with the host’s endocytic compartments is essential for supply of nutrients to the SCV. As previously reported, auxotrophic strains of *Salmonella* acquire external amino acids by inducing SIF formation and redirecting host vesicular transport to the SIFs and SCV membranes [14,15]. Moreover, as SIFs are a large interconnected network of tubules forming a continuum with SCVs, this is proposed to rapidly dilute the antimicrobial activities transferred to the bacterial vacuole upon content mixing with the host late endosomes and lysosomes, preventing degradation of the vacuolar *Salmonella* [14]. These studies signify a crucial role of *Salmonella* effectors that mediate SCV interaction with the host endocytic machinery and SIF formation required for the survival and replication of this pathogen within its intravacuolar niche.

Several T3SS-2 effectors including SifA, SseJ, SseG, SseF, SopD2, and PipB2 contribute to SCV maturation, vacuole integrity and SIF formation [16]. The most severe phenotype on the intracellular *Salmonella* growth is observed with strains lacking SifA that are highly attenuated in systemic infection and replication [17,18]. Functionally, SifA regulates the integrity of SCV and is essential for SIF formation [19]. SifA interacts with the host protein SKIP (SifA and Kinesin interacting protein)/PLEKHM2 (Pleckstrin homology and RUN domain containing protein M2) that in turn bind to the motor protein, kinesin-1 [8,20,21]. SifA-mediated SKIP recruitment on SCVs is thought to relieve the auto-inhibition of kinesin motor, which in turn promotes the microtubule-dependent extension of SIFs [22]. SifA also interacts with the host protein PLEKHM1 (Pleckstrin homology and RUN domain containing protein M1), which has similar domain architecture as SKIP, and regulates membrane biogenesis of the SCV compartment, and intracellular *Salmonella* proliferation [23]. Although components of the host late endosome-lysosome fusion machinery are known to localize to SCV and SIFs (such as Rab7 [24] and Arf-like (Arl) GTPase8b [25]), but their function in *Salmonella* replication and whether *Salmonella* modulates their recruitment for its own survival needs further exploration.

Lysosome fusion with other membrane-bound compartments requires the small GTPases Rab7 and Arl8b and their effectors; PLEKHM1 and tethering/docking factor HOPS complex, respectively as well as the SNARE proteins [26,27,28,29,30,31,32]. HOPS complex is an evolutionarily conserved multisubunit tethering complex (MTC) that mediates lysosome fusion with late endosomes, phagosomes, and autophagosomes [33]. HOPS is a hexameric complex where four of the six subunits namely, Vacuole protein sorting (Vps)11, Vps16, Vps18 and Vps33a form the core complex, and Vps39 and Vps41 are the accessory subunits [34]. The four core subunits of the HOPS complex are shared with CORVET (class C core vacuole/endosome tethering), an early endosomal MTC. Vps3/TGFBRAP1 (Transforming Growth Factor Beta Receptor Associated Protein 1)/TRAP1 and Vps8 are the accessory subunits of the hexameric CORVET complex [35,36]. In yeast and in mammalian cells, CORVET complex is recruited on to the early endosomal membranes by TGFBRAP1 binding to early endosomal Rab protein, Rab5 [37,38]. In yeast, but not in mammalian cells, Rab7 directly binds to the accessory subunits Vps39 and Vps41 to recruit HOPS complex to the vacuolar membranes, promoting their homotypic fusion [27,39]. Interestingly, in metazoans including *C*. *elegans* and in mammalian cells, Vps41 subunit of the HOPS complex binds to Arl8b, which then mediates assembly of HOPS complex on lysosomes [27,28,40]. Fewer studies have explored the role of mammalian HOPS subunits in maturation of pathogen-containing vacuole. Previous work has shown that HOPS complex plays an inhibitory function in regulating intracellular survival of the pathogen *Coxiella burnetti* by mediating fusion of bacterial phagosomes with lysosomes [41]. Consequently, *C*. *burnetti-*mediated phosphorylation of Vps41 subunit of the HOPS complex prevents its membrane localization, and thereby, its function in phagolysosome fusion. HOPS complex has also been shown to be essential for Ebola virus replication with loss of HOPS subunit expression preventing viral escape to the cytosol from host’s late endosomes/lysosomes [42,43].

Although SCV compartment is known to acquire content from the late endocytic compartments of the host cell [9,12], little is known if *Salmonella* employs HOPS complex to mediate fusion with host endolysosomes. Previous studies have shown that HOPS subunit Vps39 interacts with SKIP/PLEKHM2 and PLEKHM1, both of which bind to the *Salmonella* effector SifA [27,29]. Moreover, HOPS complex is an effector of the small GTPase Arl8b that localizes to SCVs and SIFs in *Salmonella*-infected HeLa cells [25,27]. A more direct evidence of HOPS function during *Salmonella* infection was shown where depletion of HOPS subunits (similar to PLEKHM1 depletion) altered SCV morphology with multiple bacteria present within a single enlarged vacuole [23]. However, *Salmonella* infection in these experiments was visualized after 20 hr p.i. while SCV interaction with host late endosomes/lysosomes and SIF formation is observed as early as 6 hr p.i. [9]. Thus, with regard to the role of HOPS complex in *Salmonella* infection, several important questions remain unanswered, for instance, whether HOPS complex regulates *Salmonella* replication, does it regulate SCV maturation and SIF formation and what are the bacterial and host factors required for recruitment of HOPS complex to SCVs and SIFs.

Here, we demonstrate an essential role of HOPS complex in mediating intracellular *Salmonella* replication in non-phagocytic and phagocytic cells and in a mouse model of *Salmonella* infection. Live-cell imaging experiments and transmission electron microscopy (TEM) studies revealed that SIF formation and fusion of mature SCVs with late endosomes and lysosomes were severely compromised upon depletion of HOPS subunits. Consequently, nutrient access to SCVs from the host late endocytic compartments was also impaired upon HOPS depletion. Notably, we found that bacterial effector SifA, in complex with the host protein SKIP, interact with HOPS complex and mediate HOPS localization to SCVs, enabling fusion with LEs/Lysosomes. Surprisingly, we did not find a role PLEKHM1 in mediating HOPS recruitment to SCVs although previous studies had shown that it independently binds to both SifA and Vps39 [23,29]. In conclusion, our results demonstrate that *Salmonella* recruits the host vesicle fusion machinery to gain access to nutrients and membranes from the late endocytic compartments to build its replicative niche inside the host cells.

## Results

### HOPS subunits are recruited to LAMP1-positive SCVs and SIFs during *Salmonella* infection

**Fig 1:**
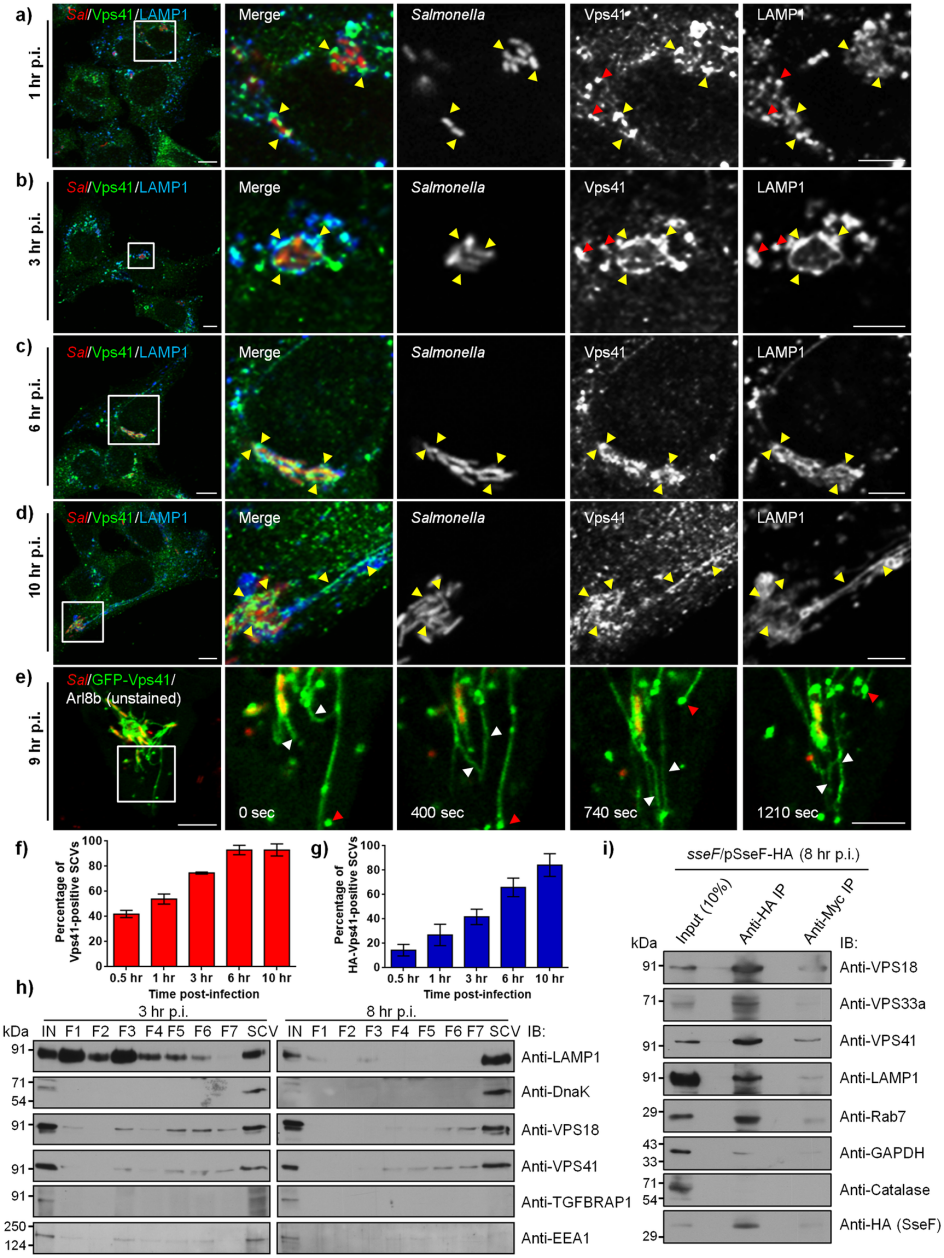
HOPS subunits are recruited to LAMP1-positive SCVs and SIFs during *Salmonella* infection. **a-d)** Representative confocal micrographs of HeLa cells infected with DsRed-expressing *Salmonella* (red). At different time points post infection (p.i.), cells were fixed and stained for endogenous Vps41 (green) and LAMP1 (blue). Different panels represent a higher magnification of the boxed areas, showing recruitment of Vps41 on SCVs and SIFs (marked by yellow arrowheads). Red arrowheads indicate lysosomal localization of Vps41 in panels (**a**) and (**b**). Bars: (main) 10 μm; (insets) 5 μm. **e)** Time-lapse microscopy of HeLa cells co-transfected with plasmids encoding GFP-Vps41 and untagged-Arl8b, and infected with DsRed-expressing *Salmonella* (red). Time-lapse series were recorded 9 hr p.i., and still images shown here correspond to S2 Movie. Different panels represent a higher magnification of the boxed area indicating Vps41-positive SIFs emanating from the SCVs showing extension, retraction and bifurcation (white arrowheads). Red arrowheads indicate fusion of Vps41-positive vesicles with SIFs. Bars: (main) 10 μm; (insets) 5 μm. **f and g)** Quantification of endogenous (**f**) or HA-tagged Vps41 (**g**)-positive SCVs at different time points p.i. Data represent percentage of Vps41-postive SCVs scored for ~100 SCVs for each time point. The mean ± S.D. is shown for three independent experiments. **h)** SCVs were isolated from *Salmonella*-infected HeLa cells at 3 hr and 8 hr p.i. using sucrose density ultracentrifugation, followed by second round of ultracentrifugation of fractions 8–10 on a ficoll cushion (labeled as SCV). Different fractions were resolved on SDS-PAGE gel and immunoblotted using indicated antibodies. **i)** *Salmonella*-modified membranes were isolated from HeLa cells infected with *sseF*-deficient strain of *Salmonella* harboring an expression vector with a C-terminal epitope-tagged *sseF* and its cognate chaperone *sscB* (*sseF*/pSseF-HA) at 8 hr p.i. by differential centrifugation. The enriched fraction was further subjected to affinity immunoprecipitation (IP) using anti-HA antibody-conjugated agarose beads or anti-Myc antibody-conjugated agarose beads as a control. The eluted samples were analyzed for presence of effector protein (SseF) and host proteins by Western blotting as indicated.

To investigate the role of HOPS complex in SCV maturation and fusion with late endosomes/lysosomes, we first examined the time-dependent localization of HOPS subunits to SCVs and SIFs in *Salmonella*-infected human epithelial cell line (HeLa). Vps41 that recruits other subunits of the HOPS complex to lysosomes [27], showed weak association with early SCVs at 10 min p.i. Most SCVs were positive for the early endosomal marker-EEA1 at this time point (S1a Fig). Recruitment of HOPS subunits, Vps41 and Vps18, around the SCVs was observed starting at 1 hr p.i. (53±4% for Vps41) that became more evident by 3 hr p.i. where 74±1% SCVs were positive for Vps41 (Fig 1a, 1b and 1f; S1b and S1c Fig). By these time points, SCVs undergo maturation and as shown in the images acquire late endosomal/lysosomal marker- LAMP1 (Fig 1a and 1b; S1b and S1c Fig). Epitope-tagged Vps41 and Vps33a were similarly recruited to the mature SCVs in *Salmonella*-infected HeLa cells that were briefly treated with mild-detergent prior to fixation to remove the cytosolic signal of the overexpressed proteins (S1f–S1i Fig; quantification of HA-Vps41 SCVs shown in Fig 1g). Prior treatment with detergent resulted in non-specific nuclear staining, as observed in confocal micrographs of the transfected cells (S1f–S1i Fig). Notably, endogenous and epitope-tagged HOPS subunits also localized to LAMP1-positive vesicles, supporting their reported subcellular distribution to late endosomes/lysosomes (see red arrowheads in insets of Fig 1a and 1b). We were unable to observe the subcellular localization of the HOPS-specific subunit-Vps39 in these experiments, due to lack of an antibody against the endogenous protein and the fact that its overexpression results in striking coalescence of lysosomes into large aggregates [44]. Previous studies have shown that four of the six subunits of the HOPS complex (Vps11, Vps16, Vps18 and Vps33a) are shared with CORVET, which is an early endosomal tethering factor [35]. To determine whether CORVET complex also localizes to SCV membranes, we analyzed distribution of epitope-tagged CORVET specific subunit-TGFBRAP1 in *Salmonella*-infected cells at 10 min, 30 min, 1 hr, 3 hr and 6 hr p.i. (S2a–S2e Fig). As expected, TGFBRAP1 colocalized with EEA1 (see yellow arrowheads in insets of S2a Fig) but not LAMP1. Further, little or no recruitment of TGFBRAP1 on SCVs and SIFs was observed at different time points of infection (S2a–S2e Fig).

Beginning at 6 hr, but better visualized at 10 hr p.i., HOPS subunit Vps41 localized to >93±4% SCVs and also localized to SIFs, identified by co-immunostaining for LAMP1-a well-characterized marker for these tubular membranes that frequently extend from the surface of mature SCVs (Fig 1c and 1d). We also observed a similar striking localization of Vps18 subunit of the HOPS complex to SCVs and SIFs in infected cells beginning at 6 hr, but primarily at 10 hr p.i. (S1d and S1e Fig). Localization of HOPS subunits to SIFs was also verified by expressing epitope-tagged-Vps41 and -Vps33a in *Salmonella*-infected cells (S1j–S1m Fig). We observed that localization of Vps41 appeared to be discontinuous and in discrete domains along the length of the SIFs. Previous studies have described similar discrete distribution of LAMP1 on SIFs attributed to poor preservation of the tubular membranes in fixed cells [10,16]. To elucidate whether the punctate localization of HOPS subunits observed on the SIFs was due to fixation, we infected HeLa cells with *Salmonella* constitutively expressing monomeric DsRed (DsRed-*Salmonella*) followed by transfection with GFP-tagged Vps41 at 2 hr p.i. Live-cell imaging at 9–10 hr p.i. revealed Vps41 was present around the SCVs and on SIFs in a continuous manner rather than as discrete domains (S2k Fig and S1 Movie). To reduce the cytosolic signal that interfered with visualizing the membrane localization of overexpressed Vps41, we co-expressed small GTPase Arl8b, which recruits Vps41 to lysosomes (Fig 1e and S2 Movie) [27]. Moreover, as shown in a previous study [25] and as can be appreciated in S2f–S2j Fig, Arl8b itself localizes to SCVs starting at 1 hr p.i., and to SIFs at 6 hr and 10 hr p.i., and is an excellent marker to visualize these compartments. Notably, we found that GFP-Vps41 was completely cytosolic and failed to localize to SCVs in CRISPR/Cas9 Arl8b-knockout cells (S2l and S2m Fig and S3 Movie). Quantification of Vps41-positive SCVs at 10 hr p.i. in wild type (WT)- and Arl8b knockout-HeLa cells demonstrated an essential role of Arl8b in recruitment of HOPS complex to SCV membranes. (S2n Fig; mean percentage Vps41-positive SCVs in WT: 91±2% and Arl8b KO: 6±1%). Consistent with this role of Arl8b, we found a striking recruitment of GFP-tagged Vps41 to SCVs and SIFs in Arl8b co-expressing cells where Vps41 was present around the SCVs and SIFs in a continuous manner, with fewer Vps41-positive vesicles remaining in the cell (Fig 1e and S2 Movie). We also observed the dynamic extension and retraction of Vps41-labeled SIFs in these cells (see white arrowheads in Fig 1e and S2 Movie). Further, Vps41-positive vesicles were also observed to fuse with the existing tubules and vesicles that were moving along the length of the tubules (see red arrowheads in Fig 1e).

Next, we examined whether recruitment of HOPS subunits to *Salmonella*-associated membranes increased as a function of time. To analyze this, we resolved the *Salmonella*-infected homogenates by two-step density gradient ultracentrifugation and confirmed the presence of *Salmonella* by immunoblotting with antibodies against the bacterial protein-DnaK (Fig 1h, fractions 8–10 (labeled as SCV fraction)). Comparison of the *Salmonella*-infected cell homogenates processed at 3 hr and 8 hr p.i. demonstrated that endogenous HOPS subunits along with LAMP1 (a known SCV marker) were enriched in the SCV fraction from 3 hr to 8 hr p.i. (Fig 1h). As expected, the early endosomal marker-EEA1 was associated with the SCVs at 3 hr p.i. but not at 8 hr p.i. Similarly, CORVET-specific subunit- TGFBRAP1 was weakly associated with SCV fractions at 3 hr p.i. but not at 8 hr p.i., supporting our earlier results of little or no recruitment of TGFBRAP1 to early SCVs (Fig 1h). To verify HOPS enrichment on late SCVs and SIFs, we also employed a recently described method of SCV isolation by immunoprecipitation (IP) of SseF-an integral membrane SPI2-T3SS effector protein [45]. As shown in Fig 1i, HOPS subunits were specifically enriched in the SseF-IP eluate but not control IP with levels comparable to the known SCV markers, such as LAMP1 and Rab7. In contrast, little or no co-IP of GAPDH or Catalase with SseF was observed, substantiating the specificity of this approach for SCV isolation (Fig 1i). Our results indicate correlation between recruitment of HOPS complex with time points wherein SCV is known to acquire content from late endosomes and lysosomes [9]. Indeed, in a recent study by Santos et al., where proteomes of early SCV and late SCV were compared, enrichment of HOPS subunits Vps11, Vps16, and Vps18 was observed in the late SCV fractions [46].

### HOPS complex is required for intracellular *Salmonella* replication

**Fig 2:**
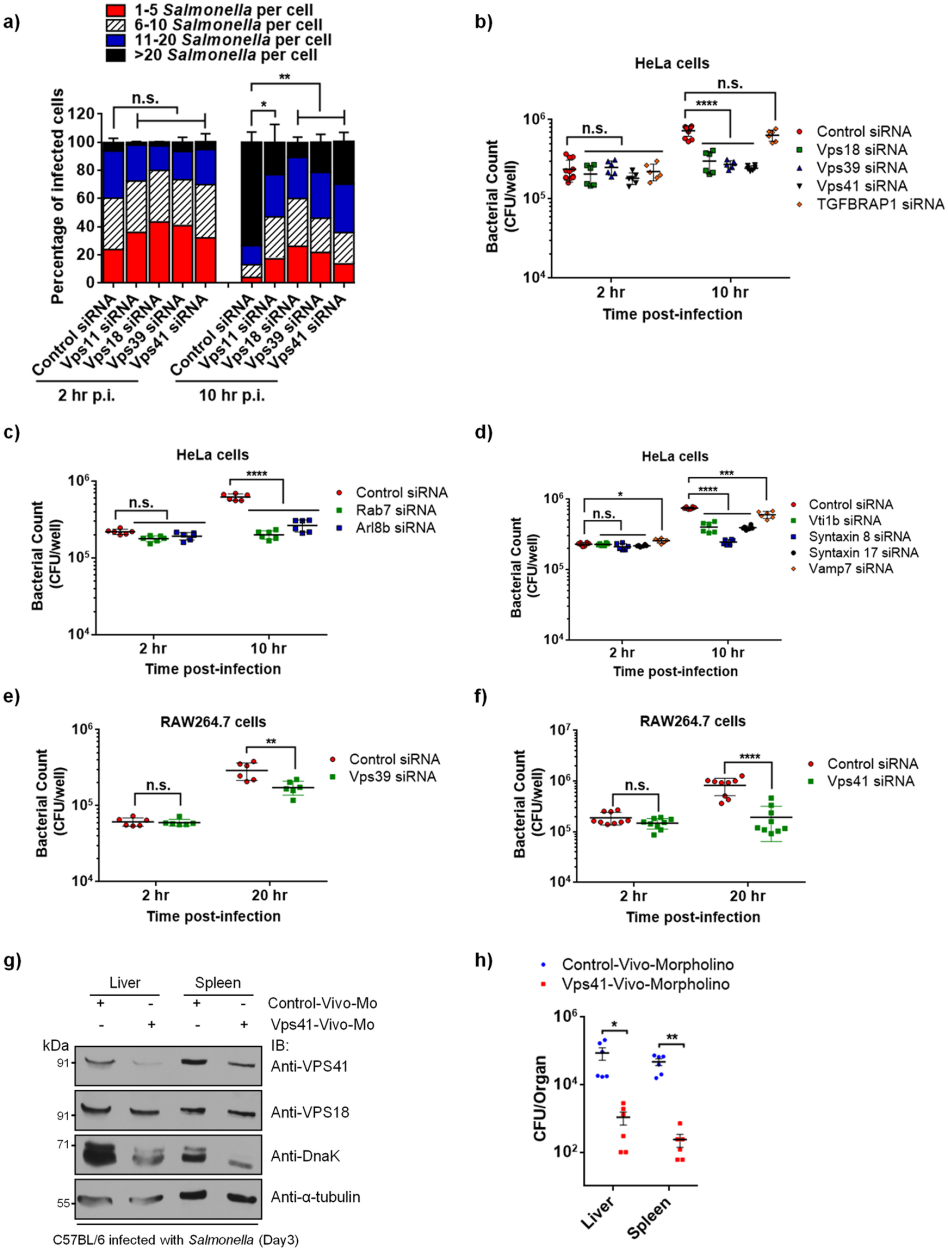
Depletion of HOPS subunits impairs *Salmonella* replication. **a)** HeLa cells transfected with indicated siRNA were infected with *Salmonella*, fixed at the indicated time points, and immunostained with antibodies to *Salmonella* and LAMP1. Using confocal microscopy, the number of intracellular bacteria was enumerated in ~300 cells per experiment. These numbers were grouped according to the legend, and expressed as a percentage of the total infected cell population. The mean ± S.D. is shown for three independent experiments (n.s., not significant; *, P < 0.05; **, P < 0.01; Student’s *t* test). **b-f)** Intracellular replication assay. HeLa (**b-d**) and RAW264.7 (**e** and **f**) cells treated with indicated siRNA and infected with *Salmonella* were harvested at indicated times p.i. The number of colony forming units (CFU) per well are shown as dot plot and data represent mean ± S.D. (n.s., not significant; *, P < 0.05; **, P < 0.01; ***, P < 0.001; ****, P < 0.0001; Student’s *t* test). **g and h)** Mice were injected intravenously (i.v.) with control- or Vps41 specific-vivo-morpholinos (sample size of six for each treatment) for 2 days at an interval of 24 hr, followed by an i.v. challenge with 1.3 × 10^3^ CFU *Salmonella*. On day 3 p.i., mice were sacrificed and bacterial loads in spleen and liver were determined by plating serial dilutions of tissue homogenates on agar plates containing streptomycin (*, P < 0.05; **, P < 0.01; Student’s *t* test). The tissue homogenates were resolved on SDS-PAGE and immunoblotted with indicated antibodies by Western blotting.

To elucidate the significance of HOPS complex during *Salmonella* infection, we assessed the intracellular replication of *Salmonella* in cells depleted of various HOPS subunits. Western blotting and qRT-PCR analysis confirmed efficient depletion of HOPS subunits in HeLa cells (S3a–S3e Fig). Control- and HOPS specific-siRNA treated HeLa cells were infected with *Salmonella* and fixed at 2 hr and 10 hr p.i., and labeled with anti-*Salmonella* antibodies to enumerate the intracellular bacterial load by immunofluorescence microscopy (Fig 2a). At 2 hr p.i., both control- and HOPS-siRNA treated cells showed a similar bacterial load with ~35% cells containing 6–10 bacteria/cell, ~17–30% of cells containing 11–20 bacteria/cell, and ~2–7% of cells were containing >20 bacteria/cell. These results suggest that HOPS complex is not required for *Salmonella* invasion into the host cells (Fig 2a). In contrast to this early time point of infection, at 10 hr p.i., while ~73% of control siRNA treated cells had >20 bacteria/cell and <13% had 11–20 bacteria/cell, only 10–30% of HOPS siRNA treated cells showed a similar bacterial load with almost equal distribution of cells containing either 6–10 bacteria/cell (20–30%) or 11–20 bacteria/cell (30–35%) (Fig 2a). These results indicate a severe defect in bacterial replication upon depletion of HOPS subunits.

We corroborated these observations by determining the number of Colony Forming Units (CFUs) present in control- and HOPS siRNA-treated HeLa cell lysates at 2 hr and 10 hr p.i. As shown in Fig 2b (quantification of CFUs/well), we observed a ~3 fold increase in bacterial replication in control cells, while only ~1.09–1.4 fold increase was observed in HOPS-depleted cells. Consistent with our previous data depicting weak or no association of CORVET subunit TGFBRAP1 with SCVs and SIFs, a ~2.8 fold increase in bacterial replication was observed upon TGFBRAP1 depletion (Fig 2b and S3f Fig showing knockdown efficiency >70%), which was not significantly different from control cells. These results suggest that HOPS, but not CORVET complex, regulates intracellular *Salmonella* replication. Since HOPS complex is one of the components of the late endocytic vesicle fusion machinery, we compared bacterial burden in HOPS-depleted cells with cells depleted of small GTPases and SNARE proteins also required for late endosome-lysosome fusion. To this end, we analyzed bacterial replication in cells treated with siRNA against Rab7, Arl8b, and late endosomal/lysosomal SNAREs proteins-Vti1b, Syntaxin 8, Syntaxin 17, and Vamp7. Western blotting and qRT-PCR analysis were done to confirm efficient depletion of these proteins in HeLa cells (S3g–S3l Fig). Similar to HOPS depletion, only ~1.1–1.3 fold increase in bacterial replication from 2 hr to 10 hr was observed in Rab7 and Arl8b depleted cells (Fig 2c). Amongst SNAREs, Syntaxin8 showed the most significant decrease in bacterial replication (~1.15 fold; Fig 2d) followed by Vti1b and Syntaxin 17 (~1.75 and ~1.8 fold, respectively; Fig 2d) whereas bacterial replication was modestly (but significantly) decreased in Vamp7-depleted cells (~2.3 fold; Fig 2d). In addition to HeLa cells, we also verified that HOPS subunit-Vps41 is required for *Salmonella* replication in primary mouse embryonic fibroblasts, MEFs (S3p and S3r Fig; knockdown efficiency >70%; control siRNA: ~3.3 fold, Vps41 siRNA: ~2.5 fold increase in bacterial burden from 2 hr to 10 hr p.i.).

It is well understood that macrophages are the major reservoir of *Salmonella* in host organisms [47]. Accordingly, to determine whether HOPS complex is required for *Salmonella* replication in macrophages, we performed CFU assays in control-, Vps39- and Vps41-siRNA treated macrophage-like cell line-RAW264.7, a well-established cell line model for *in vitro* studies of *Salmonella* infection (S3m and S3n Fig; knockdown efficiency >80%). As compared to control where ~4 fold increase in bacterial burden was observed, we found only a ~2.8 fold and ~1.3 fold increase in *Salmonella* burden upon Vps39 and Vps41 depletion, respectively, in macrophages reinforcing that HOPS complex is essential host factor for intracellular *Salmonella* replication in both epithelial and macrophage cells (Fig 2e and 2f). A similar trend (but overall less replication) was observed in the control and Vps41 lentiviral-mediated shRNA transduced cells (S3o and S3q Fig; knockdown efficiency >70%; control shRNA: ~2 fold and Vps41 shRNA: ~ 0.9 fold change in bacterial burden from 2 hr to 10 hr p.i.) To corroborate the bacterial infection experiments performed under *in vitro* cell culture conditions, we next assessed whether HOPS subunits are required for *in vivo* replication of *Salmonella* in a mouse model. To determine this, we used morpholino-based approach to downregulate Vps41 expression in mice that were further infected with *Salmonella* by intravenous injection. As a control, standard negative control morpholino was injected in age-matched mice. At day 3 p.i., CFU counts were analyzed from the liver and spleen homogenates of control- and Vps41-morpholino treated mice. The efficiency of Vps41 depletion in both liver and spleen was found to be >80% and >70%, respectively, while no change in the levels of Vps18 (that directly binds to Vps41) was observed (Fig 2g). Similar to our previous findings in cultured cells, striking decrease in *in vivo* replication of *Salmonella* was observed upon Vps41 depletion (Fig 2h). Consistent with this, DnaK signal was also strikingly reduced in tissue homogenates from Vps41 morpholino-injected mice (Fig 2g, *third panel*). Overall, our findings reveal HOPS complex as an essential host factor required for *Salmonella* proliferation in multiple cell types and in a murine infection model.

### Depletion of HOPS subunits delays but does not block SCV maturation and LAMP1 acquisition

**Fig 3:**
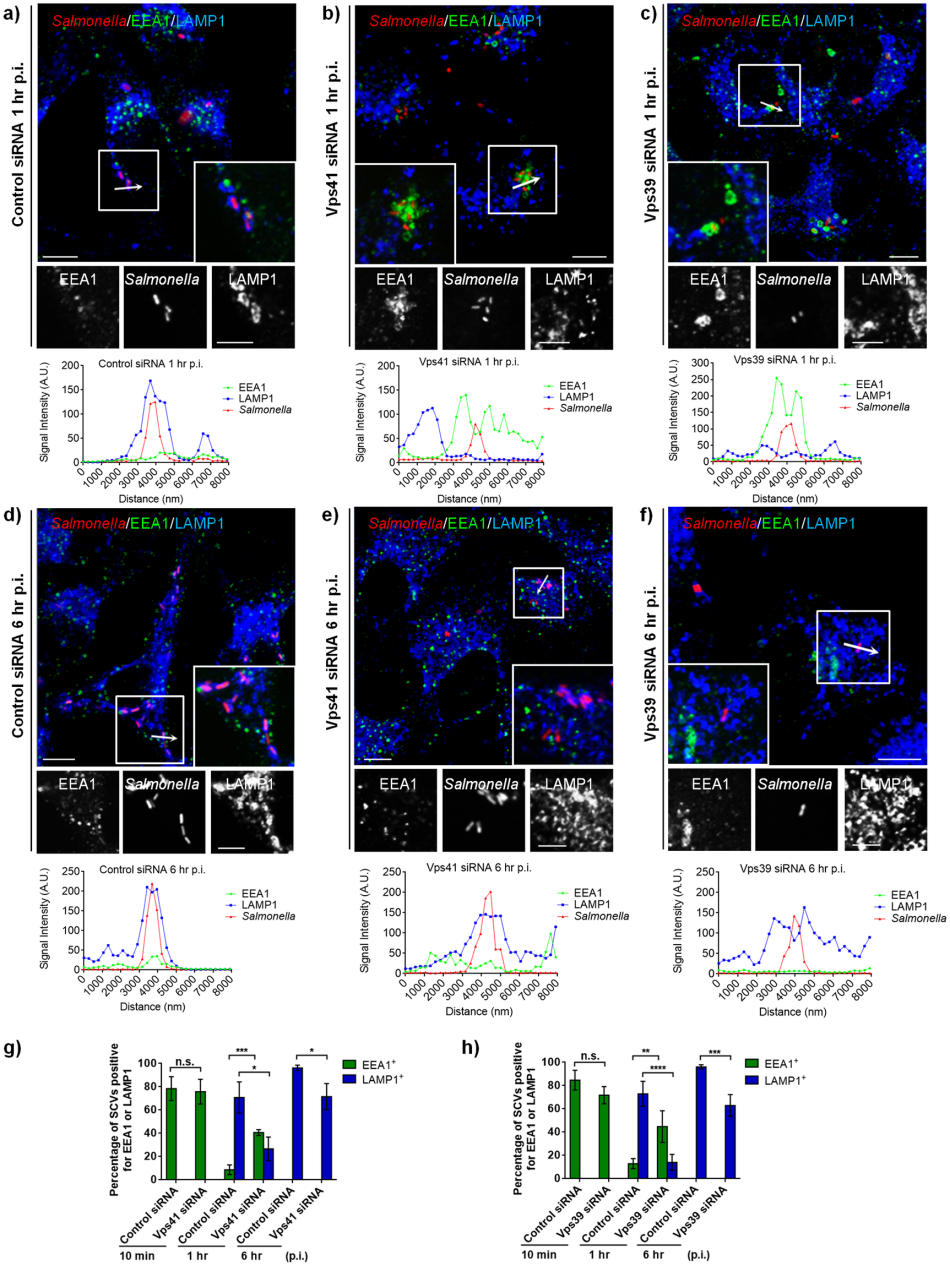
Depletion of HOPS subunits delays but does not block SCV maturation. **a-f)** Representative confocal micrographs of control siRNA-, Vps41 siRNA- or Vps39 siRNA-treated HeLa cells infected with DsRed-expressing *Salmonella* (red). At different time points p.i., cells were fixed and stained for early endosomes marker, EEA1 (green), and LAMP1 (blue). Insets depict higher magnification of the boxed areas showing localization of different markers on the SCVs. Intensity line scan profile of EEA1/LAMP1 across the width of a single SCV (indicated by an arrow in the boxed region) is shown below the individual image. Bars: (main) 10 μm; (insets) 5 μm. **g and h)** Quantification of percentage of infected cells displaying EEA1/LAMP1-accumulation around SCVs at the indicated time point p.i. Data represent mean ± S.D. for ~50 SCVs from three independent experiments (n.s., not significant; *, P < 0.05; **, P < 0.01; ***, P < 0.001; ****, P < 0.0001; Student’s *t* test).

**Fig 4:**
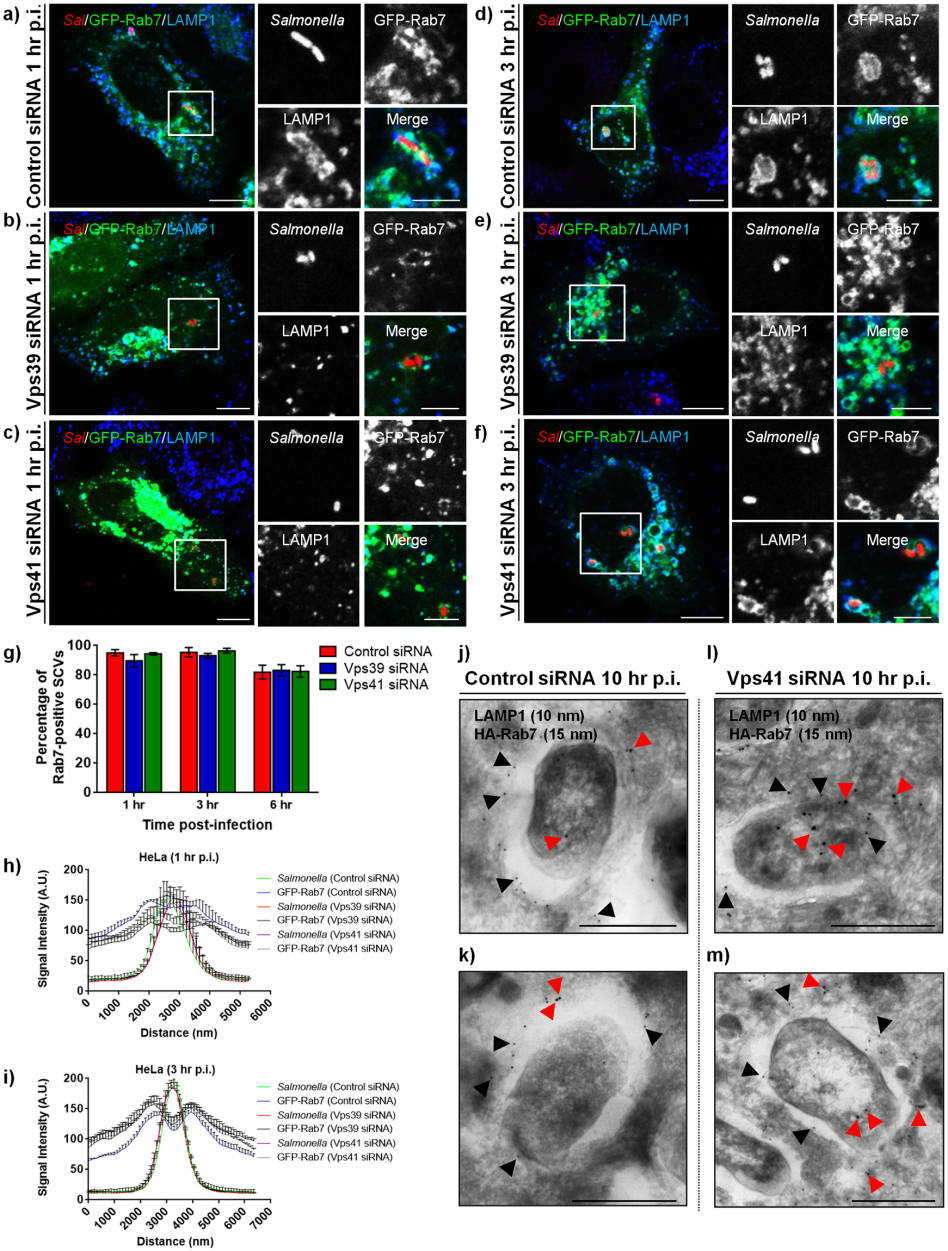
Depletion of HOPS subunits does not alter Rab7 recruitment to SCV. **a-f)** Representative confocal images of control-, Vps41-, or Vps39-siRNA treated HeLa cells, and subsequently transfected with GFP-Rab7 and infected with DsRed-expressing *Salmonella* (red). At different times p.i., cells were fixed and stained for LAMP1 (blue). Insets depict higher magnification of the boxed areas showing localization of Rab7 and LAMP1 around the SCVs. Bars: (main) 10 μm; (insets) 5 μm. **g)** Quantification of Rab7-positive SCVs in control-, Vps41- or Vps39-siRNA treated HeLa cells. Data represent mean ± S.D. over three independent experiments at the indicated time points where ~100 SCVs were counted in each experiment. **h and i)** Quantification of GFP-Rab7 intensity around the SCVs in control-, Vps41- or Vps39-siRNA treated cells over three independent experiments at the indicated time points p.i. where intensity profile of ~50 SCVs were quantified in each experiment. Data represent mean ± S.E.M. **j-m)** Representative immunogold EM images of control siRNA (**j** and **k**)- or Vps41 siRNA (**l** and **m**)-treated HeLa cells infected with *Salmonella* for 2 hr and transfected with HA-tagged Rab7 and fixed at 10 hr p.i. Cells were processed for immunogold labeling with anti-LAMP1 (10 nm) and anti-HA (15 nm) antibodies. Arrowheads indicate localization of Rab7 (red) and LAMP1 (black) around the SCVs. Bar: 500 nm.

To establish its intracellular replicative compartment, *Salmonella* dynamically interacts with, and acquires both membrane and luminal content from host late endosomes/lysosomes [2]. Since HOPS complex is a crucial factor required for tethering and fusion of incoming cargo with lysosomes, we hypothesized that HOPS function is required for SCV fusion with late endosomes and lysosomes. To this end, we first analyzed SCV maturation upon HOPS depletion by quantifying recruitment of early SCV marker (EEA1) and late SCV marker (LAMP1) at different time points in control-, Vps41- and Vps39-siRNA treated cells. At 10 min p.i., no significant differences in the percentage of EEA1-positive SCVs were evident in HOPS-depleted cells as compared to the control cells (S4a–S4c Fig and quantification shown in Fig 3g and 3h; control siRNA: ~78–84%, Vps41 siRNA:~75%, and Vps39 siRNA: ~71%). LAMP1 acquisition was not observed in either control or HOPS depleted cells at this early time point of infection (S4a–S4c Fig; see intensity profile). At 1 hr p.i., ~70% of SCVs in control siRNA treated cells were now EEA1-negative and had acquired LAMP1 (Fig 3a, see intensity profile; quantification shown in Fig 3g and 3h). In contrast, upon HOPS depletion, we found that EEA1 was still retained around ~40% of SCVs while LAMP1 acquisition was observed only around ~14–25% SCVs at 1 hr p.i. (Fig 3b and 3c; quantification shown in Fig 3g and 3h). These findings suggest a delay in SCV maturation upon depletion of HOPS subunits. Interestingly, by 6 hr p.i., ~62–70% of SCVs had acquired LAMP1 staining in HOPS siRNA treated cells and none were found to be positive for the early endosomal marker, EEA1 (Fig 3d–3f; quantification shown in Fig 3g and 3h). As LAMP1 is distributed on both late endosomes and lysosomes, we also analyzed localization of a specific late endosomal markers-Rab7 and -LBPA on SCVs in control and HOPS depleted cells at 1–6 hr p.i. As previously reported [48], we did not observe acquisition of the late endosomal lipid-lysobisphosphotidic acid (LBPA) to SCV membranes either in control or HOPS depleted cells at 1 hr and 6 hr p.i. (S5 Fig). Interestingly, Rab7 acquisition was unchanged upon HOPS depletion wherein >80–90% SCVs were positive for Rab7 at 1 hr, 3 hr, and 6 hr p.i in both control and HOPS depleted cells (Fig 4a–4f; quantification shown in Fig 4g). Notably, as compared to the control siRNA treated cells, we did observe a modest decrease in Rab7 intensity around the SCVs in HOPS depleted cells at 1 hr p.i. that was recovered by 3 hr p.i. (Fig 4h and 4i). Our findings suggest that especially at 1 hr p.i., several SCVs in HOPS-depleted cells retain characteristics of both early endosomes and late endosomes. (see quantification shown in Figs 3g, 3h and 4g). Taken together, these results signify a delay but not a complete block in SCV maturation upon depletion of HOPS subunits.

Our findings indicate that SCV maturation follows a scheme similar to maturation of early endosomes to multi-vesicular bodies/late endosomes upstream of HOPS-mediated fusion of late endosomes and lysosomes [49,50]. In agreement with this, previous studies have shown that endocytic machinery required for early to late endosome maturation such as Vps34 and Rab7 is also required for SCV maturation [24,51,52]. To confirm that LAMP1 acquisition by SCVs is not inhibited upon fusion with lysosomes, we treated cells with Bafilomycin A1 (Baf A1), a routinely used chemical inhibitor of vesicle fusion with lysosomes [53]. Baf A1 inhibits fusion of lysosomes with other compartments by inactivating the ER Ca^2+^-ATPase (SERCA) whose activity is required to maintain the lysosomal Ca^2+^ stores [54,55]. As shown in S4d and S4e Fig, LAMP1 acquisition around SCVs was not impaired in cells pretreated with Baf A1 (see intensity profile graphs in S4f and S4g Fig) although SIF formation was abrogated in the presence of this drug. These findings support our conclusion that LAMP1 acquisition by SCV does not require heterotypic fusion with lysosomes, which in turn is mediated by HOPS complex.

Previous studies have shown that *Salmonella* colonizes and hyper-replicates within the cytosol of epithelial cells [56,57]. To address whether the cytosolic hyper-replicating *Salmonella* population is increased upon HOPS depletion, we determined bacterial burdens in control and Vps41 depleted cells using the previously described modified gentamicin protection assay where cells are treated with chloroquine (CHQ) before the end of infection time point. CHQ is a lysosomotrophic agent that accumulates within endosomes/lysosomes and has been shown to degrade vacuolar but not cytosolic bacteria [56,57]. We observed a modest but not a statistically significant increase in the number of cytosolic bacteria at 7 hr p.i (peak time point of cytosolic replication [57]) in Vps41 siRNA treated cells (S4h Fig: control siRNA: 28±3% and Vps41 siRNA: ~36±4%), suggesting that majority of bacteria (~70%) continue to harbor their vacuolar niche upon HOPS depletion. In concordance with these studies, immunogold-EM of ultrathin sections of *Salmonella*-infected Vps41 depleted cells at 10 hr p.i. showed presence of several vacuolar bacteria surrounded by limiting membrane positive for late endosomal and lysosomal markers-Rab7 and LAMP1 (Fig 4j–4m).

### Depletion of HOPS subunits prevents SIF formation and blocks SCV interaction with late endosomes and lysosomes

*Salmonella* survival and replication inside its vacuole strictly correlates with its ability to form SIFs, which begins at 5–6 hr p.i,. and is best visualized at 8–10 hr p.i. by immunostaining for lysosomal glycoproteins in *Salmonella*-infected cells [10]. Notably, as compared to the control cells, we did not observe SIF formation at later time points of infection (6 hr and 10 hr p.i.) in cells depleted of either of the six HOPS subunits (S6a–S6i Fig). In contrast, SIF formation was observed in TGFBRAP1-depleted cells (S6j Fig), however SIFs were “beaded” and thinner in these cells, which might explain the modest defect in *Salmonella* replication as shown in Fig 2b. To establish whether formation or stability of SIFs was reduced upon HOPS depletion, we performed live-cell imaging to visualize GFP-LAMP1 (marker for SIFs) dynamics in control-, Vps39- and Vps41-siRNA treated cells that were infected with DsRed-expressing *Salmonella*. At 9 hr p.i., time-lapse imaging revealed extensive SIF formation in control cells that was completely absent in Vps41- and Vps39-depleted cells (S4–S6 Movies). Moreover, as compared to the control cells, significantly fewer LAMP1-positive vesicles were found to interact with SCVs in Vps41- and Vps39-depleted cells (S5 and S6 Movies).

**Fig 5:**
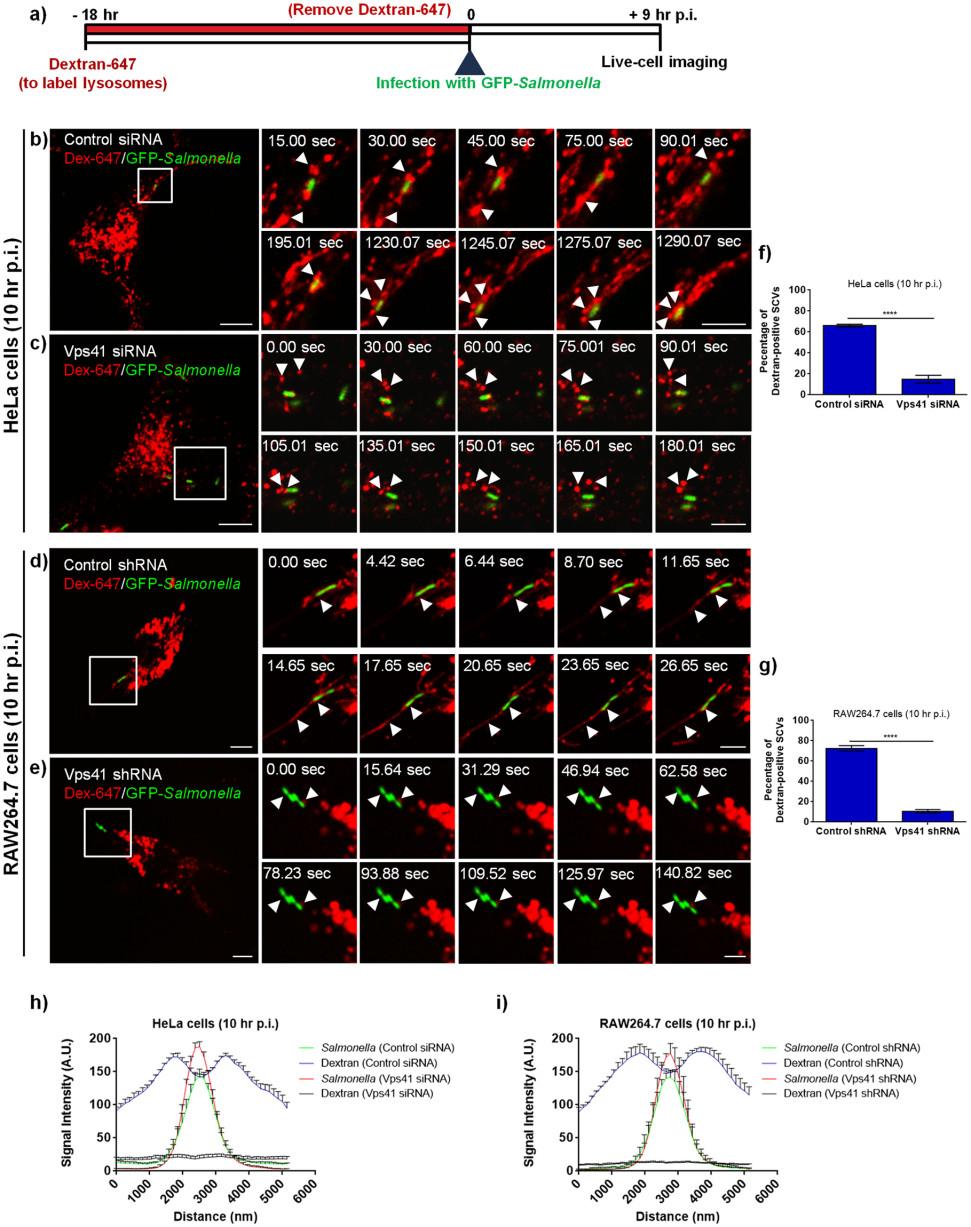
Interaction of dextran-loaded lysosomes with SCVs is impaired upon Vps41 depletion. **a)** Schematic illustrating the protocol used for loading of lysosomes with Alexa-Fluor 647-conjugated dextran in HeLa and RAW264.7 cells, prior to infection with GFP-expressing *Salmonella*. **b-e)** HeLa cells treated with control siRNA (**b**) or Vps41 siRNA (**c**) or RAW264.7 cells transduced with control shRNA (**d**) or Vps41 shRNA (**e**) were pre-incubated with Alexa-Fluor 647-conjugated dextran (red) to label lysosomes, followed by infection with GFP-expressing *Salmonella* (green). Time-lapse series for Alexa-Fluor 647-conjugated dextran loaded and infected cells were recorded at 10 hr p.i., and still images from representative time lapse series are shown (S7–S10 Movies). Different panels represent a higher magnification of the boxed area and the white arrowheads indicate the SCVs. Bars: (main) 10 μm; (insets) 5 μm. **f and g)** Quantification of Alexa-Fluor 647-conjugated dextran-positive SCVs in control and Vps41 depleted HeLa and RAW264.7 cells fixed at 10 hr p.i. Data represent mean ± S.D. over three independent experiments where ~100 SCVs were counted in each experiment (****, P < 0.0001; Student’s *t* test). **h and i)** Quantification of Alexa-Fluor 647-conjugated dextran signal intensity around the SCVs in control and Vps41 depleted HeLa and RAW264.7 cells. Data represent mean ± S.E.M. of signal intensity from three independent experiments at 10 hr p.i. where ~50 SCVs were counted in each experiment.

**Fig 6:**
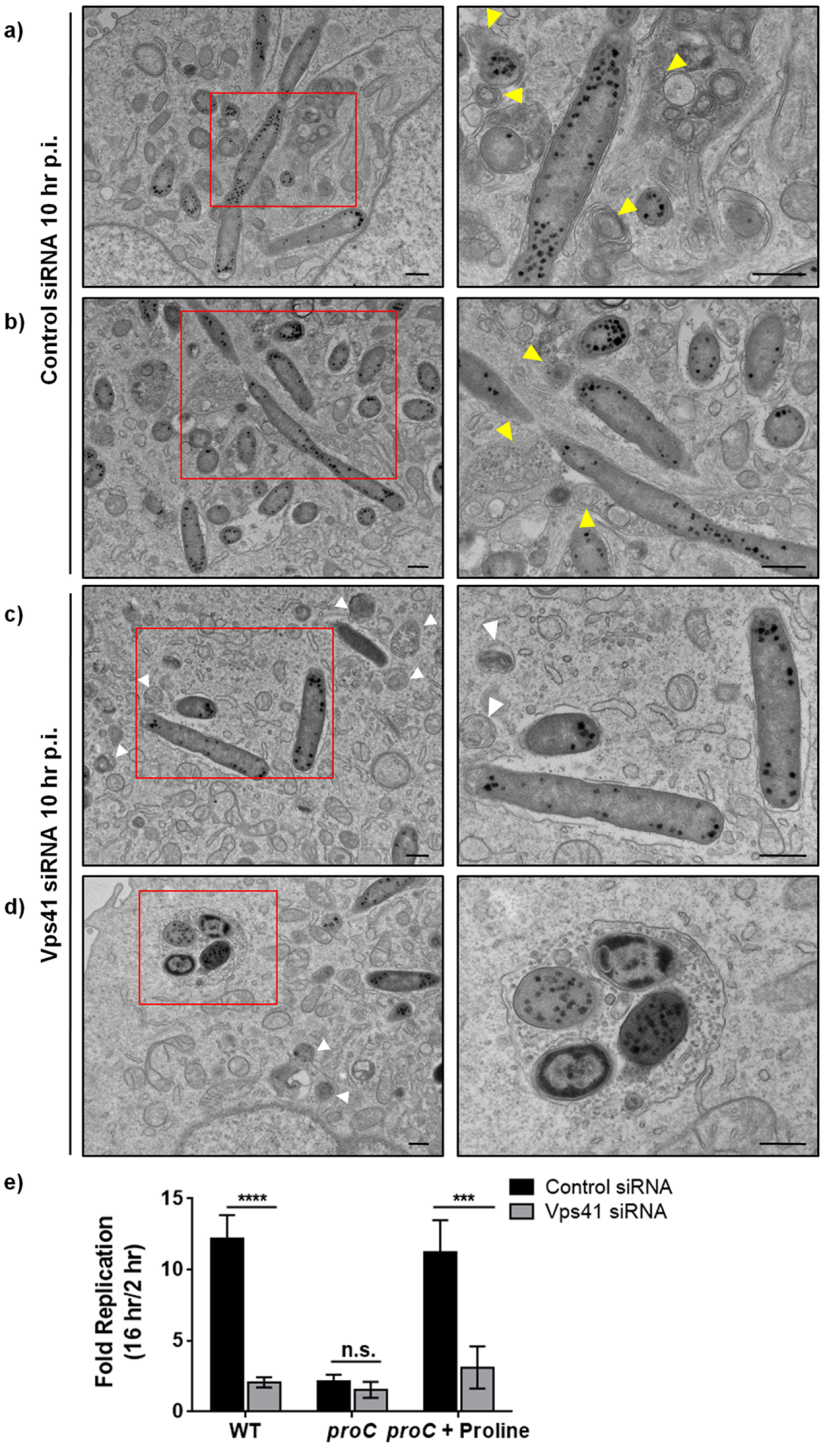
Silencing of Vps41 abrogates docking of late endosomes and lysosomes at SCVs and impairs nutrient acquisition by auxotrophic strain of *Salmonella*. **a-d)** Representative TEM images of control (**a** and **b**) and Vps41 (**c** and **d**) siRNA treated HeLa cells infected with *Salmonella* for 10 hr. Higher magnification of multiple SCVs (marked by yellow arrowheads) interacting with late endosomes (containing MVBs) and lysosomes (containing lamellar membrane sheets) in control siRNA treated cells are shown in the panels on the right. In Vps41 siRNA treated cells, white arrowheads depict the MVBs containing late endosomal compartments. Bar: 500 nm. **e**) Intracellular replication of wild-type (WT) *Salmonella* and a mutant strain auxotrophic for proline (*proC*) was determined at indicated times p.i. in control siRNA- or Vps41 siRNA-transfected HeLa cells. The fold change in intracellular proliferation was calculated as the ratio of CFU at 16 hr p.i./CFU at 2 hr p.i. To complement the intracellular proliferation of *proC Salmonella* strain, cell culture medium was supplemented with proline (0.8 mM). Shown are the means ± S.D. from three independent experiments (n.s., not significant; ***, P < 0.001; ****, P < 0.0001; Student’s *t* test).

Previous studies have shown that SCV association with the late endocytic compartments is significantly increased by 6–8 hr p.i., time points that correlate with the onset of SIF formation [10]. However, whether SIF formation is dependent upon SCV fusion with late endosomes/lysosomes and the host machinery that regulates this fusion is not known. Our results demonstrating that HOPS complex localizes to SCV and SIFs, suggest that similar to its role in mediating late endosome-lysosome fusion, this tethering factor could facilitate SCV fusion with lysosomes. To test this, prior to infection we pre-loaded control siRNA- and Vps41 siRNA-treated HeLa cells or control and Vps41 shRNA stably transduced RAW264.7 macrophages with Alexa 647-conjugated dextran (dextran-647) that specifically labels lysosomes, as shown schematically in Fig 5a. Live-cell imaging performed at 10 hr p.i. in control HeLa and RAW264.7 cells showed several dextran-positive endosomes undergoing fusion with the SCVs, resulting in acquisition of dextran by the SCVs (Fig 5b and 5d; S7 and S9 Movies). SIF formation was also observed in both control siRNA/shRNA-treated cells (S7 and S9 Movies). In contrast, little or no interaction of SCVs with the dextran compartment was observed in Vps41 depleted HeLa and RAW264.7 cells (Fig 5c and 5e; S8 and S10 Movies). Quantification of SCVs positive for dextran-647 and its signal intensity, revealed significantly lower dextran acquisition in Vps41 depleted cells compared to control (Fig 5f–5i; percentage of dextran-positive SCVs in HeLa and RAW264.7 cells-control: ~65–70%, Vps41 depletion: 10–15%). These results suggest that HOPS complex is required for acquisition of fluid-phase content by the SCVs from late endosomes and lysosomes. In agreement with these findings, imaging of ultrathin sections of *Salmonella*-infected control cells by TEM demonstrated several late endosomes (containing numerous MVBs) and lysosomes (containing lamellar membrane sheets) docked at or in close apposition to the SCVs (Fig 6a and 6b; S7b and S7c Fig; see magnified insets). In contrast, late endosomes/lysosomes docking at the SCVs were highly reduced in Vps41 depleted cells (Fig 6c and 6d; S7a, S7d and S7e Fig; see magnified insets). Further, as previously noted in another study [23], we also observed several abnormal “bag-like” SCVs upon Vps41 depletion (Fig 6d; see magnified inset). Additionally, in few TEM sections, SIF formation was also observed in control but not Vps41 depleted cells (S7b Fig, *middle panel*). Analysis of several TEM images in control cells revealed that of the ~100 SCVs imaged, ~40 SCVs had closely apposed late endosomes, whereas only ~10 of the 100 SCVs in Vps41 siRNA treated cells and none of the ~60 SCVs imaged in Vps41 shRNA transduced cells showed docked late endosomes. As previously reported [58], we also noted that lysosomes (containing lamellar membrane sheets) were reduced in Vps41 siRNA treated cells while several large MVB-containing compartments were observed (Fig 6c and 6d; indicated by white arrowheads). Although docking of late endocytic compartments at the SCVs was reduced upon Vps41 depletion, this did not indicate a general defect in the formation of late endocytic compartments. This was confirmed by LysoTracker Red uptake in control and Vps41 depleted cells, which is a selective probe that labels acidic organelles and routinely used as a specific marker to label late endosomes and endolysosomes. Immunofluorescence analysis and quantification of LysoTracker Red signal intensity by flow cytometry revealed no significant difference in control and Vps41 depleted cells (S8a–S8e Fig). The specificity of this probe was confirmed by treating cells with Baf A1 that neutralizes the pH of late endocytic compartments, and hence the signal intensity was reduced to background fluorescence levels (S8e Fig). We also confirmed that functional endo-lysosomes are formed upon Vps41 depletion by comparing levels of mature cathepsin B and D in control and Vps41 siRNA treated cells (S8f Fig). Taken together, our findings suggest that HOPS complex is a crucial host factor required for SCV fusion with the late endocytic compartments that provide membranes for formation of a replicative vacuolar niche for this pathogen.

### Depletion of HOPS subunits blocks SCV access to extracellular source of nutrition

Recent studies have shown that content mixing of SCV with the late endocytic compartments and SIF formation not only provides membrane for vacuolar integrity for the growing bacterial population but also provides nutrient access to the vacuolar bacteria for replication [14,15,17]. This was in part established by use of auxotrophic strains of *Salmonella* that were deficient in biosynthesis of particular amino acids. The mutant strains were able to replicate by obtaining nutrients from the growth medium of the host cells, only if they were proficient in SIF formation [15]. Based on our findings that HOPS complex mediates SIF formation by promoting SCV interaction with the host late endocytic compartments, we investigated role of HOPS subunits in mediating nutrient access from host cell to SCVs. To this end, we infected control- and Vps41-siRNA treated cells with proline auxotrophic strain of *Salmonella* (*proC*). This strain lacks the last enzyme required for proline biosynthesis, and is defective in intracellular replication unless proline is provided in the mammalian cell growth media [15]. As previously noted [15], we also found that *proC* strain was replication-defective as compared to the wild-type (WT) *Salmonella* strain. This growth defect was completely augmented by addition of proline in the culture media of control siRNA-treated HeLa cells (Fig 6e). In contrast, upon depletion of HOPS subunit Vps41, only a modest increase in the replication of *proC* strain in presence of extracellular proline was observed, which was significantly less than the control cells under the same experimental conditions (Fig 6e). These results suggest that HOPS complex provides nutrient access from the host late endosomes and lysosomes to the bacteria within the confinements of the vacuole, enabling intravacuolar replication of *Salmonella*.

### *Salmonella* effector SifA in complex with the host protein SKIP recruits HOPS subunits to SCVs and SIFs

**Fig 7:**
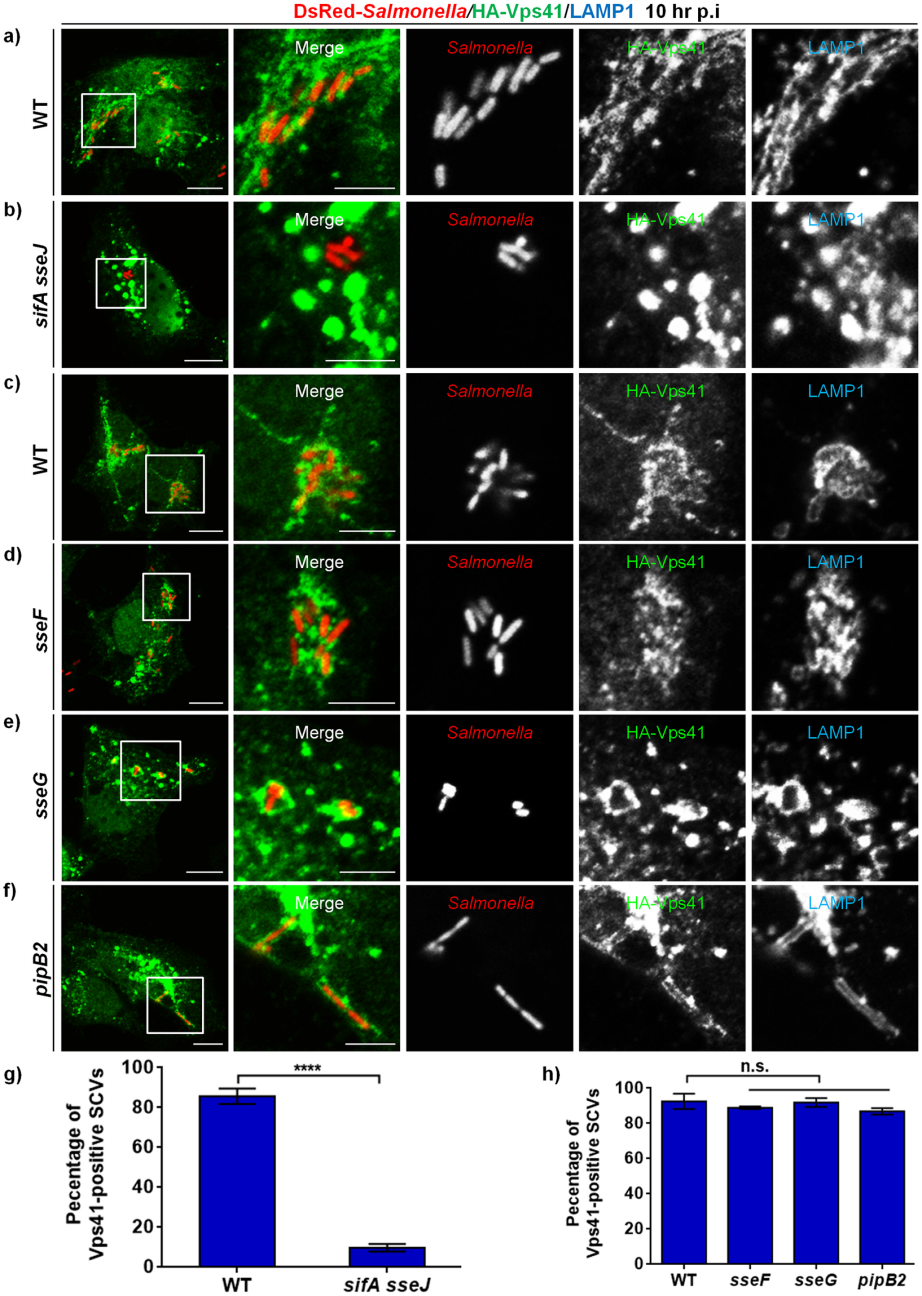
Deletion of *Salmonella* effector SifA abrogates Vps41 recruitment to SCV membranes. **a-f)** HeLa cells were infected with either DsRed expressing-wild-type (WT) strain of *Salmonella* (NCTC 12023 in (**a**) and SL1344 in (**c**)) or *sifA sseJ*, *sseF*, *sseG*, and *pipB2* strains, followed by transfection with HA tagged-Vps41. Cells were fixed at 10 hr p.i., and co-stained with anti-HA (green) and anti-LAMP1 (blue) antibodies. Different panels represent a higher magnification of the boxed areas. Bars: (main) 10 μm; (insets) 5 μm. **g and h)** Quantification of Vps41-positive SCVs in HeLa cells infected with different *Salmonella* strains (as labeled) and fixed at 10 hr p.i. Data represent mean ± S.D. from three independent experiments where ~100 SCVs were counted in each experiment (n.s., not significant; ****, P < 0.0001; Student’s *t* test).

Previous studies have revealed that *Salmonella* mutant strains deficient in SPI2-T3SS effectors *sifA*, *pipB2*, *sseF* and *sseG* show the most striking changes in SIF formation [12,16]. The most severe phenotype was observed in *Salmonella* strain lacking *sifA* where SIF formation was completely abrogated and vacuolar integrity was disrupted, leading to bacterial release in the host cytosol [18]. Our findings thus far indicate that HOPS complex is a crucial host factor required for SCV and SIF fusion with the late endocytic compartments, providing a continuous supply of membranes for SIF formation. To determine whether *Salmonella* effectors involved in SIF formation promote HOPS recruitment to SCV membranes, we visualized and quantified the recruitment of HOPS subunits Vps41 (both epitope-tagged and endogenous) and Vps18 (endogenous) to LAMP1-positive SCVs in mutant strains deficient in either *sifA ssej*, *pipB2*, *sseF* or *sseG* effectors (Fig 7 and S9 Fig). We used the *sifA sseJ* double-mutant strain in these experiments instead of the *sifA* single mutant strain as the latter loses its vacuolar integrity over time and becomes cytosolic [17]. Surprisingly, as compared to the WT strain of *Salmonella*, we did not observe recruitment of HOPS subunits-Vps41 and -Vps18 to *sifA ssej* SCVs, although association of these SCVs with the vacuolar membrane marker-LAMP1 was observed (Fig 7a and 7b; quantification shown in Fig 7g; S9a, S9b, S9g and S9h Fig). Notably, Vps41 and Vps18 continued to localize at the SCVs in cells infected with *Salmonella* mutant strains *pipB2*, *sseF* and *sseG* (Fig 7c–7f; quantification shown in Fig 7h; S9c–S9f and S9i–S9l Fig). These results suggest that SifA, but not other *Salmonella* effectors, involved in SIF formation are crucial for recruitment of HOPS subunits.

**Fig 8:**
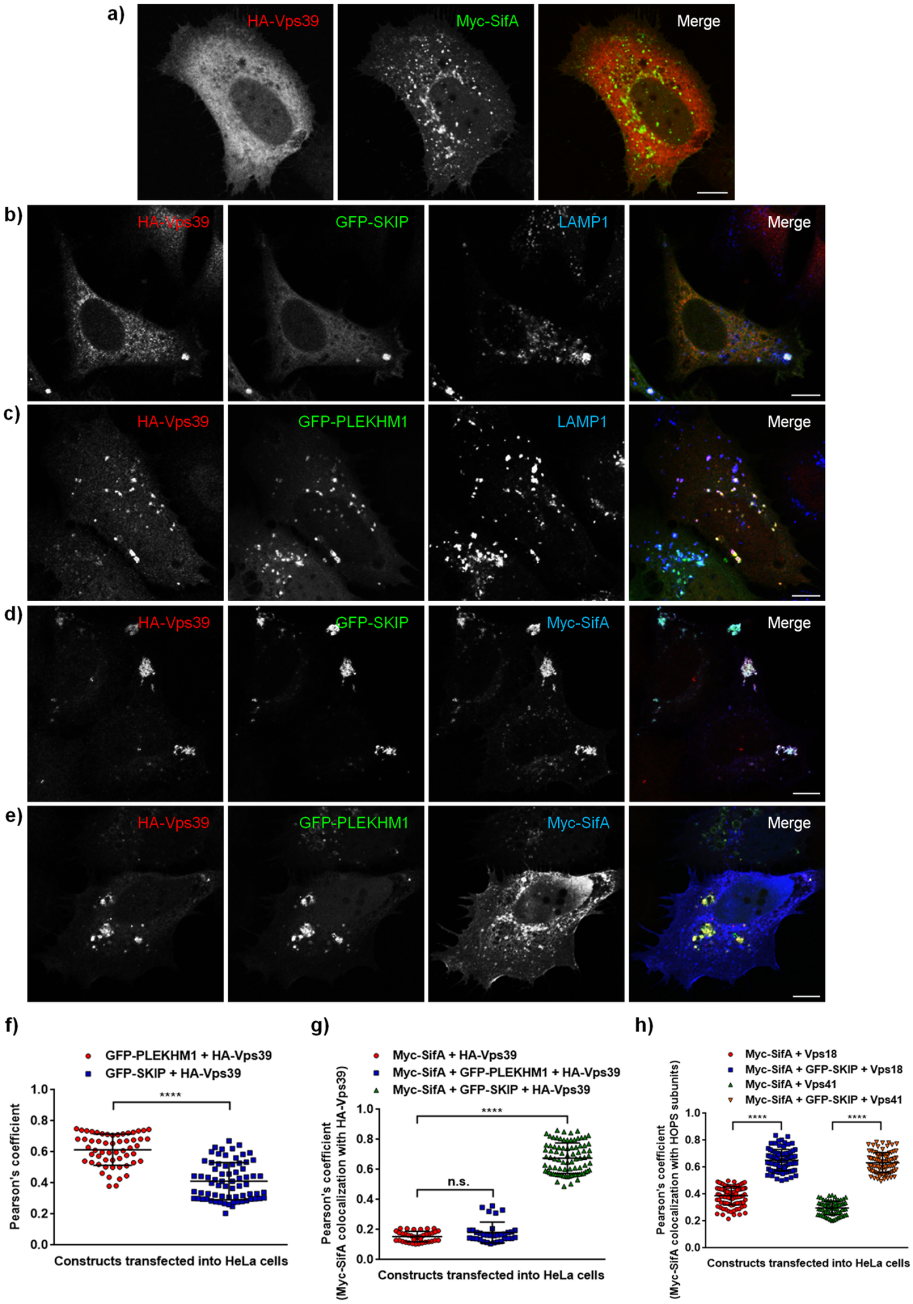
Bacterial effector SifA localizes with HOPS subunit Vps39 in a SKIP-dependent manner. **a-e)** Representative confocal micrographs of HeLa cells co-transfected with HA-Vps39 (red) and Myc-SifA (green) (**a**), HA-Vps39 (red) and GFP-SKIP (green) (**b**), HA-Vps39 (red) and GFP-PLEKHM1 (green) (**c**), HA-Vps39 (red), GFP-SKIP (green) and Myc-SifA (blue) (**d**), or HA-Vps39 (red), GFP-PLEKHM1 (green) and Myc-SifA (blue) (**e**). Cells in (**b**) and (**c**) were also stained for lysosomes using with anti-LAMP1 (blue) antibodies. Bars: 10 μm. **f-h)** Pearson’s correlation coefficient was calculated for the indicated protein pairs in transfected cells as labeled. Data represent mean ± S.D. over three independent experiments where ~25–30 transfected cells were analyzed in each experiment (n.s., not significant; ****, P < 0.0001; Student’s *t* test).

Intriguingly, we had previously found that SifA interaction partner-SKIP colocalizes and interacts with Vps39 subunit of the HOPS complex [27]. Based on these observations, we hypothesized that SifA in complex with SKIP targets HOPS complex to SCV membranes. Indeed, while little or no colocalization of Vps39 with SifA was observed, Vps39 colocalized with SKIP on peripheral structures shown to be lysosomes, which are transported in an anterograde manner by direct binding between Arl8b-SKIP complex to the plus-end microtubule binding motor-kinesin-1 (Fig 8a and 8b) [20,59,60]. Notably, colocalization between SifA and Vps39 was strikingly enhanced upon co-expression with SKIP and the three proteins were localized on the peripheral pool of lysosomes (compare Fig 8a and 8d). The other subunits-Vps18 and Vps41, also showed a significantly higher colocalization with SifA in presence of SKIP (compare S10a and S10c Fig; compare S10b and S10d Fig). Quantification of Pearson’s Correlation Coefficient (PCC) from 25–30 transfected cells over three independent experiments demonstrated a significant increase in colocalization of HOPS subunits with SifA in presence of SKIP (Fig 8g and 8h).

Recently PLEKHM1, a protein with domain architecture similar to SKIP, was reported to interact with both SifA and HOPS subunits Vps39 and Vps41 [23,29]. While it was speculated that PLEKHM1 acts as a linker between SifA and HOPS complex, no experimental evidence was shown to prove the same. Indeed, we found a strong colocalization of Vps39 with PLEKHM1, which was significantly higher than its colocalization with SKIP (Fig 8b and 8c; quantification of PCC shown in Fig 8f). To determine whether PLEKHM1, similar to SKIP, promotes colocalization of HOPS subunits with SifA, we co-expressed Vps39 and SifA with PLEKHM1. Surprisingly, while Vps39 and PLEKHM1 continued to colocalize on punctate structures, SifA was not recruited to these punctae (Fig 8e; quantification of PCC shown in Fig 8g). These results indicate that PLEKHM1 does not promote HOPS subunit association with SifA. We also noted that colocalization and interaction of SifA with PLEKHM1 was significantly weaker than with SKIP, as revealed by colocalization coefficient quantification and growth curve analysis of yeast two-hybrid assay using SifA as a bait, and SKIP and PLEKHM1 as prey proteins (S10e–S10h Fig). These findings were corroborated by GST pulldown assay where pull down of PLEKHM1 with GST tagged-SifA was found to be much lower as compared to SKIP from transfected cell lysates (S10i and S10j Fig). Additionally, qRT-PCR analysis revealed that SKIP mRNA levels in HeLa cells were ~2.5 fold higher than PLEKHM1 levels (S10k Fig). Taken together, these results imply that at least in this cell line, more amount of the secreted bacterial effector SifA must be bound to SKIP as compared to PLEKHM1.

**Fig 9:**
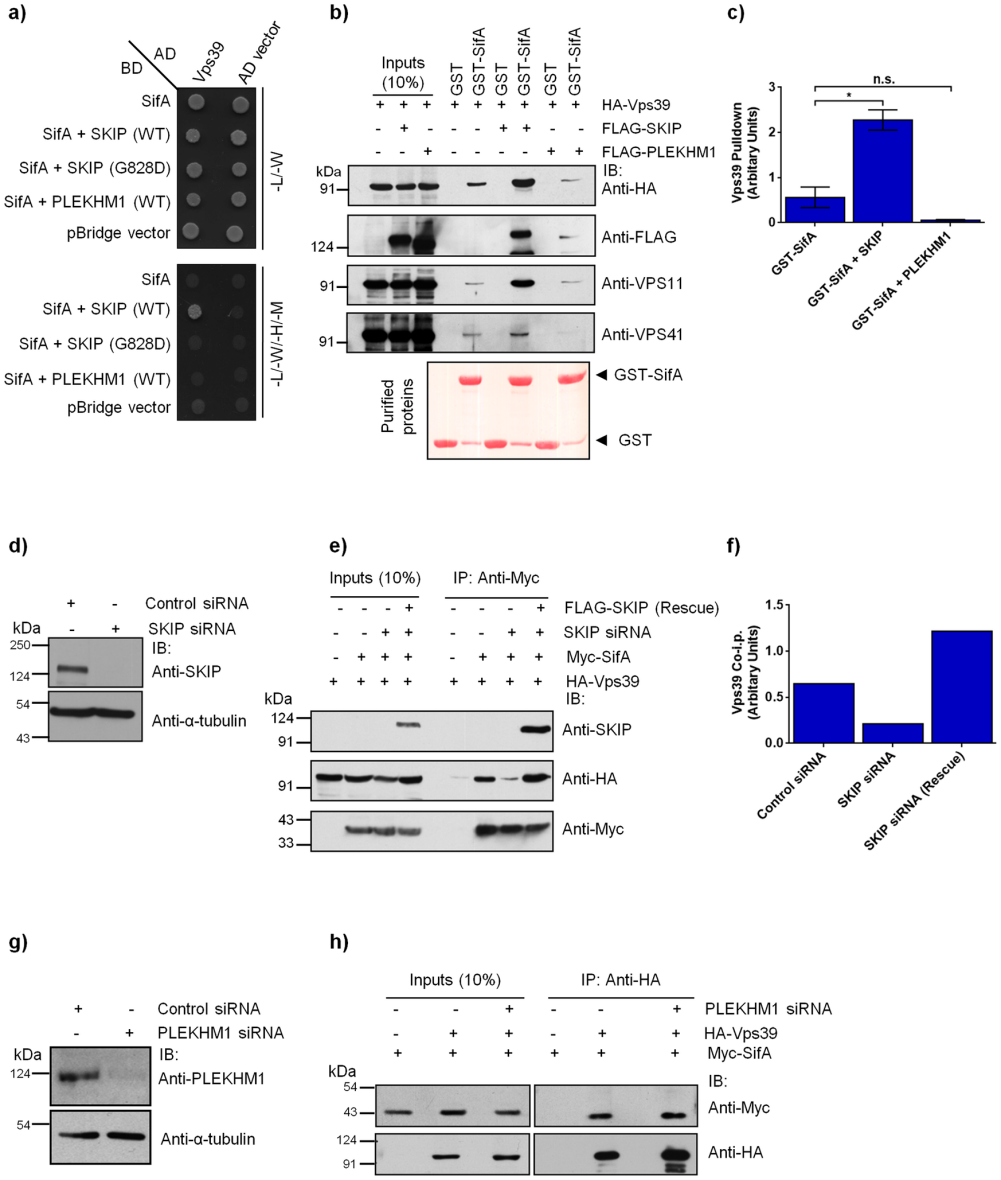
SKIP is required for interaction between bacterial effector SifA and HOPS complex. **a)** Yeast three-hybrid assay. Co-transformants were spotted on -Leu-Trp medium to check for viability, and on -Leu-Trp-His-Met media to test the interaction between SifA and Vps39 in the presence of SKIP (WT), SKIP (G828D) mutant or PLEKHM1. **b)** GST or GST tagged-SifA were immobilized on resin and incubated with lysates prepared from HEK293T cells transfected with HA-Vps39 alone or co-transfected with HA-Vps39 and FLAG-SKIP or FLAG-PLEKHM1. The precipitates were resolved by SDS-PAGE and immunoblotted with indicated antibodies. Ponceau S stain was done to visualize purified protein. **c)** Densitometric analysis of immunoblots of HA-Vps39 pulldown (normalized to input signal band intensity) by GST tagged-SifA in presence of FLAG-SKIP or FLAG-PLEKHM1. **d)** Control siRNA- or SKIP siRNA-treated HeLa cell lysates were resolved by SDS-PAGE, and immunoblotted with anti-SKIP antibody for assessing the knockdown efficiency and with anti-α-tubulin antibody as the loading control. **e)** Lysates from HEK293T cells treated with control- or SKIP-siRNA and transfected with indicated plasmids were immunoprecipitated using anti-Myc antibodies-conjugated resin. The cell lysates (inputs) and immunoprecipitates were resolved by SDS-PAGE and immunoblotted by Western blotting with the indicated antibodies. **f**) Densitometric analysis of immunoblots of HA-Vps39 co-immunoprecipitated (normalized to input signal band intensity) with Myc-SifA in control siRNA-, SKIP siRNA- or SKIP siRNA rescue construct-transfected HEK293T cell lysates. **g)** Lysates from control siRNA- or PLEKHM1 siRNA-treated HeLa cells were resolved by SDS-PAGE, and immunoblotted with anti-PLEKHM1 antibody for assessing the knockdown efficiency and with anti-α-tubulin antibody as the loading control. **h)** Lysates from HEK293T cells treated with control- or PLEKHM1-siRNA and expressing HA-Vps39 and Myc-SifA were immunoprecipitated with anti-HA antibodies-conjugated resin and precipitates were resolved on SDS-PAGE and immunoblotted with indicated antibodies.

To conclusively determine whether SKIP is a linker between SifA and HOPS subunit-Vps39, we employed yeast three-hybrid assay to test interaction of SifA and Vps39 in the presence of either SKIP or PLEKHM1 as well as a SifA binding-defective mutant of SKIP (SKIP G828D). In this assay, linker protein is under the control of the Met25 promoter that remains repressed in the presence of methionine in the growth media. As depicted in Fig 9a, under methionine-deficient conditions, SifA showed interaction with Vps39 only in the presence of SKIP, but not SKIP G828D mutant or PLEKHM1. To corroborate these results, we also performed GST pulldown using GST tagged-SifA as bait to pulldown Vps39 in cells with endogenous or overexpressed levels of SKIP. We observed a dramatic increase in the levels of Vps39 pulldown with SifA upon SKIP overexpression (Fig 9b and 9c). This striking increase in pulldown of HOPS subunits was also reflected upon probing for endogenous Vps11, which directly binds to Vps39 during assembly of the HOPS complex (Fig 9b). Vps41 pulldown with SifA was also increased upon SKIP overexpression, although this was less striking as compared to Vps39 and Vps11 (Fig 9b). To establish that endogenous levels of SKIP are sufficient to drive this interaction, we performed co-IP of SifA and Vps39 in control and SKIP depleted cells (Fig 9d; >90% gene silencing efficiency observed). As shown in Fig 9e and 9f, co-IP of Vps39 with SifA was significantly reduced upon SKIP depletion, and was restored upon expression of the siRNA-resistant SKIP construct, suggesting that SKIP acts as a linker to facilitate interaction between SifA and HOPS complex. In line with these observations and in accordance with previous studies [20,61], we found a significant defect in bacterial replication in SKIP-depleted cells as compared to control (S10l Fig; control siRNA: ~3 fold and SKIP siRNA: ~1.3 fold increase in bacterial burden from 2 hr to 10 hr p.i.). Notably, we did not observe any increase in pulldown of HOPS subunit Vps39 with GST tagged-SifA upon PLEKHM1 overexpression (Fig 9b and 9c). Similarly, no effect on the levels of co-IP Myc-tagged SifA with HA-Vps39 was observed upon PLEKHM1 depletion (Fig 9g; >90% silencing efficiency observed; Fig 9h), suggesting that PLEKHM1 does not facilitate interaction between SifA and HOPS complex.

**Fig 10:**
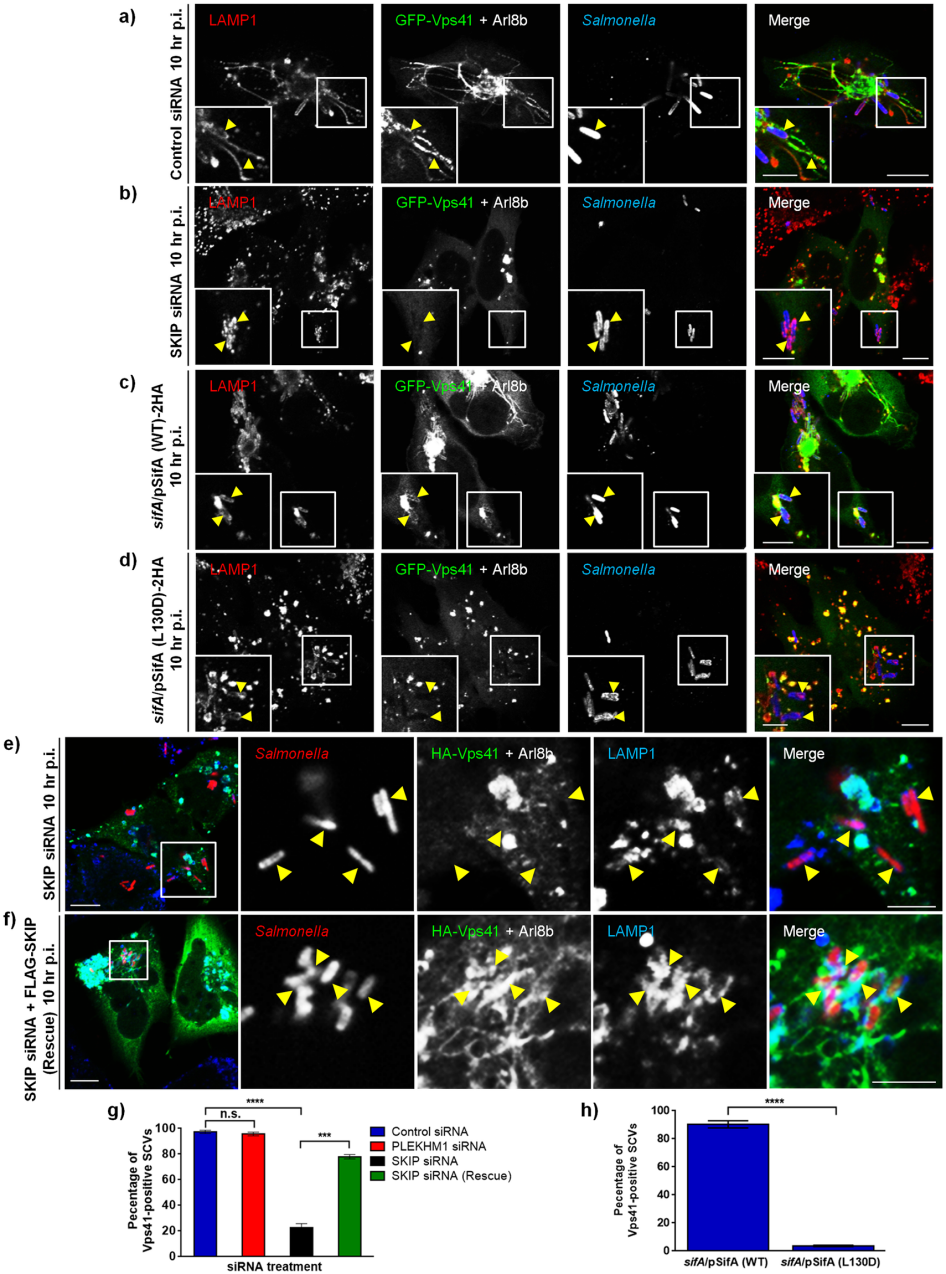
Recruitment of HOPS subunit Vps41 to SCVs requires SifA-SKIP interaction. **a and b)** Representative confocal micrographs of control siRNA- or SKIP siRNA-treated HeLa cells infected with *Salmonella*, followed by co-transtection with plasmids expressing GFP-Vps41 (green) and untagged-Arl8b. Cells were fixed at 10 hr p.i., and immunostained with antibodies to *Salmonella* (blue) and LAMP1 (red). Arrowheads in the insets depict localization of Vps41 around SCV membranes (marked by LAMP1). **c and d)** Representative confocal micrographs of HeLa cells infected with *Salmonella* strains *sifA/*pSifA (WT)-2HA (**c**) or *sifA/*pSifA (L130D)-2HA (**d**), followed by co-transfection with plasmids expressing GFP-Vps41 (green) and untagged-Arl8b. Cells were fixed 10 hr p.i., and immunostained with antibodies to *Salmonella* (blue) and LAMP1 (red). Arrowheads in the insets depict localization of Vps41 around SCV membranes (marked by LAMP1). **e and f)** Representative confocal micrographs of HeLa cells treated with SKIP siRNA, infected with DsRed-expressing *Salmonella*, followed by transfection with plasmids expressing HA-Vps41, untagged-Arl8b and vector (**e**) or siRNA-resistant FLAG-SKIP (rescue construct) (**f**). Cells were fixed 10 hr p.i., and immunostained with antibodies to HA tag (green) and LAMP1 (blue). Different panels represent a higher magnification of the boxed areas, indicating Vps41 localization around SCV membranes (marked by arrowheads). Bars: (main) 10 μm; (insets) 5 μm. **g)** Quantification of HA-Vps41-postive SCVs at 10 hr p.i. in indicated siRNA treated HeLa cells. Data represent mean ± S.D. of ~50 SCVs scored in each experiment for three independent experiments (n.s., not significant; ***, P < 0.001; ****, P < 0.0001; Student’s *t* test). **h)** Quantification of HA-Vps41-positive SCVs at 10 hr p.i. in HeLa cells infected with indicated *Salmonella* strains. Data represent mean ± S.D. of ~100 SCVs scored in each experiment for three independent experiments (****, P < 0.0001; Student’s *t* test).

To then determine whether SKIP is required for recruitment of HOPS subunits to SCV membranes, we visualized Vps41 localization to SCVs in control and SKIP siRNA treated cells. As shown in Fig 10a and 10b, while Vps41 was present around the SCVs in control siRNA treated cells, little or no association was observed in SKIP depleted cells at 10 hr p.i. Quantification of Vps41-positive SCVs in control and SKIP siRNA treated cells demonstrated that Vps41 recruitment to SCV membranes was abrogated upon SKIP depletion (Fig 10g). These findings were corroborated by live-cell imaging experiments of GFP-tagged Vps41 either expressed alone or co-expressed with Arl8b in control and SKIP siRNA treated cells (S11–S14 Movies; S11a and S11b Fig). Recruitment of Vps41 to SCVs was rescued by expression of siRNA-resistant SKIP, confirming specificity of SKIP siRNA treatment (Fig 10e–10g). In contrast, Vps41 continued to associate with SCV membranes in PLEKHM1 depleted cells at 10 hr p.i. (S11c and S11d Fig; Fig 10g), which supports our previous results that PLEKHM1 does not regulate SifA interaction with HOPS complex. To corroborate these findings, we used an independent method to disrupt interaction of SifA and SKIP i.e. infection with *Salmonella* strain expressing a point mutant of SifA (L130D), which is defective in binding to SKIP and formation of SIFs [21,61]. Using co-IP approaches, we confirmed that SKIP does not interact with the previously reported SKIP-binding interface mutants of SifA (S11e Fig) [21]. Also, unlike SifA deletion (*sifA*), bacteria expressing SifA (L130D) do not escape to the cytosol and continue to be surrounded by LAMP1-positive vacuolar membrane [61], which allowed us to analyze whether HOPS complex was recruited to the SCVs surrounded by an intact vacuolar membrane. Notably, as compared to the cells infected with the *sifA* strain expressing SifA (WT)-2xHA plasmid, in cells infected with *sifA* strain expressing point mutant SifA (L130D)-2xHA, little or no association of HOPS subunit-Vps41 with SCVs was observed at 10 hr p.i. (Fig 10c and 10d). Quantification of Vps41-positive SCVs infected with either strain demonstrated that recruitment of HOPS subunit Vps41 to SCV membranes was abrogated in the presence of SKIP-binding defective mutant of SifA (Fig 10h). These findings were corroborated by live-cell imaging experiments of GFP-tagged Vps41 either expressed alone or co-expressed with Arl8b in cells infected with either *Salmonella* strain (S15–S18 Movies; S11f and S11g Fig).

**Fig 11:**
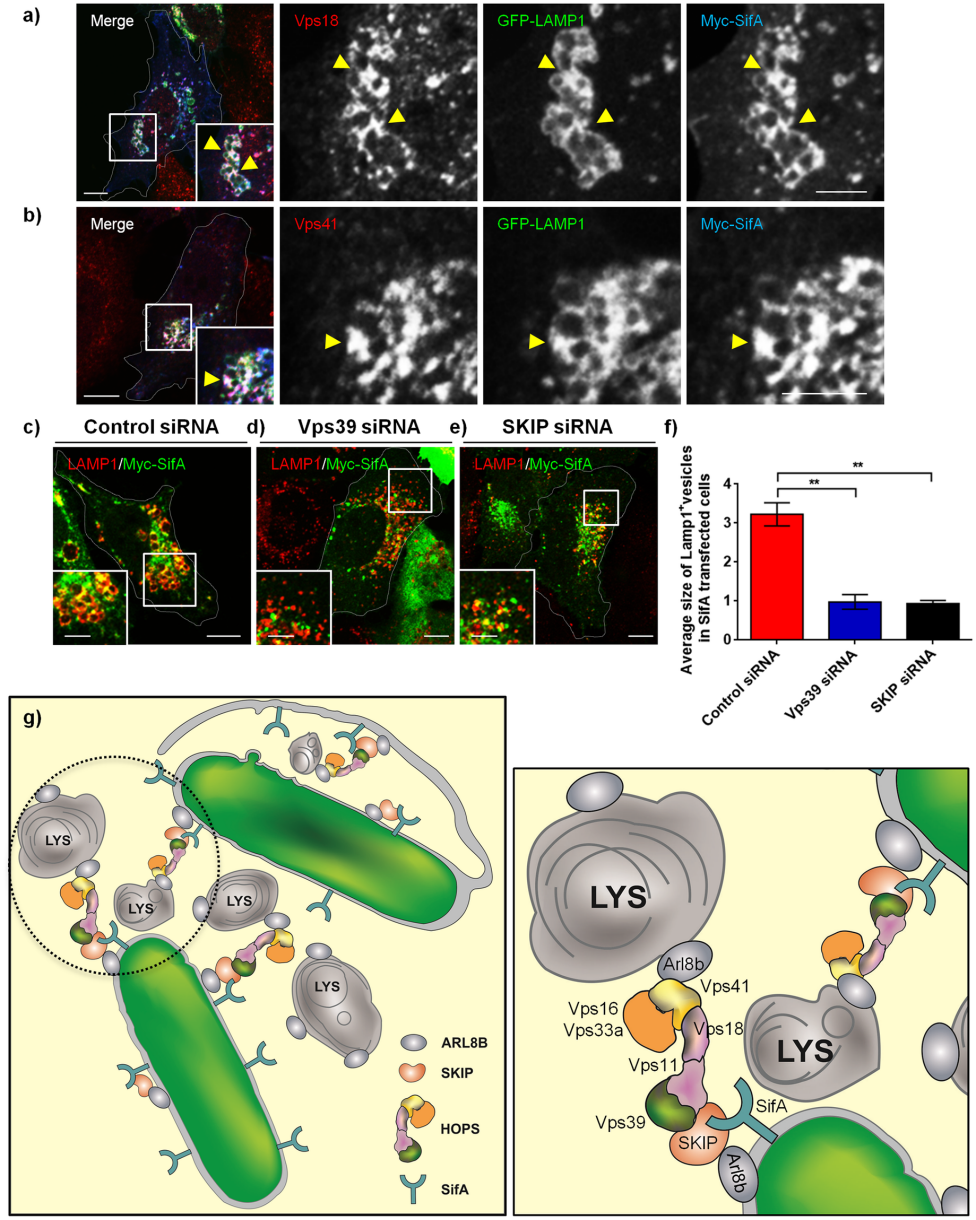
SifA-dependent lysosome clustering requires both SKIP and Vps39. **a-b)** Representative confocal micrographs of HeLa cells co-transfected with Myc-SifA and GFP-LAMP1 plasmids. The cells were fixed and co-stained using anti-Myc (blue) and anti-Vps18 (**a**, red) or anti-Vps41 (**b**, red) antibodies. Different panels represent a higher magnification of the boxed areas and HOPS subunits enrichment on clustered lysosomes is indicated by the arrowheads. **c-e**) HeLa cells treated with indicated siRNA were transfected with Myc-SifA expressing plasmid. The cells were fixed and co-stained using anti-Myc (green) and anti-LAMP1 (red) antibodies. Insets show higher magnification of the boxed areas clustered lysosomes induced by SifA expression, which is dependent upon Vps39 and SKIP expression. Bars: (main) 10 μm; (insets) 5 μm. **f)** Average size of LAMP1-positive compartments calculated in cells treated with indicated siRNA and transfected with Myc-SifA plasmid. Data represent mean ± S.D. of ~25–30 transfected per experiment over three independent experiments (**, P < 0.01; Student’s *t* test). **g)** Schematic depicting the molecular machinery required for SCV fusion with late endosomes and lysosomes. Multisubunit tethering factor-HOPS complex is a target for *Salmonella* effector SifA, which associates with its known binding partner-SKIP to recruit HOPS complex to SCV membranes, thereby enabling SCV fusion with Arl8b-positive lysosomes.

A previous study has shown that SifA protein expression in host cells results in the extensive clustering/aggregation of specifically the late endocytic compartments marked by LAMP1 and V-ATPase immunostaining [62]. Taking our results presented here into consideration, SifA could promote SCV fusion with late endosomes/lysosomes by virtue of its interaction with the host factors, SKIP and HOPS complex. Indeed, endogenous HOPS subunits-Vps18 and -Vps41, were enriched on the vertices of these clustered LAMP1-positive compartments induced by ectopic expression of SifA (Fig 11a and 11b). To test whether SifA-mediate clustering and aggregation of late endosomes and lysosomes requires presence of SKIP and HOPS subunits, we transfected SifA in control-, Vps39- and SKIP-siRNA treated cells and analyzed particle size of LAMP1-positive compartment. Our results show that SifA-mediated increase in lysosomal particle size depends upon the expression of SKIP and HOPS subunit-Vps39 (Fig 11c–11f). Taken together, our findings indicate that *Salmonella* virulence factor SifA in complex with the host protein, SKIP, recruits the vesicle fusion machinery of the host including the tethering factor HOPS complex to SCV membranes, thereby, enabling SCV fusion with late endosomes and lysosomes.

## Discussion

*Salmonella typhimurium* is a successful intracellular pathogen that has developed an array of sophisticated strategies to massively remodel the host endosomal system for its own survival and propagation. Previous studies have shown that SCV biogenesis involves extensive interactions with the host endocytic pathway including late endosomes/lysosomes [2]. However, little is known about how *Salmonella* mediates these interactions and whether it co-opts the late endosomal-lysosomal vesicle fusion machinery of the host cell for building its replicative niche. Conflicting reports have shown that while *Salmonella* inhibits activation of the small GTPase Rab7 [24,63], it actively recruits Arl8b on SCV and SIFs [25] wherein both Rab7 and Arl8b are components of protein machinery required for late endosome-lysosome fusion [26]. Intriguingly, Arl8b-positive lysosomes are less acidic and have reduced proteolytic activity than Rab7-positive endosomes [64]. It is interesting to speculate that Arl8b- but not Rab7-positive lysosomes act as source of membrane for SCV biogenesis and SIF formation during later time points of infection. This would ensure membrane and cargo delivery to SCVs without increasing the proteolytic activity within *Salmonella*’s replicative niche.

In this study, we have investigated the role of HOPS complex, a multisubunit tethering factor required for vesicle fusion with lysosomes, in regulating *Salmonella* survival and replication inside its vacuole. Our results reveal that HOPS complex is a target for *Salmonella* effector SifA, which in collaboration with its known binding partner SKIP and the host GTPase, Arl8b, recruits HOPS complex to SCV membranes, thereby enabling SCV fusion with lysosomes (Fig 11g). As late endocytic compartments are a source for both membrane and fluid-phase cargo, including nutrients for *Salmonella* residing in the vacuole [2,14], silencing of HOPS subunits inhibited *Salmonella* replication under both *in vitro* and *in vivo* conditions.

Unlike the defense strategies used by intracellular pathogens such as *M*. *tuberculosis* and *C*. *burnetti* [65,66], *Salmonella* does not block the maturation of its phagosome, which rapidly (~30–60 min p.i.) acquires several (but not all) characteristics of the late endocytic compartments but does not become bactericidal [2]. The acidic pH of the SCV (~<5) is required for the induction of the SPI-2 effectors, which in turn facilitate *Salmonella* replication inside the host cell [67]. At 1–2 hr p.i., we found weak but consistent localization of HOPS subunits on mature SCVs, which correlated with the recruitment of the lysosomal marker, LAMP1. While HOPS complex localized to the mature SCVs, we did not find an essential role of HOPS subunits, Vps41 and Vps39, in SCV maturation as indicated by a modest delay but not a block in LAMP1 acquisition in HOPS depleted cells. Our results support previous studies suggesting that SCV maturation is akin to an early to late endosome maturation event, regulated by proteins including PI(3) kinase and Rab7 (acquired upon HOPS depletion, Fig 4) that act upstream of the HOPS complex in endo-lysosome fusion [24,51].

Previous live-cell imaging studies of *Salmonella*-infected HeLa cells and RAW264.7 macrophages have shown that at 6–8 hr p.i., 90% of SCVs interact with dextran-loaded terminal lysosomes, and acquire not only membrane but also fluid-phase cargo from these compartments [9,12]. Besides delivering membranes for SCV biogenesis, fusion with late endosomes/lysosomes provides access to nutrients for bacterial replication [14,15]. Intravacuolar *Salmonella* can access nutrients from the host endolysosomal compartments by direct fusion of SCV with these membranes or from cytosol by recruitment of nutrient transporters on SCV and SIF membranes. In both of these scenarios, extensive membrane network will be required, which is delivered by host vesicle fusion machinery including HOPS complex. Accordingly, the ability of proline auxotrophic *Salmonella* strain to acquire proline from the extracellular media was also abrogated in HOPS-depleted cells. Besides their role as a tethering factor, HOPS subunits bind to SNARE proteins, which mediate membrane fusion [32,35]. We found a comparable defect in *Salmonella* intracellular replication when we depleted other components of the vesicle fusion machinery including small GTPases-Rab7 and -Arl8b, as well as SNARE proteins: Vti1b, Syntaxin 8, Syntaxin 17 and VAMP7 that are known to regulate late endosome-lysosome fusion [68]. These results indicate that *Salmonella* co-opts the host vesicle fusion machinery for survival and replication within its intravacuolar niche.

One of the hallmarks of *Salmonella* intracellular lifestyle is presence of striking tubular membranes or SIFs that emanate from the juxtanuclear SCVs [11]. The ability to form SIFs was found to directly correlate with *Salmonella*’s ability to replicate both under *in vitro* and *in vivo* conditions, as supported by the replication defect observed in *Salmonella* strains defective in SIF formation [19]. Recent studies have now shown that SIF formation allows *Salmonella* to convert the host cell endosomal system into a continuum with the SCV, not only providing SCVs access to the endocytosed material but the extensive SIF network is proposed to rapidly dilute the antimicrobial activities transferred to the vacuole upon its fusion with the host late endosomes and lysosomes. As a result, SCVs competent to form SIFs have bacteria with significantly higher metabolic activity than one that cannot form SIFs [14]. Using live-cell imaging we found that depletion of HOPS subunits completely inhibited SIF formation by *Salmonella*, supporting the strong replication defect observed in these cells.

SifA is the most well characterized *Salmonella* effector named for its essential role in mediating SIF formation [11]. Accordingly, *Salmonella* strains lacking SifA show a strong replication defect, as they fail to induce SIF formation and escape into the cytosol [19]. SifA has been shown to interact with two host proteins namely SKIP/PLEKHM2 and PLEKHM1 via pleckstrin homology (PH) domains of these proteins [20,23]. We found that SKIP, but not PLEKHM1, acts as a linker to mediate interaction of HOPS complex with SifA by simultaneously binding to HOPS subunit-Vps39. These results were surprising given the fact that previously PLEKHM1 was implicated in recruitment of HOPS complex to mediate SCV fusion with detoxified lysosomes [23]. However, the role of PLEKHM1 as a linker was never directly tested in this study and it was speculated based on the fact that PLEKHM1 binds to both HOPS complex and SifA [23,29]. A direct comparison of PLEKHM1 and SKIP’s linker role and their relative binding affinities for SifA as well as comparison of expression levels of both proteins in HeLa cells led us to conclude that SifA-SKIP promotes recruitment of HOPS subunits to SCV compartment. It will be interesting to determine whether SifA and Vps39 have overlapping binding sites on PLEKHM1, preventing SifA recruitment to PLEKHM1 and Vps39-positive compartment. Our study also suggests a novel role for SKIP in promoting *Salmonella* intracellular replication, besides its known function in preventing kinesin-1 accumulation on SCVs and regulating vacuolar integrity [20,22,59].

HOPS complex localization to SCVs and SIFs also required small GTPase Arl8b, which is highly enriched on these compartments and regulates lysosomal localization of both of its effectors-SKIP and Vps41 subunit of the HOPS complex [25,27,60]. Recently, we have uncovered that PLEKHM1, like SKIP, binds to Arl8b via its RUN domain and is a shared effector of Rab7 and Arl8b, which simultaneously binds to both GTPases to promote cargo trafficking to lysosomes [26]. Since *Salmonella* has devised a strategy to inhibit Rab7 activation, on the other hand Arl8b is enriched on SCVs and SIFs, it will be relevant to determine whether PLEKHM1 role in SCV fusion with lysosomes is dependent upon its interaction with Arl8b.

Unlike *Salmonella typhimurium*, much less is known about the intracellular lifestyle of the human-restricted pathogen-*Salmonella typhi*, the typhoid-causing strain of the same serovar. Intracellular *S*. *typhi* secretes the typhoid toxin inside its SCV, which is then packaged into vesicular carriers that are then transported into the extracellular space to mediate its effect in an autocrine and paracrine manner on the host cells [69,70]. Interaction of *S*. *typhi* vacuole with the host endocytic machinery and mechanisms regulating formation and transport of the typhoid toxin-containing vesicular carriers are only beginning to be understood [71,72]. Indeed, like *S*. *typhimurium*, intracellular replication of *S*. *typhi* was impaired in Rab7-depleted cells, suggesting that *S*. *typhi* might also manipulate host late endosomes and lysosomes to regulate biogenesis of its SCV and growth inside the host cells [73]. Future studies are required to address whether the host endocytic machinery regulates *S*. *typhi* replication and biogenesis of the typhoid toxin vesicular carriers that will reveal novel targets for development of antimicrobial molecules.

## Methods

### Cell culture

HeLa, HEK293T, and RAW264.7 cells were obtained from the American Type Culture Collection and maintained in DMEM (Lonza) supplemented with 10% heat-inactivated Fetal Bovine Serum (FBS; Life technologies) at 37°C in 5% CO_2_ humidified incubator. All the cultures were used between passage numbers 5–15.

An Arl8b-KO HeLa cell line was previously described [26]. Arl8b-knockout (KO) HeLa cells were generated using the Arl8b sg/RNA (Target sequence: 5′-GATGGAGCTGACGCTCG-3′) CRISPR/Cas9 All-in-One Lentivector Set (Applied Biological Materials). For stably silencing the expression of Vps41 in RAW264.7 cells, lentivirus mediated shRNA gene silencing approach was used. Briefly, for lentiviral transduction, RAW264.7 cells were seeded in a 35-mm tissue culture dish (Corning) in Polybrene (8 μg/ml; Sigma-Aldrich) and mixed with 500 μl of viral supernatant (day 0). Puromycin (Sigma-Aldrich) was added after 24–48 hr at 5 μg/ml for a minimum of 3 days to select transductants, and experiments were performed on days 5–15 after transduction. shRNA target sequences were as follows: Mission (negative control sequence), CAACAAGATGAAGAGCACCAA and mouse Vps41, GAGTGGCCTGGAGATCTATAT. Development of HeLa-Vps41 shRNA cell line was previously described using Vps41 shRNA, 5′-CCATTGACAAACCACCATTTA-3′ [27].

Primary mouse embryonic fibroblast (MEF) cells were isolated from the embryos of BALB/c mouse. Briefly, embryos were harvested from female mice 15 days after the appearance of the copulation plug. Embryos were placed in 1 ml of 0.05% trypsin/EDTA solution (Life technologies) and finely minced using a sterile razor blade and repeated pipetting was performed to dissociate cells. The trypsin was inactivated by adding DMEM supplemented with 10% FBS and the culture was centrifuged to pellet MEF cells. The pelleted MEF cells were resuspended in culture media, and plated at optimal density in tissue culture dishes at 37°C in 5% CO_2_ humidified incubator.

### Antibodies and chemicals

The following antibodies were used in this study: mouse anti-FLAG M2 clone (F1804; Sigma-Aldrich), mouse anti-HA (MMS-101P; Covance), rabbit anti-HA (sc-805; Santa Cruz Biotechnology), rat anti-HA clone 3F10 (11867423001; Roche), mouse anti-Myc 9E10 clone (sc-40; Santa Cruz Biotechnology), mouse anti-α-tubulin (T9026; Sigma-Aldrich), mouse anti-GAPDH (sc-166574; Santa Cruz Biotechnology), mouse anti-EEA1 (610457; BD Biosciences), rabbit anti-EEA1 (ab2900; Abcam), mouse anti-LAMP1 (555798; BD Biosciences), rabbit anti-LAMP1 (ab24170; Abcam), rabbit anti-PLEKHM1 (ab171383; Abcam), rabbit anti-SKIP/PLEKHM2 (HPA032304; Sigma-Aldrich), mouse anti-TGFBRAP1 (sc-13134; Santa Cruz Biotechnology), mouse anti-LBPA (Z-PLBPA; Echelon Biosciences), rabbit anti-Catalase (12980; Cell Signaling Technology), rabbit anti-Rab5 (3547; Cell Signaling Technology), rabbit anti-Rab7 (9367; Cell Signaling Technology), rabbit anti-Cathepsin D (K50161R; Meridian Life Sciences), mouse anti-Cathepsin B clone 4B11 (414800; Thermo Fisher Scientific), rabbit anti-*Salmonella* O-antigen (225341; BD Biosciences), and mouse anti-DnaK (ADI-SPA-880-F; Enzo Life Sciences). Rabbit anti-PLEKHM1 antibody generated against the N-terminal 497 amino acids of human PLEKHM1 protein was a gift from Prof. Paul Odgren (University of Massachusetts Medical School, Worcester, MA) and has been previously used to detect PLEKHM1 by immunofluorescence and Western blotting [26,74]. Rabbit anti-Arl8 antibody used in this study has been described previously [28].

For detection of HOPS subunits, the following antibodies were used: rabbit anti-Vps11 (ab125083; Abcam), rabbit anti-Vps18 (ab178416; Abcam), rabbit anti-Vps33a (16896-1-AP; ProteinTech), rabbit anti-Vps41 (ab181078; Abcam), and mouse anti-Vps41 (sc-377271; Santa Cruz Biotechnology).

All the Alexa fluorophore-conjugated secondary antibodies were purchased from Molecular Probes (Thermo Fisher Scientific). HRP-conjugated goat anti-mouse and goat anti-rabbit were purchased from Jackson ImmunoResearch Laboratories. Alexa Fluor 647-conjugated Dextran, LysoTracker Red DND-99 and DAPI were purchased from Molecular Probes (Thermo Fisher Scientific). L-Proline, Cytochalasin D, Bafilomycin A1, Polybrene, Streptomycin, Gentamicin and Puromycin were purchased from Sigma-Aldrich. Yeast drop-out media were purchased from Clontech.

### Bacterial strains, plasmids and infections

All the *Salmonella typhimurium* strains and plasmids used in this study are described in Table 1. For infection of HeLa and MEF cells, late-log *S*. *typhimurium* cultures were used and prepared using a method optimized for bacterial invasion [5]. Briefly, wild-type and mutant bacteria were grown for 16 hr at 37°C with shaking and then subcultured (1:33) in LB (Difco) without antibiotics and grown until late exponential phase (O.D. = 3.0). Bacterial inocula were prepared by pelleting at 10,000 x g for 2 min, diluted 1:100 in Phosphate buffer saline (PBS) (pH 7.2), and added to cells (at the specified MOI) for 10 min at 37°C to allow invasion and synchronized infection. After infection, extracellular bacteria were removed by extensive washing using warm PBS and 50 μg/ml gentamicin was added to the medium at 30 min p.i. for incubation at 37°C. After 2 hr p.i., the concentration of gentamicin in the medium was decreased to 5 μg/ml. Following this infection protocol, cells were processed for microscopy and biochemical experiments as described in the individual figure legends.

**Table 1:** Descriptions of the strains of S. typhimurium and the plasmids used in this study.

*Strains or Plasmids*	*Description*	*Source*
***Salmonella typhimurium strains***:		
*S*. *typhimurium* SL1344	Wild-type (WT); Streptomycin 50 μg/ml	Kind gift from Dr. John Brumell and described previously [62].
GFP-*S*. *typhimurium* SL1344	*S*. *typhimurium* SL1344 transformed with GFP expressing plasmid pFU95; Streptomycin 50 μg/ml Ampicillin 50 μg/ml	This Study
DsRed-*S*. *typhimurium* SL1344	*S*. *typhimurium* SL1344 transformed with DsRed expressing plasmid pFU96; Streptomycin 50 μg/ml Ampicillin 50 μg/ml	This Study
*S*. *typhimurium* SL1344 Δ*sifA*	*S*. *typhimurium* SL1344 with chromosomal deletion of SifA gene; Streptomycin 50 μg/ml	Kind gift from Dr. John Brumell and described previously [62].
*S*. *typhimurium* SL1344 Δ*sifA/*psifA-2HA	*S*. *typhimurium* SL1344 with chromosomal deletion of SifA gene complemented with a plasmid encoding epitope tagged SifA; Chloramphenicol 34 μg/ml	Kind gift from Dr. John Brumell and described previously [62].
*S*. *typhimurium* SL1344 Δ*sifA/*psifA (L130D)-2HA	*S*. *typhimurium* SL1344 with chromosomal deletion of SifA gene complemented with a plasmid encoding epitope tagged SifA having point mutation at amino acid residue position 130 (L to D); Streptomycin 50 μg/ml Chloramphenicol 34 μg/ml	This study
*S*. *typhimurium* NCTC 12023	Wild-type (WT)	Kind gift from Dr. Michael Hensel and described previously [15].
DsRed-*S*. *typhimurium* NCTC 12023	*S*. *typhimurium* NCTC 12023 transformed with DsRed expressing plasmid pFU96; Ampicillin 50 μg/ml	This Study
*S*. *typhimurium* NCTC 12023 (Δ*sifA*::FRT Δ*sseJ*::FRT)	*S*. *typhimurium* NCTC 12023 with a chromosomal deletion of effectors SifA and SseJ	Kind gift from Dr. Michael Hensel and described previously [15].
*S*. *typhimurium* NCTC 12023 (Δ*proC*::FRT)	*S*. *typhimurium* NCTC 12023 with a chromosomal deletion of Pyrroline-5-carboxylate reductase gene	Kind gift from Dr. Michael Hensel and described previously [15].
*S*. *typhimurium* SL1344 (Δ*sseF*::aphT)	*S*. *typhimurium* SL1344 with a chromosomal deletion of effector SseF; Streptomycin 30 μg/ml Kanamycin 50 μg/ml	Kind gift from Dr. Michael Hensel.
*S*. *typhimurium* SL1344 Δ*sseF*/Pro_*sseA*_*sscBsseF*::HA	*S*. *typhimurium* SL1344 with chromosomal deletion of SseF gene complemented with a plasmid encoding HA-epitope tagged SseF; Streptomycin 30 μg/ml Kanamycin 50 μg/ml Carbenicillin 50 μg/ml	This study
*S*. *typhimurium* SL1344 (Δ*sseG*::aphT)	*S*. *typhimurium* SL1344 with a chromosomal deletion of effector SseG; Streptomycin 30 μg/ml Kanamycin 50 μg/ml	Kind gift from Dr. Michael Hensel.
*S*. *typhimurium* SL1344 (Δ*pipB2*::aphT)	*S*. *typhimurium* SL1344 with a chromosomal deletion of effector PipB2; Streptomycin 30 μg/ml Kanamycin 50 μg/ml	Kind gift from Dr. Michael Hensel.
***Yeast two-hybrid constructs***:		
pGADT7 vector	GAL4-activation domain encoding yeast two-hybrid vector	Clontech
pGADT7-Vps39	Human Vps39 cloned into the pGADT7 vector	Described previously [27].
pGADT7-PLEKHM1	Human PLEKHM1 cloned into the pGADT7 vector	Described previously [26].
pGADT7-SKIP	Human SKIP cloned into the pGADT7 vector	Described previously [27].
pGBKT7 vector	GAL4-DNA binding domain encoding yeast two-hybrid vector	Clontech
pGBKT7-SifA	SifA cloned into the pGBKT7 vector	This study
***Yeast three-hybrid constructs***:		
pBridge vector	Yeast three-hybrid vector	Clontech
pBridge-SifA	SifA (1–330 aa) cloned into the MCS-I of the pBridge vector	This study
pBridge-SifA/SKIP	SifA (1–330 aa) cloned into the MCS-I and full-length SKIP cloned into the MCS-II of the pBridge vector	This study
pBridge-SifA/SKIP (G828D)	SifA (1–330 aa) cloned into the MCS-I and SKIP with point mutation at amino acid position 828 changing G with D, cloned into the MCS-II of the pBridge vector	This study
pBridge-SifA/PLEKHM1	SifA (1–330 aa) cloned into the MCS-I and full-length PLEKHM1 cloned into the MCS-II of the pBridge vector	This study
***Mammalian expression constructs***:		
pcDNA3.1(-) vector	Mammalian expression vector	Invitrogen
pcDNA3.1(-)- FLAG- TGFBRAP1	N-terminal FLAG-tagged human TGFBRAP1 cloned into the pcDNA3.1(-) vector	This study
pcDNA3.1(-)- FLAG- PLEKHM1	N-terminal FLAG-tagged human PLEKHM1 cloned into the pcDNA3.1(-) vector	Described previously [26].
pEGFPC1-PLEKHM1	N-terminal GFP-tagged human PLEKHM1 cloned into the pEGFPC1 vector	Described previously [26].
pcDNA3.1(-)-FLAG-SKIP	N-terminal FLAG-tagged human SKIP cloned into the pcDNA3.1(-) vector	Described previously [26].
pcDNA3.1(-)-siRNA-resistant-FLAG-SKIP	N-terminal FLAG-tagged full-length rescue construct against SKIP-siRNA cloned into the pcDNA 3.1(-) vector	Described previously [27].
pEGFPC1-SKIP	N-terminal GFP-tagged human SKIP cloned into the pEGFPC1 vector	Described previously [26].
pcDNA3.1(+)-Arl8b untagged	Human Arl8b cloned into the pcDNA3.1(+) vector	Described previously [26].
pcDNA3.1(+)-Mouse Arl8b-GFP	Full-length mouse Arl8b with C-terminal GFP tag cloned into the pcDNA3.1(+) vector	Described previously [26].
pEGFPC1-Rab7	N-terminal GFP-tagged human Rab7 cloned into the pEGFPC1 vector	Kind gift from Dr. Vesa Olkkonen and described previously [75].
pEBB-HA-Rab7	Full-length human Rab7 with N-terminal HA tag cloned into the pEBB vector	Kind gift from Dr. Jason Kinchen and described previously [76].
pcDNA3.1(-)-HA-Vps41	N-terminal HA tagged human Vps41 cloned into the pcDNA3.1(-) vector	Described previously [27].
pEGFPC1-Vps41	N-terminal GFP-tagged human Vps41 cloned into the pEGFPC1 vector	This study
pcDNA3.1(-)-HA-Vps39	N-terminal HA tagged human Vps39 cloned into the pcDNA3.1(-) vector	Described previously [27].
pcDNA3.1(-)-Vps33a-HA	C-terminal HA tagged human Vps33a cloned into the pcDNA3.1(-) vector	Described previously [27].
GFP-LAMP1	GFP-tagged Lgp120 (rat lamp-1)	Kind gift from Dr. Steve Caplan and described previously [44].
pEGFPC1-SifA	SifA cloned into the pEGFPC1 vector	Kind gift from Dr. Samuel Miller and described previously [77].
Myc-SifA	SifA cloned into the pCMV-myc vector	Kind gift from Dr. Samuel Miller and described previously [21].
Myc-SifA (L130D)	Myc-SifA construct with point mutation at amino acid residue position 130 (L to D) of SifA	This Study
Myc-SifA (M131D)	Myc-SifA construct with point mutation at amino acid residue position 131 (M to D) of SifA	This Study
***Bacterial expression constructs***:		
pGEX-5X-1-SifA	SifA cloned in pGEX-5-X-1 vector	Kind gift from Dr. Kasturi Haldar and described previously [78].
pFU95	Ap^r^; *gapA-rbs-gfpmut3*.*1*; ColE1	Kind gift from Dr. Petra Dersch and described previously [79].
pFU96	Ap^r^; *gapA-rbs-dsred2*; ColE1	Kind gift from Dr. Petra Dersch and described previously [79].
pSifA-2HA	2 HA-tags SifA cloned into the pACYC184 vector	Kind gift from Dr. John Brumell and described previously [62].
pSifA (L130D)-2HA	psifA-2HA construct with a point mutation at amino acid residue 130 (L to D) of SifA	This Study
Pro_*sseA*_*sscBsseF*::HA	pWSK29 plasmid containing expression cassette for HA epitope-tagged SseF effector	Kind gift from Dr. Michael Hensel and described previously [80].

For infections of RAW264.7 cells, stationary-phase bacterial cultures incubated at 37°C with shaking were diluted (O.D. = 1) and opsonized in PBS supplemented with 20% FBS for 20 min at 37°C. After three washes in PBS, bacteria were resuspended in growth medium without antibiotics, and added to the cells (MOI of 50:1) for 20 min to facilitate phagocytosis. The remaining protocol was similar as in case of infection of HeLa cells.

### Transfections and RNAi

Cells grown on glass coverslips (VWR) were transfected with desired constructs using X-tremeGENE-HP DNA transfection reagent (Roche) for 16–18 hr. For gene silencing, siRNA duplexes for non-targeting siRNA pool, control siRNA (5′-TGGTTTACATGTCGACTAA-3′), human Arl8b (5′-AGGTAACGTCACAATAAAGAT-3′), human Rab7a (5′-CTAGATAGCTGGAGAGATG-3′) human Vps11 (5′-GAGGCTGAGCTGAGCCTCGTATT-3′), human Vps18 (5′-CTAGATAGCTGGAGAGATG-3′), human Vps33a (5′-CATTGCAGTGTTGCCTCGATATG-3′), human Vps39 (ON-TARGET plus SMART pool), mouse Vps39 (ON-TARGET plus SMART pool), human Vps41 (5′-CCATTGACAAACCACCATTTA-3′), mouse Vps41 (ON-TARGET plus SMART pool), human PLEKHM1 (5′-CCGGTCTCTGCAAGAGGTATTGT-3′), human SKIP (5′-CTTCTGAACTGGACCGATT-3′), human Vti1b (ON-TARGET plus SMART pool), human Stx8 (ON-TARGET plus SMART pool), human Stx17 (ON-TARGET plus SMART pool), human Vamp7 (ON-TARGET plus SMART pool) and human TGFBRAP1 (ON-TARGET plus SMART pool) were purchased from GE Healthcare (Dharmacon), and transfection was performed using Dharmafect 1 as per the manufacturer's instructions.

### Immunofluorescence staining, confocal microscopy and colocalization analysis

Cells were fixed in 4% p-formaldehyde (PFA) in PHEM buffer (60 mM PIPES, 10 mM EGTA, 25 mM HEPES, and 2 mM MgCl_2_, final pH 6.8) for 10 min at room temperature. Post fixation, cells were incubated with blocking solution (0.2% saponin + 5% FBS in PHEM buffer) at room temperature for 30 min, followed by three washes with 1X PBS. After this blocking step, cells were incubated with primary antibodies in staining solution (PHEM buffer + 0.2% saponin) for 1 hr at room temperature, washed thrice with 1X PBS, and further incubated for 30 min with Alexa fluorophore-conjugated secondary antibodies made in staining solution. Cells were washed thrice with 1X PBS and mounted in Fluoromount G (Southern Biotech). Single-plane confocal images were acquired using a 710 Confocal Laser Scanning Microscope (ZEISS) equipped with a Plan Apochromat 63×/1.4 NA oil immersion objective and high-resolution microscopy monochrome cooled camera AxioCamMRm Rev. 3 FireWire (D) (1.4 megapixels, pixel size 6.45 μm × 6.45 μm). For image acquisition, ZEN Pro 2011 (ZEISS) software was used. All images were captured to ensure that little or no pixel saturation is observed. The representative confocal images presented in figures were imported into Adobe Photoshop CS and formatted to 300 dpi resolution. The whole image adjustment of brightness was done using curves function.

For all the colocalization analysis, at least 30 cells for each treatment per experiment were used for three independent experiments. Pearson’s Correlation Coefficient (PCC) was determined using the JACoP plugin of ImageJ where the threshold was set using maximum entropy.

### Dextran-647 loading and live-cell imaging

In order to trace the endocytic route, HeLa cells were incubated with Alexa-Fluor 647-conjugated dextran (Molecular Probes) for 16–18 hr. The cells were washed once with 1X PBS and infected with GFP-expressing *Salmonella* (at an MOI 50:1) and further incubated in a dextran-free medium for the rest of the experiment. Live-cell imaging was initiated at indicated time-points.

For live-cell imaging experiments, cells were seeded on glass-bottom tissue culture treated cell imaging dish (Eppendorf) and infected with either DsRed- or GFP- expressing *Salmonella* strains (at an MOI 50:1) as described above. Post-infection, imaging dish was loaded into a sealed live-cell imaging chamber (37°C and 5% CO_2_) for imaging in DMEM. Time-lapse confocal images were acquired at specified time-points using an LSM 710 confocal microscope with a LCI Plan Neofluar objective 63×/1.3 multi-immersion correction and equipped with a high-resolution microscopy monochrome cooled camera AxioCamMRm Rev. 3 FireWire (D). Image acquisition and adjustments to brightness and contrast was performed by using ZEN Pro 2011 software.

### Transmission electron microscopy

Sample processing and TEM was performed at the Harvard Medical School EM Facility (Boston, USA). Briefly, control shRNA or Vps41 shRNA transduced HeLa cells were infected with *S*. *typhimurium* SL1344 for 10 hr. Post-infection, cells were fixed in routine fixative (2.5% glutaraldehyde/1.25% PFA in 0.1 M sodium cacodylate buffer, pH 7.4) for at least 1 hr at room temperature and washed in 0.1 M sodium cacodylate buffer (pH 7.4). The cells were then post fixed for 30 min in 1% osmium tetroxide/1.5% potassium ferrocyanide, washed in water three times, and incubated in 1% aqueous uranyl acetate for 30 min, followed by two washes in water and subsequent dehydration in grades of alcohol (5 min each: 50, 70, 95, 2× 100%). Cells were removed from the dish in propylene oxide, pelleted at 3000 rpm for 3 min, and infiltrated overnight in a 1:1 mixture of propylene oxide and TAAB Epon (Marivac Canada). The samples subsequently embedded in TAAB Epon and polymerized at 60°C for 48 hr. Ultrathin sections were cut on a Reichert Ultracut-S microtome, picked up onto copper grids stained with lead citrate, and examined in a JEOL 1200EX transmission electron microscope. Images were recorded with an AMT 2k charge-coupled device camera.

### Double immunogold EM labeling

Sample fixation for immunogold EM was carried out as described previously [26], and double immunogold labeling and imaging was performed at the Harvard Medical School EM Facility (Boston, USA). For preparation of cryosections, control siRNA- and Vps41 siRNA-treated HeLa cells were infected with *S*. *typhimurium* as described above. After 2 hr p.i., cells were transfected with HA-Rab7 expressing construct and 10 hr p.i. cells were fixed with 4% PFA + 0.1% glutaraldehyde (Glu) prepared in 0.1 M sodium phosphate buffer, pH 7.4. After 2 hr fixation at room temperature, the cell pellet was washed once with PBS and then placed in PBS containing 0.2 M glycine for 15 min to quench free aldehyde groups. Before freezing in liquid nitrogen, the cell pellets were cryoprotected by incubating in three drops of 2.3 M sucrose in PBS for 15 min. Frozen samples were sectioned at -120°C, and the sections were transferred to formvar/carbon-coated copper grids. Grids were floated on PBS until the immunogold labeling was performed.

The double immunogold labeling was performed at room temperature on a piece of parafilm. All the primary antibodies and Protein A immunogold were diluted in 1% Bovine Serum Albumin (BSA) in PBS. In brief, grids were floated on drops of 1% BSA for 10 min to block for unspecific labeling, transferred to 5 μl drops of rat anti-HA, and incubated for 30 min. The grids were then washed in four drops of PBS for a total of 15 min, transferred to 5 μl drops of rabbit anti-rat for 30 min, and washed again in four drops of PBS for 15 min, followed by 15 nm Protein A immunogold for 20 min (5 μl drops). After the 15 nm Protein A immunogold incubation, grids were washed in four drops of PBS, fixed for 2 min with 0.5% Glu followed by four drops of PBS containing 0.2 M glycine for 15 min to quench free aldehyde groups. The labeling process was repeated with rabbit anti-LAMP1 followed by 10 nm Protein A immunogold for 20 min in 5 μl drops. Finally, the grids were washed in four drops of PBS and six drops of double-distilled water. Contrasting/embedding of the labeled grids was performed on ice in 0.3% uranyl acetate in 2% methyl cellulose for 10 min. Grids were picked up with metal loops, and the excess liquids were removed by blotting with a filter paper and were examined in an electron microscope (1200EX; JEOL). Images were recorded with an AMT 2k CCD camera.

### Cell lysates, co-immunoprecipitation and immunoblotting

For lysates, cells were lysed in ice-cold lysis buffer (25 mM Tris-HCl pH 7.4, 150 mM NaCl, 1 mM EDTA, 1% Triton X-100 and protease inhibitor cocktail). For co-IP experiments, HEK293T cells treated with control or gene-specific siRNA and transfected with indicated plasmids were lysed in ice-cold TAP lysis buffer (20 mM Tris, pH 8.0, 150 mM NaCl, 0.5% NP-40, 1 mM MgCl_2_, 1 mM Na_3_VO_4_, 1 mM NaF, 1 mM PMSF, and protease inhibitor cocktail; Sigma-Aldrich). The lysates were incubated with indicated antibody-conjugated agarose beads at 4°C rotation for 3 hr, followed by four washes in TAP wash buffer (20 mM Tris, pH 8.0, 150 mM NaCl, 0.1% NP-40, 1 mM MgCl_2_, 1 mM Na_3_VO_4_, 1 mM NaF, and 1 mM PMSF). The samples were then loaded on SDS-PAGE for further analysis. Protein samples separated on SDS-PAGE were transferred onto polyvinylidene fluoride (PVDF) membranes (Bio-Rad Laboratories). Membranes were blocked overnight at 4°C in blocking solution (10% skim milk in 0.05% PBS-Tween 20). Indicated primary and secondary antibodies were prepared in 0.05% PBS-Tween 20. The membranes were washed for 10 min thrice with 0.05% PBS-Tween 20 or 0.3% PBS-Tween 20 after 1 hr incubation with primary antibody and 1 hr incubation with secondary antibody, respectively. The blots were developed using a chemiluminescence-based method (Pierce).

### Intracellular replication assay

To enumerate intracellular *Salmonella* growth, gentamicin protection assay was performed. Briefly, cells were infected with designated *Salmonella* strain for different time periods using the protocol described above. At the end of every time point p.i. cells were gently washed with PBS followed by lysis using PBS containing 0.1% Triton X-100 and 1% SDS for 5 min at room temperature. The resulting lysates were serially diluted and plated onto LB agar plates containing streptomycin.

### Chloroquine (CHQ) resistance assay

To assess cytosolic *Salmonella* replication in HeLa cells upon Vps41 depletion, CHQ resistance assay was performed as described previously [56]. Briefly, control siRNA- or Vps41 siRNA-treated HeLa cells were seeded in 24-well plates and infected with *S*. *typhimurium* as described above. To evaluate cytosolic replication of *Salmonella*, 1 hr prior to 7 hr p.i. time point, two wells were treated with CHQ (150 μM) and gentamicin (5 μg/ml) for 1 hr (CHQ-resistant bacteria) and another two wells were incubated with gentamicin (5 μg/ml) only (total bacteria). At the end of 7 hr p.i. time point, duplicate gentamicin treated (total CFU) and duplicate CHQ + gentamicin-treated cells (cytosolic CFU) were solubilized and serial dilutions were plated on LB agar for CFU enumeration.

### *Salmonella* survival assay in morpholino-treated mice

Six weeks old C57BL/6 male mice were obtained from the CSIR-Institute of Microbial Technology (IMTECH) animal house facility and injected intravenously (i.v.) with 12.5 mg/Kg (of body weight) of either control (CCTCTTACCTCAGTTACAATTTATA) or mouse VPS41-specific (CCATAGCGCAGCCTGAGAGTCAT) vivo-morpholinos (purchased from Gene Tools, LLC) for two consecutive days at an interval of 24 hr, followed by *Salmonella* infection the third day. For *Salmonella* infection, stationary phase culture of *S*. *typhimurium* strain SL1344 was diluted to a CFU of ~1.3X10^3^ in 100 μl of 1X PBS and injected i.v. The infectious dose was quantified by plating plating dilution series on LB agar plates containing streptomycin. Mice were sacrificed after 3 days and dilution series of spleen and liver lysates (prepared in 0.05% sodium deoxycholate in 1X PBS) were plated on LB agar plates containing streptomycin.

### Ethics statement

This study was carried out in strict accordance to the guidelines issued by the Committee for the Purpose of Supervision of Experiments on Animals (No. 55/1999/CPCSEA) under the Prevention of Cruelty to Animals Act 1960 and amendments introduced in 1982 by Ministry of Environment and Forest, Government of India. All protocols involving mice experiments were approved by the Institutional Animal Ethics Committee (IAEC) of Council of Scientific and Industrial Research-Institute of Microbial Technology (Approval no. IAEC/16/12 and IAEC/17/13).

### Enrichment of *Salmonella*-Containing Vacuoles (SCVs)

Roughly 50 million HeLa cells infected with *S*. *typhimurium* SL1344 strain were used for subcellular fractionation of SCVs. At 3 hr and 8 hr p.i., cells were washed thrice with ice-cold PBS and scrapped into a 15 ml centrifuge tube using a rubber cell scrapper. The cells were centrifuged at 1000 rpm for 7 min and the cell pellets were suspended in ice-cold homogenization buffer (250 mM sucrose, 20 mM HEPES (pH 7.2), 0.5 mM EGTA and 5 μg/ml Cytochalasin D) containing protease inhibitor cocktail (Sigma-Aldrich) and transferred to a Dounce Homogenizer with a tight fitting pestle on ice to break the cells. Approximately 30 strokes were applied until almost 90% of the cells were broken without breaking the nuclei. The intact cells and nuclei were pelleted in microcentrifuge tube at 400 x g for 3 min. The resulting supernatant was collected in a fresh microcentrifuge tube to yield the post nuclear supernatant (PNS). The PNS was brought to a final concentration of 39% sucrose and layered on to 2 ml 55% sucrose which was in turn layered onto 65% sucrose cushion in a 13.2 ml open top Beckman ultracentrifuge tube followed by addition of 2 ml 32.5% and 2 ml 10% sucrose solutions. All sucrose solutions (w/v) were prepared in 20 mM HEPES (pH 7.2) and 0.5 mM EGTA. The PNS layered on sucrose gradient was then subjected for ultracentrifugation in a swinging bucket rotor for 1 hr at 100000 x g at 4°C. The fractions of 1 ml each were collected from top to bottom. Pooled fractions 8–10 were adjusted very slowly to a final sucrose concentration of 11% with homogenization buffer without sucrose and layered on 15% Ficoll cushion (5% sucrose, 0.5 mM EGTA and 20 mM HEPES pH 7.2). The samples in open top Beckman ultracentrifuge tube were spun at 18000 x g for 30 min in a Beckman SW 41 Ti rotor at 4°C. The supernatant was discarded and pellet was resuspended in 11 ml homogenization buffer. The samples were spun again at 18000 x g for 20 min in a Beckman SW 41 Ti rotor at 4°C and the resulting pellet was labeled as “SCV” fraction. The pelleted SCV fractions were resuspended in 20 μl of 4X SDS-sample buffer, boiled for 10 min and analyzed by SDS-PAGE and immunoblotting.

### Enrichment of *Salmonella*-modified membranes (SMMs) and affinity immuno-precipitation

A previously published three-step approach, lysis of infected host cells followed by intracellular compartment enrichment and affinity-IP, was used to enrich and determine the presence of HOPS subunits in SMMs [45]. Briefly, 16 hr prior to infection, 5 million HeLa cells were seeded in a 10-cm tissue culture dish and four 10-cm dishes were used per IP. For infection, *S*. *typhimurium* SL1344 *sseF* harboring a low-copy expression vector with a C-terminal HA-tagged SseF and its cognate chaperone sscB (*sseF*/SseF-HA) was used, and cells infection for a period of 8 hr was carried out as described above. Post-infection, cells were washed thrice with ice-cold PBS and scrapped into a 15 ml centrifuge tube using a rubber cell scrapper, and centrifuged at 1000 x g for 7 min. The resulting cells pellet was suspended in ice-cold homogenization buffer (250 mM sucrose, 20 mM HEPES, 0.5 mM EGTA, pH 7.4), centrifuged at 1000 x g for 10 min, and resuspended in 1 ml of 4°C pre-cooled homogenization buffer containing protease inhibitor cocktail (Sigma-Aldrich). The cells were mechanically disrupted by adding 100 μl of 0.5 mm glass beads (Sigma-Aldrich) using a vortexer (three 1 min strokes) with 5 min of intermediate cooling on ice. The lysate was centrifuged at 100 x g for 10 min at 4°C, and the resulting pellet (labeled as “GEMN pellet”) was washed twice with ice-cold homogenization buffer with protease inhibitor mixture. The final GEMN pellet was resuspended in 500 μl of homogenization buffer supplemented with 1.5 mM MgCl_2_ and treated with DNase I (50 μg/ml) for 30 min at 37°C. The protein concentration in the GEMN protein fraction was determined using Bradford reagent (Bio-rad).

For affinity-IP, 500 μg of GEMN proteins adjusted to a final volume of 500 μl in solubilization buffer (1.5 mM MgCl_2_, 10 mM KCl, 0.1% NP-40) were added to 20 μl of pre-blocked (in 1% BSA made in PBS for 30 min) anti-HA antibody-conjugated agarose beads or anti-myc antibody-conjugated agarose beads (Sigma-Aldrich) as a control, and were allowed to mix on rotary shaker at 4°C for 4 hr. At the end of the incubation period, beads were washed five times with 0.1% NP-40 made in PBS to remove non-specific proteins. Finally, the beads were resuspended in 20 μl of 4X SDS-sample buffer, boiled for 10 min and analyzed by SDS-PAGE and immunoblotting.

### GST pulldown assay

For protein expression and purification, bacterial expression vectors encoding for GST or GST tagged-SifA were transformed into *E*. *coli* BL21 strain. Primary cultures of a transformed single colony were set up for 12 hr at 37°C in LB broth containing plasmid vector antibiotic. Secondary cultures were set up using 1% primary inoculums and subjected to incubation at 37°C to an absorbance of 0.6 at 600 nm and then protein production was induced using 0.5 mM isopropyl β-D-1-thiogalactopyranoside (IPTG) for 5 hr at 30°C. After the incubation period, bacterial cultures were centrifuged at 4,000 rpm for 15 min, washed once with 1X PBS, and resuspended in ice-cold buffer (20 mM HEPES and 150 mM NaCl, pH 7.4) containing protease inhibitor tablet (Roche) and 1 mM PMSF. Cell lysis was performed by sonication, followed by centrifugation at 12,000 rpm for 15 min at 4°C. The supernatants were incubated with glutathione resin (Gbiosciences) on rotation for 2 hr at 4°C to allow binding of GST or GST tagged-SifA, followed by 10 washes with wash buffer (20 mM HEPES, 300 mM NaCl, and 0.5% Triton X-100, pH 7.4). For use in the pulldown assays, protein-bound glutathione resins were blocked with 5% BSA in PBS for 2 hr at 4°C.

For pulldown assays, transfected HEK 293T cells were lysed in ice-cold TAP lysis buffer, and lysates were incubated with protein-bound glutathione resins at 4°C for 3 hr with rotation. Samples were washed four times with TAP wash buffer, and elution was performed by boiling the samples in 1X SDS-PAGE loading buffer and loaded onto SDS-PAGE for analysis.

### Yeast two-hybrid and yeast three-hybrid assay

For the yeast two-hybrid assay, plasmids encoding GAL4-activation domain (AD) and GAL4-DNA binding domain (BD) fusion encoding constructs were co-transformed in *S*. *cerevisiae* AH109 strain, streaked on SD plates lacking leucine and tryptophan (SD-L/-W) and allowed to grow at 30°C for 3 days. The co-transformants were replated on non-selective medium (SD-L/-W) and selective medium (SD-leucine/-tryptophan/-histidine; SD-L/-W/-H) to assess interaction.

For measuring yeast growth rate, primary cultures were seeded in SD-L/-W broth (Clontech) from single colonies of *S*. *cerevisiae* AH109 strain co-transformed with indicated plasmids, and grown overnight at 30°C to saturation. The resulting cultures were diluted to approximately 0.1 OD (at 600 nm) in SD-L/-W/-H broth (Clontech) and culture growth was monitored every 4 hr for 48 hr.

For performing the yeast three-hybrid assay, the *S*. *cerevisiae* Gold strain (Clontech) was made sensitive to methionine (Met) by streaking the yeast on an SD/-Met media at least two times before transforming with the desired plasmids. After co-transformation, yeast cells were replated on SD-L/-W (nonselective; selects only for the presence of plasmid) or SD-L/-W/-H/-M (selective; requires interaction of bait and prey proteins through the linker protein for growth).

### LysoTracker red-uptake assay

The acidotropic dye LysoTracker Red DND-99 (Thermo Fisher Scientific) was diluted in Opti-MEM without phenol red (Thermo Fisher Scientific). To control siRNA- or Vps41 siRNA-treated HeLa cells cultures 100 nM LysoTracker Red was added and uptake for 1 hr was performed. At the end of the internalization period, cells were washed and then resuspended in fresh pre-warmed medium, and red lysosomal fluorescence of 10,000 cells per sample was determined by flow cytometry (BD Accuri). FlowJo v10 software was used to analyze all of the data from flow cytometric experiments. For visualization of LysoTracker Red-uptake signal by confocal microscopy, at the end of the dye uptake cells were fixed in 4% PFA in PHEM buffer at room temperature for 10 min. Post-fixation, cells were washed and mounted on glass slides and analyzed.

### Statistical analyses

Statistical analyses were performed with Prism 6 software (GraphPad). Data are presented as mean ± standard deviation (S.D.) unless otherwise indicated. P-values were calculated using two-tailed unpaired Student’s *t* test, and differences were considered significant when P < 0.05. The sample sizes are specified in the figure legends for all of the quantitative data.

## Supporting information

S1 Fig Localization of HOPS subunits to SCVs and SIFs at different times post *Salmonella* infection. **a)** Representative confocal micrographs of HeLa cells infected with DsRed-expressing *Salmonella* (red). At 10 min p.i., cells were fixed and stained for endogenous Vps41 (green) and EEA1 (blue). Different panels represent a higher magnification of the boxed areas, showing absence of Vps41 but presence of EEA1 around SCVs (marked by arrowheads) at this time point of infection. **b-e)** Representative confocal micrographs of HeLa cells infected with DsRed-expressing *Salmonella* (red). At different times after infection, cells were fixed and stained for endogenous Vps18 (green) and LAMP1 (blue, shown only in inset). Insets depict recruitment of Vps18 on SCVs and SIFs as marked by arrowheads. **f-m)** Representative confocal micrographs of HA-Vps41 or Vps33a-HA transfected HeLa cells infected with DsRed-expressing *Salmonella* (red). At different times after infection, cells were fixed and stained using anti-HA (green) and anti-LAMP1 (blue, shown only in inset) antibodies. Insets depict recruitment of epitope-tagged HOPS subunits on SCVs and SIFs as marked by arrowheads. Bars: (main) 10 μm; (insets) 5 μm.
(TIF)

S2 Fig HOPS- but not CORVET-specific subunit is recruited to SCV, which is dependent upon expression of lysosomal small GTPase Arl8b. **a-e)** Representative confocal micrographs of FLAG-TGFBRAP1 transfected HeLa cells infected with DsRed-expressing *Salmonella* (red). At different times after infection (as indicated), cells were fixed and stained using anti-FLAG (green) and anti-EEA1 (**a**, blue) or anti-LAMP1 (**b-e**, blue, shown only in inset) antibodies. Arrowheads in inset from panel (**a**) depict colocalization of TGFBRAP1 with EEA1. **f-j)** Representative confocal micrographs of Arl8b-GFP transfected HeLa cells infected with DsRed-expressing *Salmonella* (red). At different times after infection (as indicated), cells were fixed and stained using anti-LAMP1 (blue, shown only in inset) antibody. Insets depict higher magnification of boxed areas. Bars: (main) 10 μm; (insets) 5 μm. **k and l)** Time-lapse microscopy of WT or CRISPR/Cas9 Arl8b KO HeLa cells transfected with plasmid encoding GFP-Vps41, and infected with *Salmonella* expressing DsRed (red). Time-lapse series were recorded at the indicated times p.i., and still images correspond to movies shown as S1 and S3 Movies. Bars: (main) 10 μm; (insets) 5 μm. **m)** WT- and CRISPR/Cas9 Arl8b KO-HeLa cell lysates were immunoblotted with anti-Arl8 antibody for assessing the knockdown efficiency and with anti-α-tubulin antibody as a loading control. **n)** Quantification of GFP-Vps41-positive SCVs in WT- and Arl8b KO-HeLa cells. Data represent mean ± S.D. over three independent experiments at 10 hr p.i. where 100 SCVs were counted in each experiment (****, P < 0.0001; Student’s *t* test).
(TIF)

S3 Fig HOPS subunit Vps41 is required for intracellular replication of *Salmonella* in different cell types. **a-p)** Western blotting or qRT-PCR analysis of different cell types transfected with indicated siRNA or shRNA was performed to measure the gene silencing efficiency. **q and r)** Intracellular replication assay. RAW264.7 (**q**) or primary MEF cells (**r**) treated with indicated shRNA or siRNA, and infected with *Salmonella* were harvested at indicated times p.i. The number of CFU per well were determined and shown as dot plot. Data represent mean ± S.D. (n.s., not significant; ****, P < 0.0001; Student’s *t* test).
(TIF)

S4 Fig LAMP1 acquisition around SCVs does not require fusion with lysosomes. **a-c)** Representative confocal micrographs of control siRNA-, Vps41 siRNA- or Vps39 siRNA-treated HeLa cells infected with DsRed-expressing *Salmonella* (red). At 10 min p.i., cells were fixed and stained for early endosomes marker, EEA1 (green) and LAMP1 (blue). Insets depict higher magnification of the boxed areas showing localization of different markers on the SCVs. Shown below the image is the intensity scan profile to visualize colocalization of *Salmonella* (red) with EEA1 (green) and LAMP1 (blue). **d and e)** HeLa cells pre-treated with either DMSO (vehicle control) or Bafilomycin A1 (Baf A1) (50 nM) overnight were infected with DsRed-expressing *Salmonella* (red). At 10 hr p.i., cells were fixed and immunostaining for LAMP1 (green) was performed. The nuclei were stained using DAPI (blue). Insets depict higher magnification of the boxed areas showing localization of different markers on the SCVs. Bars: (main) 10 μm; (insets) 5 μm. **f and g)** The intensity scan profile to visualize colocalization of *Salmonella* (red) with LAMP1 (blue) in DMSO or Baf A1 treated HeLa cells is shown. **h)** Chloroquine (CHQ) resistance assay was performed to quantify the percentage of cytosolic bacteria in total population upon Vps41 silencing. HeLa cells seeded in a 24-well plate were transfected with control- or Vps41-siRNA, and infected with *Salmonella*. After 6 hr p.i., two wells were incubated with CHQ and gentamicin (CHQ-resistant bacteria, cytosolic bacteria) and two wells were incubated with gentamicin only (total bacteria) for 1 hr. At the end of 7 hr p.i., cells were harvested and the number of CFU per well were determined and the percentage of cytosolic bacteria proliferation was calculated as the ratio of CFU obtained at 7 hr p.i. in CHQ + gentamicin treated wells/CFU obtained at 7 hr p.i. in gentamicin alone treated wells. Data represent mean ± S.D. from three independent experiments (n.s., not significant; Student’s *t* test).
(TIF)

S5 Fig LBPA is not acquired around the SCVs in control and HOPS depleted cells. **a-f)** Representative confocal micrographs of control siRNA-, Vps39 siRNA- or Vps41 siRNA-treated HeLa cells infected with DsRed-expressing *Salmonella* (red). At 1 hr (**a-c**) and 6 hr (**d-f**) p.i., cells were fixed and stained for LBPA (green) and LAMP1 (blue). Insets depict higher magnification of the boxed areas showing localization of different markers on the SCVs. Bars: (main) 10 μm; (insets) 5 μm.
(TIF)

S6 Fig Depletion of HOPS complex subunits leads to absence of SIF formation. **a-j)** Representative confocal micrographs of control siRNA (**a** and **d**)-, HOPS subunits specific siRNA (**b**, **c**, and **e-i**)- or TGFBRAP1 siRNA (**j**)-transfected HeLa cells and infected with *Salmonella*. At different times after infection (as indicated), cells were fixed and immunostained with antibodies to *Salmonella* (green) and LAMP1 (red). The nuclei were stained using DAPI (blue). Insets represent a higher magnification of the boxed areas with arrowheads depicting LAMP1 localization on individual SCVs. Bars: (main) 10 μm; (insets) 5 μm.
(TIF)

S7 Fig Silencing of Vps41 abrogates docking of late endosomes and lysosomes at SCVs. **a)** Lysates of control shRNA or Vps41 shRNA transduced HeLa cells were immunoblotted with anti-Vps41 antibody for assessing the knockdown efficiency and with anti-α-tubulin antibody as the loading control. **b-e)** Representative TEM images of control shRNA (**b** and **c**) and Vps41 shRNA (**d** and **e**) transduced HeLa cells infected with *Salmonella* for 10 hr. Higher magnification of multiple SCVs interacting with late endosomes and lysosomes in control shRNA transduced HeLa cells are shown (marked by arrowheads). Arrowheads indicate SIF formation in inset of panel (**b**). Bar: 500 nm.
(TIF)

S8 Fig Late endocytic compartments are acidic and functional in Vps41 depleted cells. **a-d)** Representative confocal micrographs of control- and Vps41-siRNA treated HeLa cells incubated with LysoTracker Red (100 nM) for 1 hr. Bar: 10 μm. **e)** Control siRNA- and Vps41 siRNA-transfected HeLa cells were incubated with DMSO (vehicle control) or with Baf A1 incubated for 1 hr. After 1 hr, LysoTracker Red (100 nM) uptake was performed for 1 hr. At the end of the internalization period, cells were washed and fluorescence was determined by flow cytometry. **f)** Lysates from control- and Vps41-siRNA treated HeLa cells were resolved by SDS-PAGE and immunoblotted with indicated antibodies by Western blotting.
(TIF)

S9 Fig Deletion of *Salmonella* effector SifA abrogates acquisition of HOPS complex on SCV membranes. **a-l)** HeLa cells were infected with DsRed expressing-wild-type (WT) strain of *Salmonella* (NCTC 12023 (**a** and **g**) and SL1344 (**c** and **i**) or *sifA sseJ* (**b** and **h**), *sseF* (**d** and **j**), *sseG* (**e** and **k**), and *pipB2* (**f** and **l**) strains. Cells were fixed at 10 hr p.i., and co-stained with anti-Vps41 (green, **a**-**f**) or anti-Vps18 (green, **g-l**) and anti-LAMP1 (blue, shown only in inset) antibodies. Different panels represent a higher magnification of the boxed areas. Bars: (main) 10 μm; (insets) 5 μm.
(TIF)

S10 Fig Characterization of SifA interaction with SKIP and PLEKHM1. **a-d)** Representative confocal micrographs of HeLa cells transfected with Myc-SifA alone (red), or co-transfected with Myc-SifA (blue) and GFP-SKIP (green). Cells were fixed and stained with antibodies against Vps41 (red, **a** and **c**) and Vps18 (red, **b** and **d**). Bars: 10 μm. **e and f)** Representative confocal micrographs of HeLa cells co-transfected with GFP-SKIP and Myc-SifA (red) **or** GFP-PLEKHM1 and Myc-SifA (red). Bars: (main) 10 μm. **g)** Pearson’s correlation coefficient was calculated for the indicated protein pairs in transfected cells as labeled. Data represent mean ± S.D. over three independent experiments where ~25–30 transfected cells were analyzed in each experiment (****, P < 0.0001; Student’s *t* test). **h)** Primary yeast cultures were seeded in SD/-leucine/-tryptophan broth from single colonies of *S*. *cerevisiae* AH109 strain co-transformed with indicated plasmids, and grown overnight at 30°C to saturation. The resulting cultures were diluted to approximately 0.1 OD (at 600 nm) in SD/-leucine/-tryptophan/-histidine broth and culture growth was monitored every 4 hr for 48 hr. **i)** Immunoblot of a GST pulldown assay using HEK293T cell lysates expressing FLAG-PLEKHM1 incubated with GST or GST tagged-SifA. Purified proteins were visualized by Ponceau S staining. **j)** Densitometric analysis of immunoblots of FLAG-PLEKHM1 or FLAG-SKIP pulldown with GST tagged-SifA (normalized to their respective input band intensity). **k)** qRT-PCR analysis to evaluate expression level of PLEKHM1 and SKIP in HeLa cells. **l)** Control siRNA- and SKIP siRNA-treated HeLa cells were infected with *Salmonella* for the indicated times and the number of CFU per well were determined and shown as dot plot. Data represent mean ± S.D. (n.s., not significant; ****, P < 0.0001; Student’s *t* test).
(TIF)

S11 Fig SifA-SKIP interaction is required for Vps41 recruitment to SCV membranes. **a and b)** Live-cell imaging was performed on control siRNA- or SKIP siRNA-treated HeLa cells infected with DsRed-expressing *Salmonella* (red) and co-transfected with plasmids encoding GFP-Vps41 and untagged-Arl8b. Time-lapse series were recorded 10 hr p.i., and still images correspond to movies shown as S12 and S14 Movies. Different panels represent the time-lapse series of the boxed area. Arrowheads indicate individual SCVs. Bars: (main) 10 μm; (insets) 5 μm. **c and d)** Representative confocal micrographs of HeLa cells treated with either control siRNA (**c**) or PLEKHM1 siRNA (**d**), and infected with DsRed-expressing *Salmonella* (red) followed by co-transfection with HA-Vps41 and Arl8b. Cells were fixed 10 hr p.i., and immunostaining was performed using anti-HA (green) and anti-LAMP1 (blue) antibodies. Insets depict higher magnification of the boxed areas (SCVs are indicated by arrowheads). Bars: (main) 10 μm; (insets) 5 μm. **e)** Lysates from HEK293T cells co-transfected with plasmids expressing FLAG-SKIP and either Myc-SifA (WT), Myc-SifA (L130D) or Myc-SifA (M131D) were immunoprecipitated using anti-FLAG antibodies-conjugated resins. The precipitates were resolved on SDS-PAGE and immunoblotted with indicated antibodies. **f and g)** Time-lapse microscopy was performed on HeLa cells infected with DsRed-expressing *Salmonella* strains *sifA/*pSifA (WT)-2HA (**f**) or *sifA/*pSifA (L130D)-2HA (**g**) and co-transfected with the plasmids expressing GFP-Vps41 and untagged-Arl8b. Time-lapse series were recorded 9 hr p.i., and still images correspond to movies shown as S16 and S18 Movies. Different panels represent the time-lapse series of the boxed area. Arrowheads indicate individual SCVs. Bars: (main) 10 μm; (insets) 5 μm.
(TIF)

S1 Movie Live-cell imaging of HeLa cells transfected with plasmid encoding GFP-Vps41, and infected with DsRed-expressing *Salmonella* (red). Time-lapse series were recorded at 9 hr p.i. with every image captured at an interval of 5 sec. Movie is shown at 5 frames/sec.
(MP4)

S2 Movie Extension, retraction, and collapse of Vps41-positive SIFs. Live-cell imaging of HeLa cells co-transfected with plasmids encoding GFP-Vps41 and untagged-Arl8b, and infected with DsRed-expressing *Salmonella* (red). Time-lapse series were recorded 9 hr p.i. with every image captured at an interval of 10 sec. Movie is shown at 5 frames/sec.
(MP4)

S3 Movie Live-cell imaging of Arl8b KO HeLa cells transfected with plasmid encoding GFP-Vps41, and infected with DsRed-expressing *Salmonella* (red). Time-lapse series were recorded at 10 hr p.i. with every image captured at an interval of 5 sec. Movie is shown at 5 frames/sec.
(MP4)

S4 Movie The movie shows the formation of extensive dynamic tubules (SIFs) in control siRNA treated HeLa cells expressing GFP-LAMP1 and infected with DsRed-expressing *Salmonella*. Time-lapse series were recorded at 9 hr p.i. with every image captured at an interval of 15 sec. Movie is shown at 5 frames/sec.
(MP4)

S5 Movie Absence of SIF formation in Vps41-depleted cells. The movie shows the absence of SIFs in Vps41 siRNA treated HeLa cells expressing GFP-LAMP1 and infected with DsRed-expressing *Salmonella*. Time-lapse series were recorded at 9 hr p.i. with every image captured at an interval of 15 sec. Movie is shown at 5 frames/sec.
(MP4)

S6 Movie Absence of SIF formation in Vps39-depleted cells. The movie shows the absence of SIFs in Vps39 siRNA treated HeLa cells expressing GFP-LAMP1 and infected with DsRed-expressing *Salmonella*. Time-lapse series were recorded at 9 hr p.i. with every image captured at an interval of 15 sec. Movie is shown at 5 frames/sec.
(MP4)

S7 Movie Accessibility of late-endocytic cargo to SCVs and SIFs in *Salmonella* infected HeLa cells. The movie shows that majority of SCVs and SIFs acquire the dextran-647 fluorescent tracer in the lumen of the compartment, thereby labeling both of these compartments in case of control siRNA treated HeLa cells infected with GFP-expressing *Salmonella*. Time-lapse series were recorded at 10 hr p.i. with every image captured at an interval of 15 sec. Movie is shown at 5 frames/sec.
(MP4)

S8 Movie Accessibility of late-endocytic cargo to SCVs is prohibited upon depletion of HOPS subunit-Vps41 in HeLa cells. The movie shows that in HeLa cells depleted of Vps41 expression and infected with GFP-expressing *Salmonella*, reduced interaction of SCVs with dextran 647-loaded endolysosomal compartments was observed. Time-lapse series were recorded at 9 hr p.i. with every image captured at an interval of 15 sec. Movie is shown at 5 frames/sec.
(MP4)

S9 Movie Accessibility of late-endocytic cargo to SCVs and SIFs in *Salmonella*-infected RAW264.7 cells. The movie shows that majority of SCVs and SIFs acquire the dextran-647 fluorescent tracer in the lumen of the compartment, thereby labeling both of these compartments in case of control shRNA transduced RAW264.7 cells infected with GFP-expressing *Salmonella*. Time-lapse series were recorded at 10 hr p.i. with every image captured at an interval of 3 sec. Movie is shown at 5 frames/sec.
(MP4)

S10 Movie Accessibility of late-endocytic cargo to SCVs is prohibited upon depletion of HOPS subunit Vps41 in RAW264.7 cells. The movie shows that in RAW264.7 cells depleted of Vps41 expression and infected with GFP-expressing *Salmonella*, reduced interaction of SCVs with dextran 647-loaded endolysosomal compartments was observed. Time-lapse series were recorded at 10 hr p.i. with every image captured at an interval of 3 sec. Movie is shown at 5 frames/sec.
(MP4)

S11 Movie Control siRNA treated HeLa cells were infected with DsRed-expressing *Salmonella* followed by transfection with plasmid encoding GFP-Vps41. Time-lapse series were recorded at 10 hr p.i. with every image captured at an interval of 5 sec. Movie is shown at 5 frames/sec.
(MP4)

S12 Movie Control siRNA treated HeLa cells were infected with DsRed-expressing *Salmonella* followed by co-transfection with plasmids encoding GFP-Vps41 and untagged-Arl8b. Time-lapse series were recorded at 10 hr p.i. with every image captured at an interval of 5 sec. Movie is shown at 5 frames/sec.
(MP4)

S13 Movie HOPS complex is recruited onto SCVs and SIFs in a SKIP-dependent manner. SKIP siRNA treated HeLa cells were infected with DsRed-expressing *Salmonella* followed by transfection with plasmid encoding GFP-Vps41. Time-lapse series were recorded at 9 hr p.i. with every image captured at an interval of 5 sec. Movie is shown at 5 frames/sec.
(MP4)

S14 Movie HOPS complex is recruited onto SCVs and SIFs in a SKIP-dependent manner. SKIP siRNA treated HeLa cells were infected with DsRed-expressing *Salmonella* followed by co-transfection with plasmids encoding GFP-Vps41 and untagged-Arl8b. Time-lapse series were recorded at 10 hr p.i. with every image captured at an interval of 5 sec. Movie is shown at 5 frames/sec.
(MP4)

S15 Movie Live-cell imaging was performed on HeLa cells infected with DsRed-expressing *Salmonella* strain *sifA/*pSifA (WT)-2HA and transfected with plasmid expressing GFP-Vps41. Time-lapse series were recorded at 10 hr p.i. with every image captured at an interval of 5 sec. Movie is shown at 5 frames/sec.
(MP4)

S16 Movie Live-cell imaging was performed on HeLa cells infected with DsRed-expressing *Salmonella* strain *sifA/*pSifA (WT)-2HA and co-transfected with the plasmids expressing GFP-Vps41 and untagged-Arl8b. Time-lapse series were recorded at 9 hr p.i. with every image captured at an interval of 5 sec. Movie is shown at 5 frames/sec.
(MP4)

S17 Movie Live-cell imaging was performed on HeLa cells infected with DsRed-expressing *Salmonella* strain *sifA/*pSifA (L130D)-2HA and transfected with plasmid expressing GFP-Vps41. Time-lapse series were recorded at 10 hr p.i. with every image captured at an interval of 5 sec. Movie is shown at 5 frames/sec.
(MP4)

S18 Movie Live-cell imaging was performed on HeLa cells infected with DsRed-expressing *Salmonella* strain *sifA/*pSifA (L130D)-2HA and co-transfected with the plasmids expressing GFP-Vps41 and untagged-Arl8b. Time-lapse series were recorded at 9 hr p.i. with every image captured at an interval of 5 sec. Movie is shown at 5 frames/sec.
(MP4)
