# Supplementary figures and images for: Salmonella exploits the host endolysosomal tethering factor HOPS complex to promote its intravacuolar replication
Source: PLoS Pathog. 2017 Oct 30;13(10):e1006700. doi: 10.1371/journal.ppat.1006700 (PMC5679646; doi:10.1371/journal.ppat.1006700)

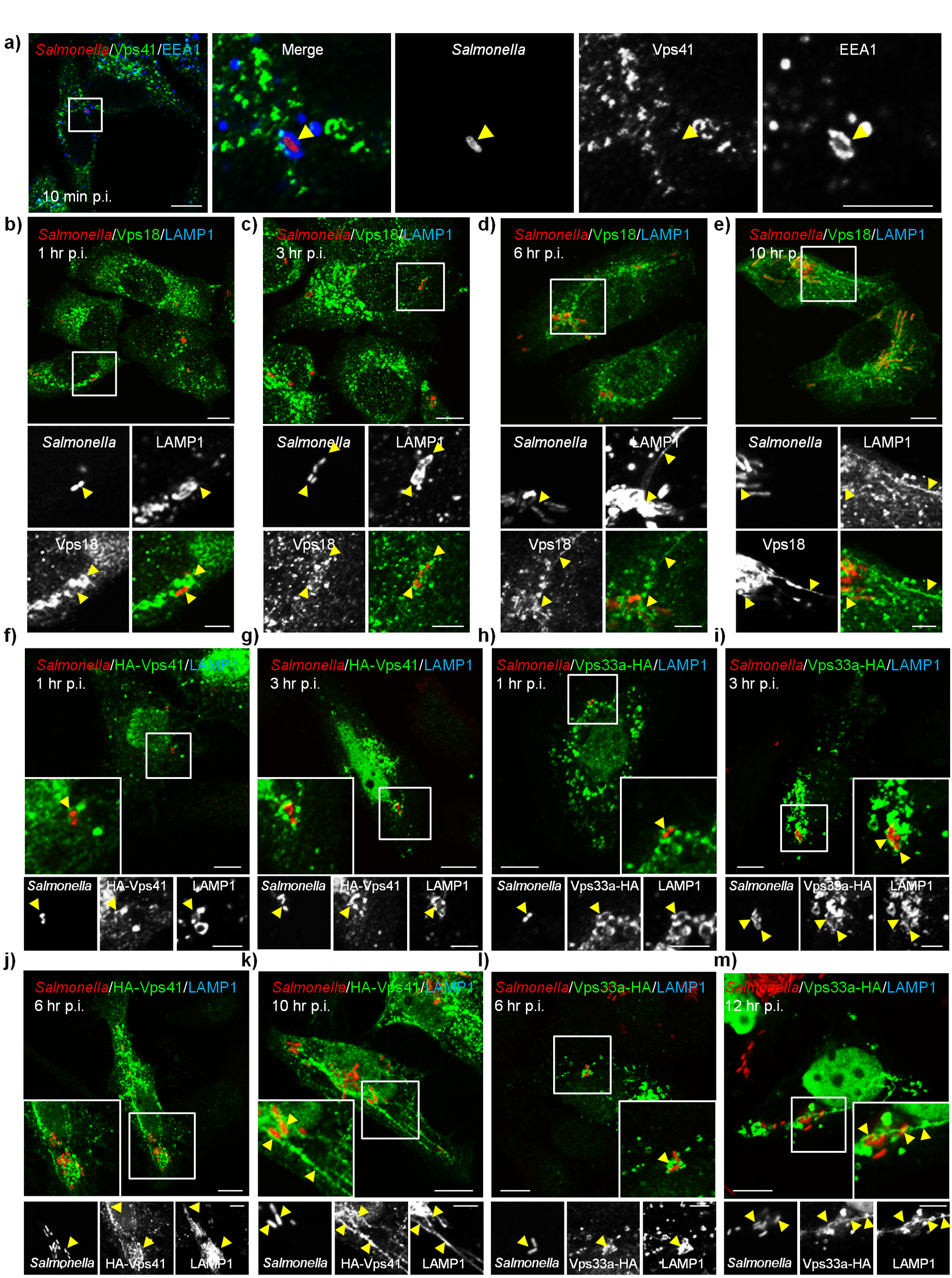

Supplement: S1 Fig — a) Representative confocal micrographs of HeLa cells infected with DsRed-expressing Salmonella (red). At 10 min p.i., cells were fixed and stained for endogenous Vps41 (green) and EEA1 (blue). Different panels represent a higher magnification of the boxed areas, showing absence of Vps41 but presence of EEA1 around SCVs (marked by arrowheads) at this time point of infection. b-e) Representative confocal micrographs of HeLa cells infected with DsRed-expressing Salmonella (red). At different times after infection, cells were fixed and stained for endogenous Vps18 (green) and LAMP1 (blue, shown only in inset). Insets depict recruitment of Vps18 on SCVs and SIFs as marked by arrowheads. f-m) Representative confocal micrographs of HA-Vps41 or Vps33a-HA transfected HeLa cells infected with DsRed-expressing Salmonella (red). At different times after infection, cells were fixed and stained using anti-HA (green) and anti-LAMP1 (blue, shown only in inset) antibodies. Insets depict recruitment of epitope-tagged HOPS subunits on SCVs and SIFs as marked by arrowheads. Bars: (main) 10 μm; (insets) 5 μm. (TIF) [file ppat.1006700.s001.tif]

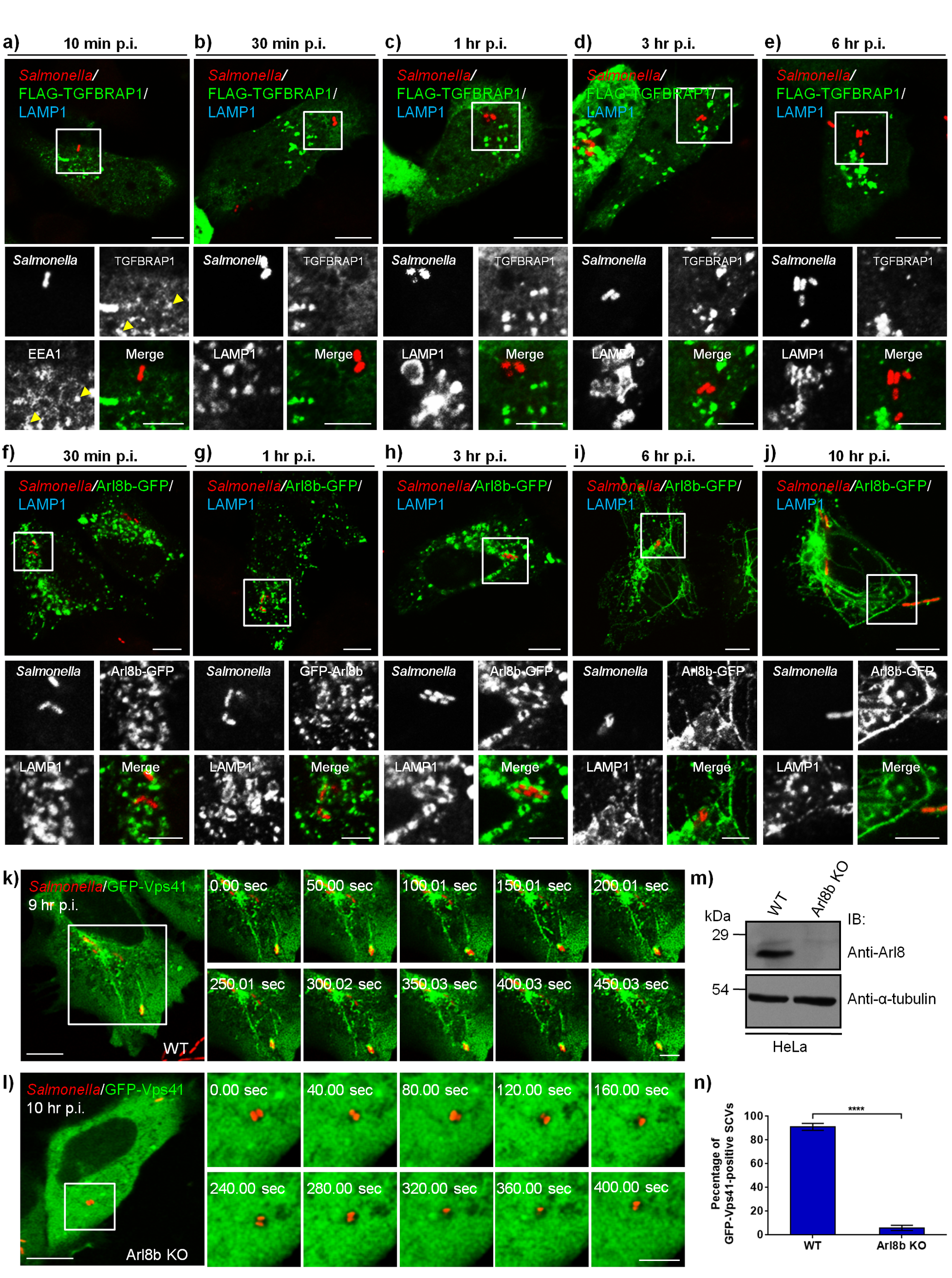

Supplement: S2 Fig — a-e) Representative confocal micrographs of FLAG-TGFBRAP1 transfected HeLa cells infected with DsRed-expressing Salmonella (red). At different times after infection (as indicated), cells were fixed and stained using anti-FLAG (green) and anti-EEA1 (a, blue) or anti-LAMP1 (b-e, blue, shown only in inset) antibodies. Arrowheads in inset from panel (a) depict colocalization of TGFBRAP1 with EEA1. f-j) Representative confocal micrographs of Arl8b-GFP transfected HeLa cells infected with DsRed-expressing Salmonella (red). At different times after infection (as indicated), cells were fixed and stained using anti-LAMP1 (blue, shown only in inset) antibody. Insets depict higher magnification of boxed areas. Bars: (main) 10 μm; (insets) 5 μm. k and l) Time-lapse microscopy of WT or CRISPR/Cas9 Arl8b KO HeLa cells transfected with plasmid encoding GFP-Vps41, and infected with Salmonella expressing DsRed (red). Time-lapse series were recorded at the indicated times p.i., and still images correspond to movies shown as S1 and S3 Movies. Bars: (main) 10 μm; (insets) 5 μm. m) WT- and CRISPR/Cas9 Arl8b KO-HeLa cell lysates were immunoblotted with anti-Arl8 antibody for assessing the knockdown efficiency and with anti-α-tubulin antibody as a loading control. n) Quantification of GFP-Vps41-positive SCVs in WT- and Arl8b KO-HeLa cells. Data represent mean ± S.D. over three independent experiments at 10 hr p.i. where 100 SCVs were counted in each experiment (****, P < 0.0001; Student’s t test). (TIF) [file ppat.1006700.s002.tif]

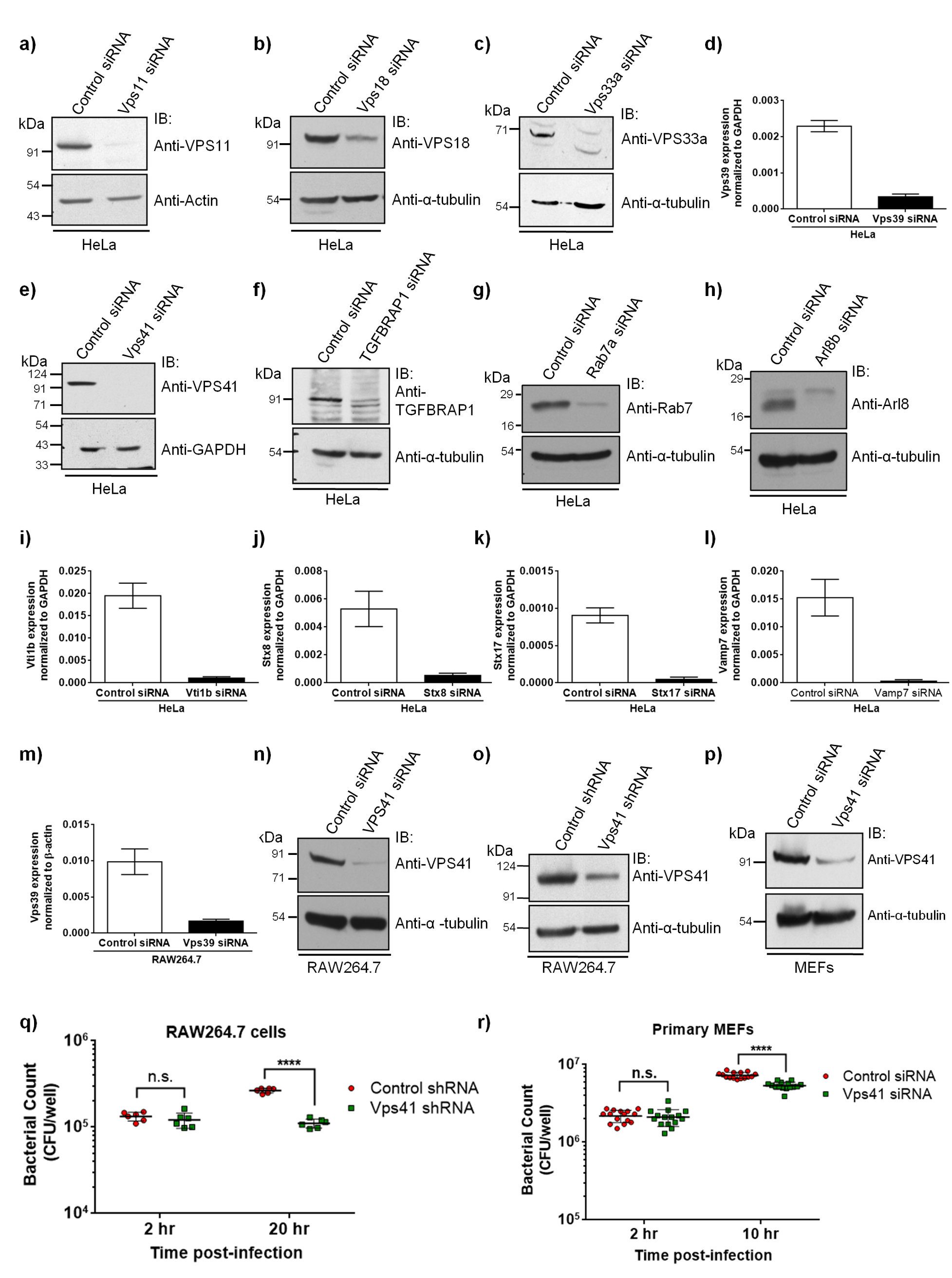

Supplement: S3 Fig — a-p) Western blotting or qRT-PCR analysis of different cell types transfected with indicated siRNA or shRNA was performed to measure the gene silencing efficiency. q and r) Intracellular replication assay. RAW264.7 (q) or primary MEF cells (r) treated with indicated shRNA or siRNA, and infected with Salmonella were harvested at indicated times p.i. The number of CFU per well were determined and shown as dot plot. Data represent mean ± S.D. (n.s., not significant; ****, P < 0.0001; Student’s t test). (TIF) [file ppat.1006700.s003.tif]

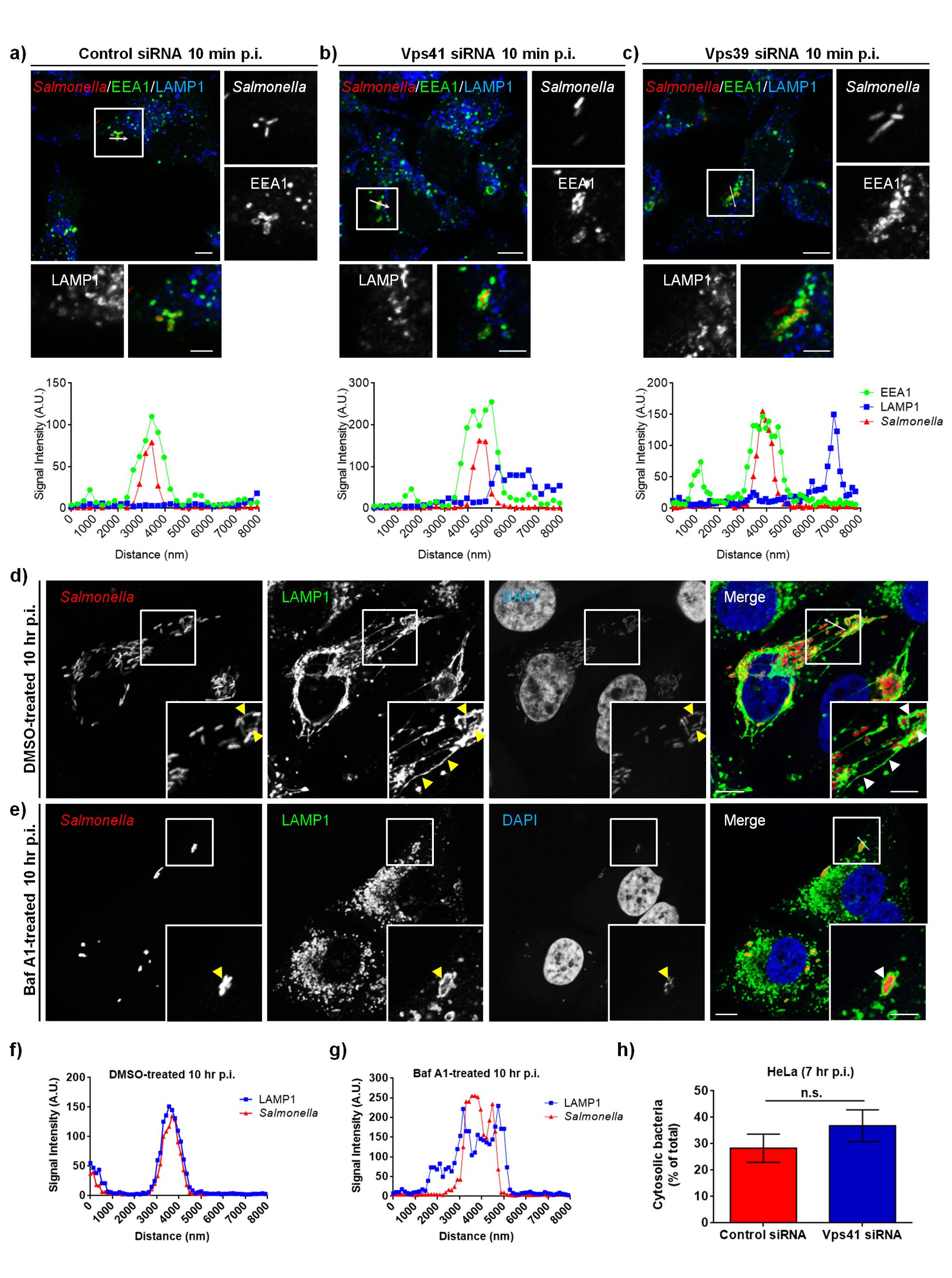

Supplement: S4 Fig — a-c) Representative confocal micrographs of control siRNA-, Vps41 siRNA- or Vps39 siRNA-treated HeLa cells infected with DsRed-expressing Salmonella (red). At 10 min p.i., cells were fixed and stained for early endosomes marker, EEA1 (green) and LAMP1 (blue). Insets depict higher magnification of the boxed areas showing localization of different markers on the SCVs. Shown below the image is the intensity scan profile to visualize colocalization of Salmonella (red) with EEA1 (green) and LAMP1 (blue). d and e) HeLa cells pre-treated with either DMSO (vehicle control) or Bafilomycin A1 (Baf A1) (50 nM) overnight were infected with DsRed-expressing Salmonella (red). At 10 hr p.i., cells were fixed and immunostaining for LAMP1 (green) was performed. The nuclei were stained using DAPI (blue). Insets depict higher magnification of the boxed areas showing localization of different markers on the SCVs. Bars: (main) 10 μm; (insets) 5 μm. f and g) The intensity scan profile to visualize colocalization of Salmonella (red) with LAMP1 (blue) in DMSO or Baf A1 treated HeLa cells is shown. h) Chloroquine (CHQ) resistance assay was performed to quantify the percentage of cytosolic bacteria in total population upon Vps41 silencing. HeLa cells seeded in a 24-well plate were transfected with control- or Vps41-siRNA, and infected with Salmonella. After 6 hr p.i., two wells were incubated with CHQ and gentamicin (CHQ-resistant bacteria, cytosolic bacteria) and two wells were incubated with gentamicin only (total bacteria) for 1 hr. At the end of 7 hr p.i., cells were harvested and the number of CFU per well were determined and the percentage of cytosolic bacteria proliferation was calculated as the ratio of CFU obtained at 7 hr p.i. in CHQ + gentamicin treated wells/CFU obtained at 7 hr p.i. in gentamicin alone treated wells. Data represent mean ± S.D. from three independent experiments (n.s., not significant; Student’s t test). (TIF) [file ppat.1006700.s004.tif]

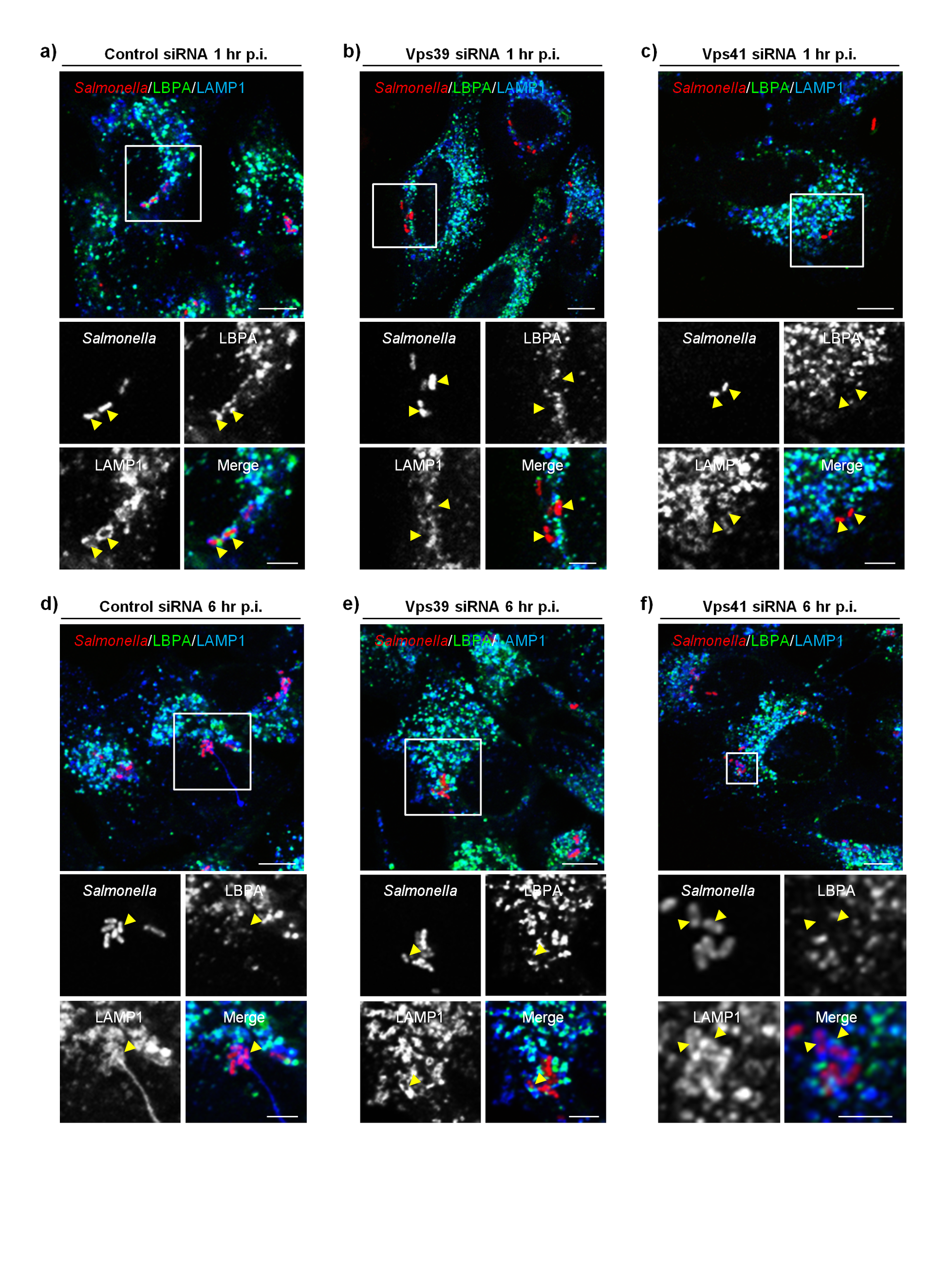

Supplement: S5 Fig — a-f) Representative confocal micrographs of control siRNA-, Vps39 siRNA- or Vps41 siRNA-treated HeLa cells infected with DsRed-expressing Salmonella (red). At 1 hr (a-c) and 6 hr (d-f) p.i., cells were fixed and stained for LBPA (green) and LAMP1 (blue). Insets depict higher magnification of the boxed areas showing localization of different markers on the SCVs. Bars: (main) 10 μm; (insets) 5 μm. (TIF) [file ppat.1006700.s005.tif]

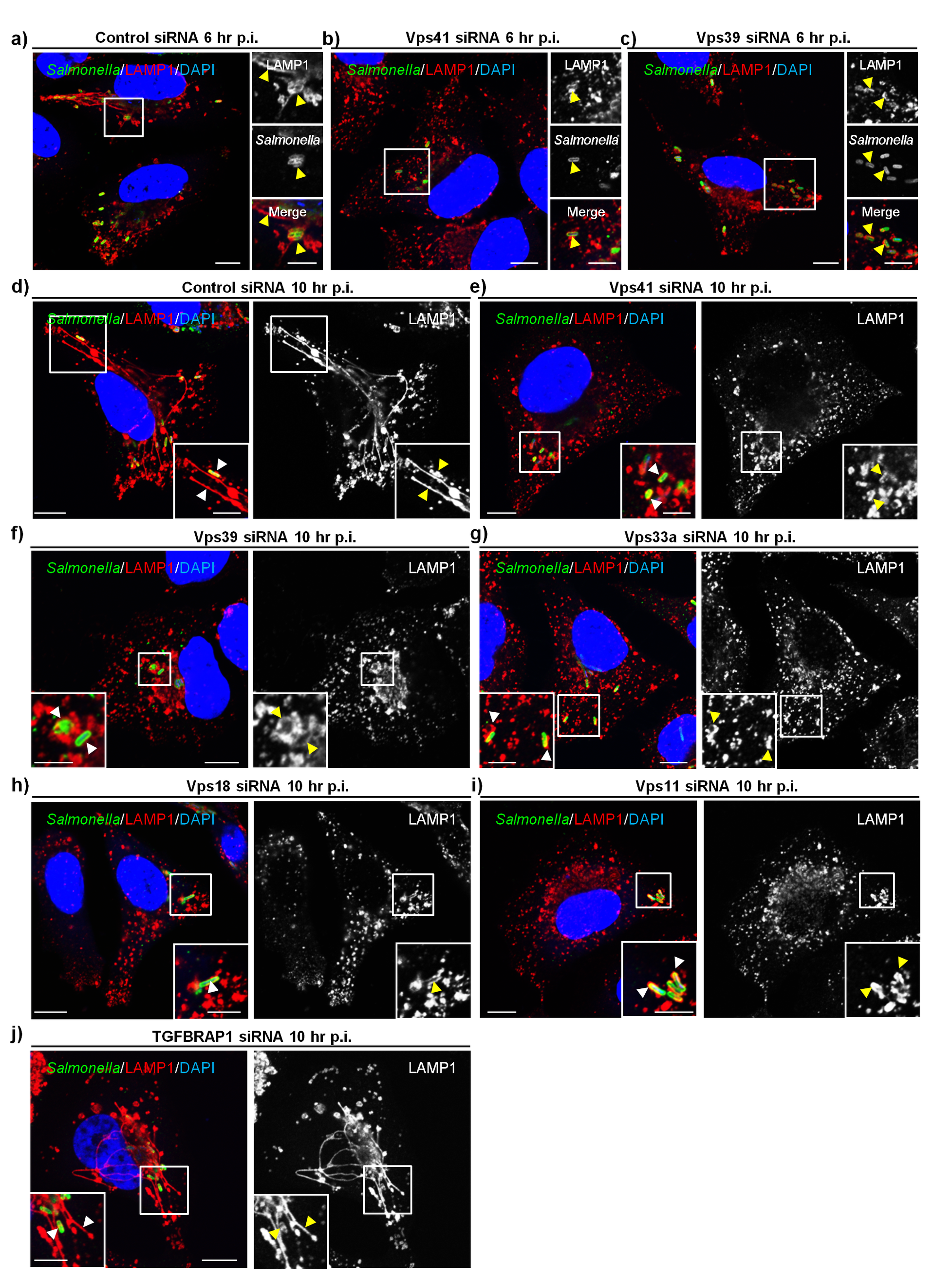

Supplement: S6 Fig — a-j) Representative confocal micrographs of control siRNA (a and d)-, HOPS subunits specific siRNA (b, c, and e-i)- or TGFBRAP1 siRNA (j)-transfected HeLa cells and infected with Salmonella. At different times after infection (as indicated), cells were fixed and immunostained with antibodies to Salmonella (green) and LAMP1 (red). The nuclei were stained using DAPI (blue). Insets represent a higher magnification of the boxed areas with arrowheads depicting LAMP1 localization on individual SCVs. Bars: (main) 10 μm; (insets) 5 μm. (TIF) [file ppat.1006700.s006.tif]

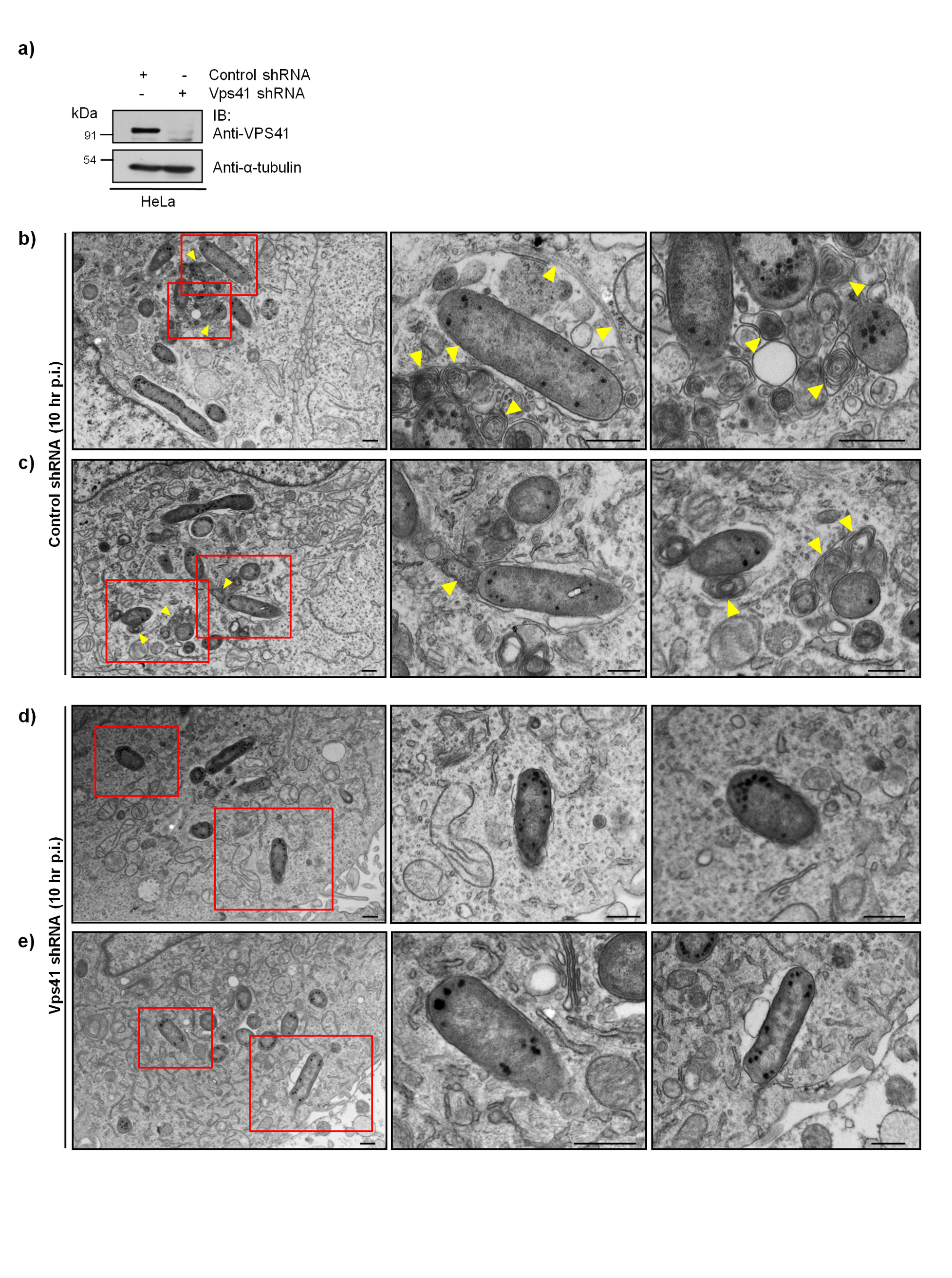

Supplement: S7 Fig — a) Lysates of control shRNA or Vps41 shRNA transduced HeLa cells were immunoblotted with anti-Vps41 antibody for assessing the knockdown efficiency and with anti-α-tubulin antibody as the loading control. b-e) Representative TEM images of control shRNA (b and c) and Vps41 shRNA (d and e) transduced HeLa cells infected with Salmonella for 10 hr. Higher magnification of multiple SCVs interacting with late endosomes and lysosomes in control shRNA transduced HeLa cells are shown (marked by arrowheads). Arrowheads indicate SIF formation in inset of panel (b). Bar: 500 nm. (TIF) [file ppat.1006700.s007.tif]

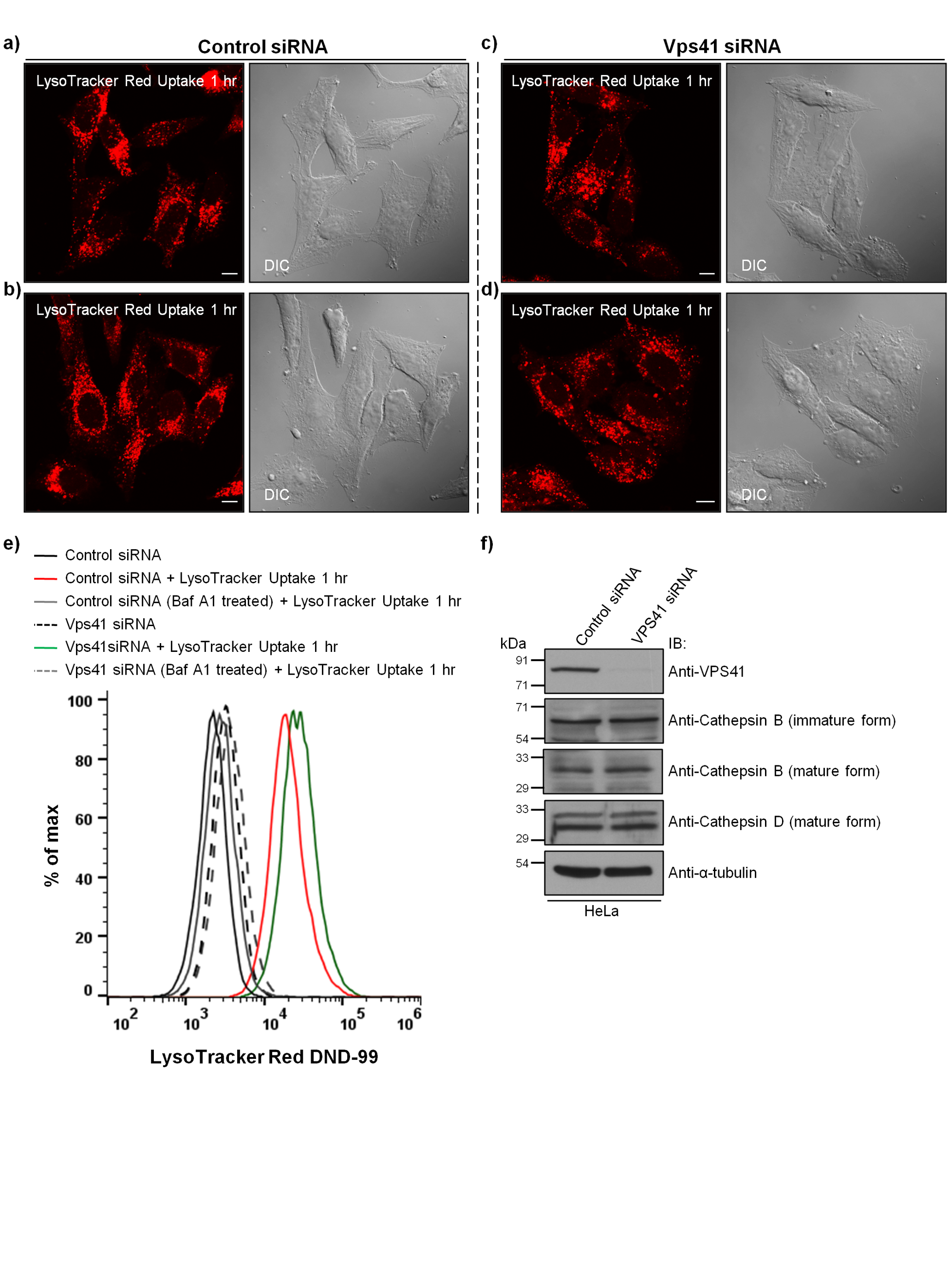

Supplement: S8 Fig — a-d) Representative confocal micrographs of control- and Vps41-siRNA treated HeLa cells incubated with LysoTracker Red (100 nM) for 1 hr. Bar: 10 μm. e) Control siRNA- and Vps41 siRNA-transfected HeLa cells were incubated with DMSO (vehicle control) or with Baf A1 incubated for 1 hr. After 1 hr, LysoTracker Red (100 nM) uptake was performed for 1 hr. At the end of the internalization period, cells were washed and fluorescence was determined by flow cytometry. f) Lysates from control- and Vps41-siRNA treated HeLa cells were resolved by SDS-PAGE and immunoblotted with indicated antibodies by Western blotting. (TIF) [file ppat.1006700.s008.tif]

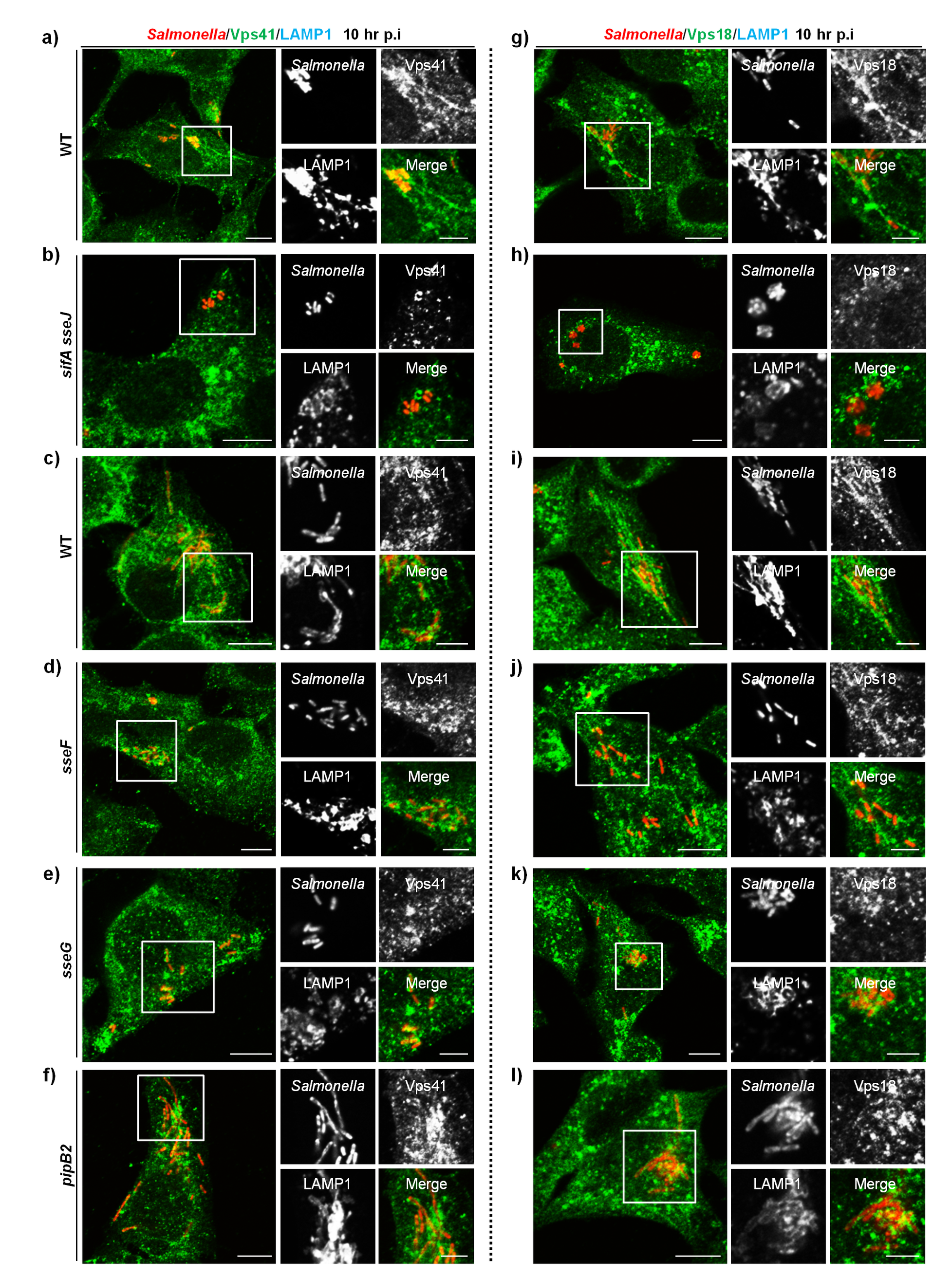

Supplement: S9 Fig — a-l) HeLa cells were infected with DsRed expressing-wild-type (WT) strain of Salmonella (NCTC 12023 (a and g) and SL1344 (c and i) or sifA sseJ (b and h), sseF (d and j), sseG (e and k), and pipB2 (f and l) strains. Cells were fixed at 10 hr p.i., and co-stained with anti-Vps41 (green, a-f) or anti-Vps18 (green, g-l) and anti-LAMP1 (blue, shown only in inset) antibodies. Different panels represent a higher magnification of the boxed areas. Bars: (main) 10 μm; (insets) 5 μm. (TIF) [file ppat.1006700.s009.tif]

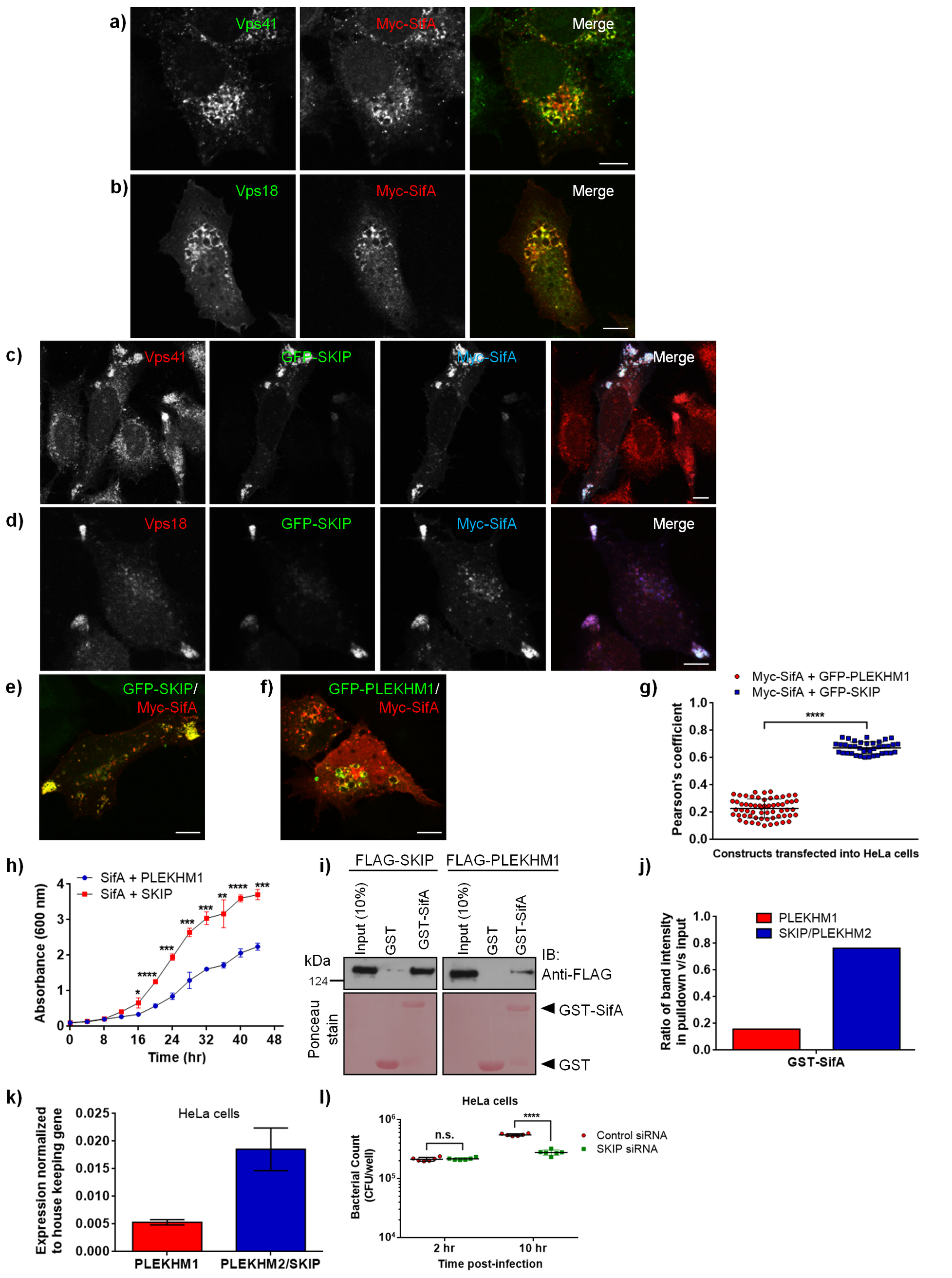

Supplement: S10 Fig — a-d) Representative confocal micrographs of HeLa cells transfected with Myc-SifA alone (red), or co-transfected with Myc-SifA (blue) and GFP-SKIP (green). Cells were fixed and stained with antibodies against Vps41 (red, a and c) and Vps18 (red, b and d). Bars: 10 μm. e and f) Representative confocal micrographs of HeLa cells co-transfected with GFP-SKIP and Myc-SifA (red) or GFP-PLEKHM1 and Myc-SifA (red). Bars: (main) 10 μm. g) Pearson’s correlation coefficient was calculated for the indicated protein pairs in transfected cells as labeled. Data represent mean ± S.D. over three independent experiments where ~25–30 transfected cells were analyzed in each experiment (****, P < 0.0001; Student’s t test). h) Primary yeast cultures were seeded in SD/-leucine/-tryptophan broth from single colonies of S. cerevisiae AH109 strain co-transformed with indicated plasmids, and grown overnight at 30°C to saturation. The resulting cultures were diluted to approximately 0.1 OD (at 600 nm) in SD/-leucine/-tryptophan/-histidine broth and culture growth was monitored every 4 hr for 48 hr. i) Immunoblot of a GST pulldown assay using HEK293T cell lysates expressing FLAG-PLEKHM1 incubated with GST or GST tagged-SifA. Purified proteins were visualized by Ponceau S staining. j) Densitometric analysis of immunoblots of FLAG-PLEKHM1 or FLAG-SKIP pulldown with GST tagged-SifA (normalized to their respective input band intensity). k) qRT-PCR analysis to evaluate expression level of PLEKHM1 and SKIP in HeLa cells. l) Control siRNA- and SKIP siRNA-treated HeLa cells were infected with Salmonella for the indicated times and the number of CFU per well were determined and shown as dot plot. Data represent mean ± S.D. (n.s., not significant; ****, P < 0.0001; Student’s t test). (TIF) [file ppat.1006700.s010.tif]

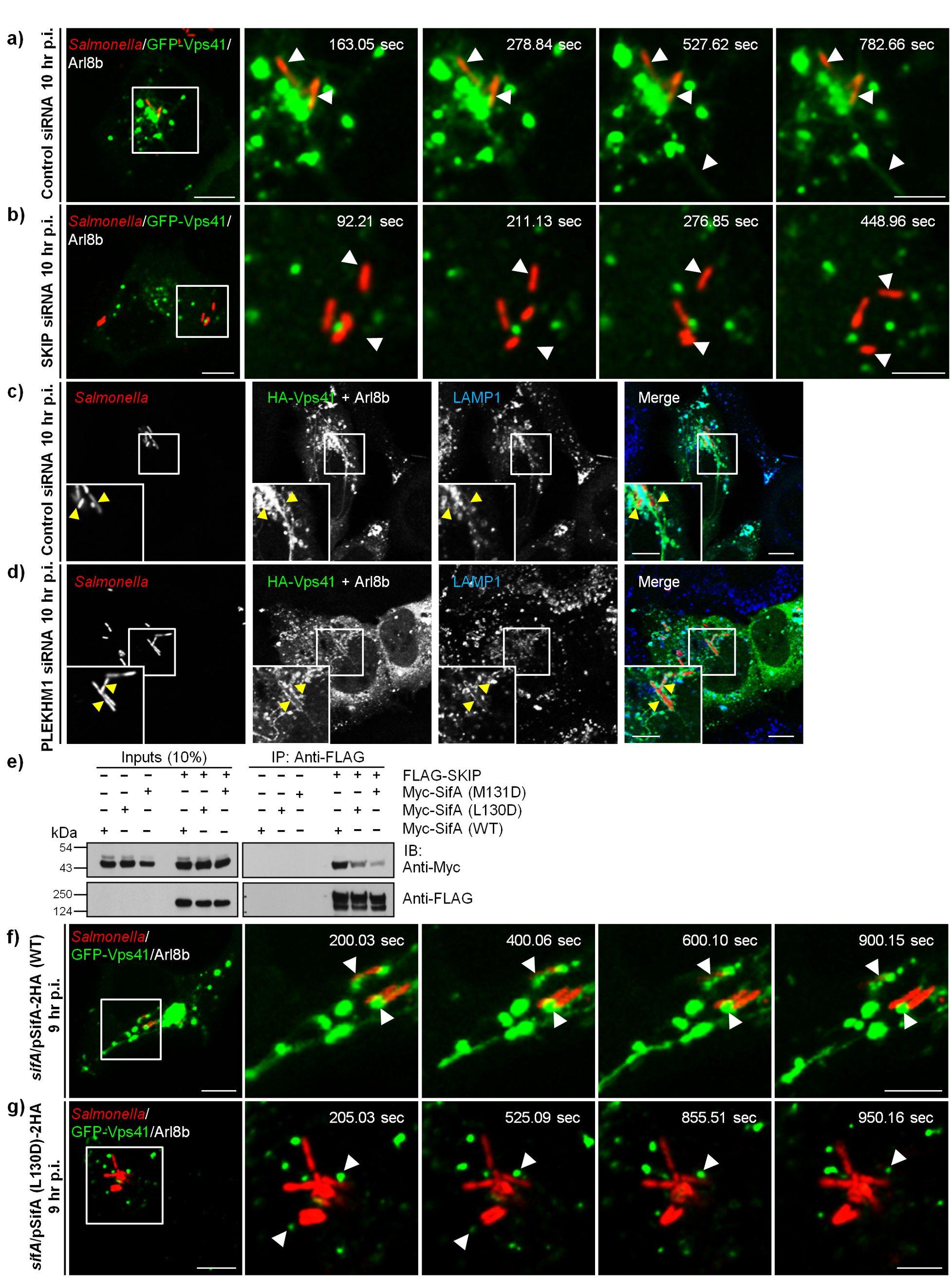

Supplement: S11 Fig — a and b) Live-cell imaging was performed on control siRNA- or SKIP siRNA-treated HeLa cells infected with DsRed-expressing Salmonella (red) and co-transfected with plasmids encoding GFP-Vps41 and untagged-Arl8b. Time-lapse series were recorded 10 hr p.i., and still images correspond to movies shown as S12 and S14 Movies. Different panels represent the time-lapse series of the boxed area. Arrowheads indicate individual SCVs. Bars: (main) 10 μm; (insets) 5 μm. c and d) Representative confocal micrographs of HeLa cells treated with either control siRNA (c) or PLEKHM1 siRNA (d), and infected with DsRed-expressing Salmonella (red) followed by co-transfection with HA-Vps41 and Arl8b. Cells were fixed 10 hr p.i., and immunostaining was performed using anti-HA (green) and anti-LAMP1 (blue) antibodies. Insets depict higher magnification of the boxed areas (SCVs are indicated by arrowheads). Bars: (main) 10 μm; (insets) 5 μm. e) Lysates from HEK293T cells co-transfected with plasmids expressing FLAG-SKIP and either Myc-SifA (WT), Myc-SifA (L130D) or Myc-SifA (M131D) were immunoprecipitated using anti-FLAG antibodies-conjugated resins. The precipitates were resolved on SDS-PAGE and immunoblotted with indicated antibodies. f and g) Time-lapse microscopy was performed on HeLa cells infected with DsRed-expressing Salmonella strains sifA/pSifA (WT)-2HA (f) or sifA/pSifA (L130D)-2HA (g) and co-transfected with the plasmids expressing GFP-Vps41 and untagged-Arl8b. Time-lapse series were recorded 9 hr p.i., and still images correspond to movies shown as S16 and S18 Movies. Different panels represent the time-lapse series of the boxed area. Arrowheads indicate individual SCVs. Bars: (main) 10 μm; (insets) 5 μm. (TIF) [file ppat.1006700.s011.tif]
